# Conceptualising a model to guide nursing and midwifery in the community guided by an evidence review

**DOI:** 10.1186/s12912-017-0225-3

**Published:** 2017-06-29

**Authors:** Patricia Leahy-Warren, Helen Mulcahy, Lazelle Benefield, Colin Bradley, Alice Coffey, Ann Donohoe, Serena Fitzgerald, Tim Frawley, Elizabeth Healy, Maria Healy, Marcella Kelly, Bernard McCarthy, Kathleen McLoughlin, Catherine Meagher, Rhona O’Connell, Aoife O’Mahony, Gillian Paul, Amanda Phelan, Diarmuid Stokes, Jessica Walsh, Eileen Savage

**Affiliations:** 1 School of Nursing & Midwifery, Brookfield health Sciences Complex, University College, Cork, Ireland; 2 University of Oklahoma Health Sciences Centre, Oklahoma City, USA; 3 Department of General Practice, University College, Cork, Ireland; 4 School of Health Sciences, University College, Dublin, Ireland; 5 HSE Cork South Lee Public Health Nursing, Cork, Ireland; 6 School of Nursing and Midwifery, Queen’s University Belfast, Belfast, Northern Ireland; 7 School of Nursing & Midwifery, NUI Galway, Galway, Ireland; 8 Health Sciences Library, University College, Dublin, Ireland

**Keywords:** Conceptual model, Nursing, Midwifery, Community, Primary care, Scoping review

## Abstract

**Background:**

Successful models of nursing and midwifery in the community delivering healthcare throughout the lifespan and across a health and illness continuum are limited, yet necessary to guide global health services. Primary and community health services are the typical points of access for most people and the location where most care is delivered. The scope of primary healthcare is complex and multifaceted and therefore requires a practice framework with sound conceptual and theoretical underpinnings.

The **aim** of this paper is to present a conceptual model informed by a scoping evidence review of the literature.

**Methods:**

A scoping evidence review of the literature was conducted using the Preferred Reporting Items for Systematic Reviews and Meta-Analysis (PRISMA) statement. Databases included CINAHL, MEDLINE, PsycINFO and SocINDEX using the EBSCO platform and the Cochrane Library using the keywords: model, nursing, midwifery, community, primary care. Grey literature for selected countries was searched using the Google ‘advanced’ search interface. Data extraction and quality appraisal for both empirical and grey literature were conducted independently by two reviewers. From 127 empirical and 24 non-empirical papers, data extraction parameters, in addition to the usual methodological features, included: the nature of nursing and midwifery; the population group; interventions and main outcomes; components of effective nursing and midwifery outcomes.

**Results:**

The evidence was categorised into six broad areas and subsequently synthesised into four themes. These were not mutually exclusive: (1) Integrated and Collaborative Care; (2) Organisation and Delivery of Nursing and Midwifery Care in the Community; (3) Adjuncts to Nursing Care and (4) Overarching Conceptual Model. It is the latter theme that is the focus of this paper. In essence, the model depicts a person/client on a lifespan and preventative-curative trajectory. The health related needs of the client, commensurate with their point position, relative to both trajectories, determines the nurse or midwife intervention. Consequently, it is this need, that determines the discipline or speciality of the nurse or midwife with the most appropriate competencies.

**Conclusion:**

Use of a conceptual model of nursing and midwifery to inform decision-making in primary/community based care ensures clinical outcomes are meaningful and more sustainable. Operationalising this model for nursing and midwifery in the community demands strong leadership and effective clinical governance.

**Electronic supplementary material:**

The online version of this article (doi:10.1186/s12912-017-0225-3) contains supplementary material, which is available to authorized users.

## Background

The delivery of an effective, efficient, safe, fair and equitable healthcare service is a focus both internationally and within the Irish healthcare context [1–4]. Coupled with this need is the move toward primary care and primary healthcare delivery that demands the use of an effective, efficient and cost effective community based model of care [3, 4]. There are ideals inherent within primary healthcare which encompass a person-centred focus involving collaborative, comprehensive and coordinated service delivery, that is family and community centred [1, 2]. Recognition of these ideals serve as the starting point from the first point of access [1, 2, 5], which is frequently considered as primary care, occurring outside of acute or long term care and within a community setting.

The delivery of primary healthcare involves its re-orientation, moving from the acute secondary to primary and community sector, emphasising a preventative focus of care [6]. This re-orientation of service delivery needs to address the needs of service users and the demands of healthcare providers, in a shared collaborative, empowering manner [5, 6]. Thus, the inherent ethos within this focus of care provision, is working with the community population as a client [1, 2, 5]. A shift was articulated by Mason and Clarke [7] emphasising departure from ‘professional health care provider’ to ‘enabler and facilitator of health’, and of partnership [8]. This form of service delivery requires a person-centred mode of care delivery, with shared partnership and collaboration in decision making while recognising clients as being central to this process [1, 2].

The development of an effective nursing and midwifery model in the community, as a first step, requires a review of the evidence regarding what models are currently available, with consideration as to how they could best inform the organisation and delivery of community services and practice. According to Ulrich et al. [9], the need for evidence-based design in healthcare is promising in terms of benefiting service users, healthcare professionals, and healthcare organizations. Therefore, the purpose of this paper is to report on a scoping evidence review of the literature to identify existing models of community nursing and midwifery, and to identify evidence that could inform the development of an optimal model.

## Methods

The aim of the scoping review was to identify and appraise literature regarding existing models of community nursing and midwifery that could inform the development of an organisational model for service delivery within these contexts. A systematic search was conducted of online databases CINAHL, MEDLINE, PsycINFO and SocINDEX. The Cochrane Library was searched for studies about community nursing or midwifery using the PICOCS framework (Table 1) to develop an initial search strategy and to support selection criteria [10]. In addition, the grey literature for selected countries (USA, UK, Netherlands, Canada, Australia and Ireland) was searched using the Google ‘advanced’ search interface.

**Table 1:** PICOS framework guiding selection criteria

Population:	The whole population including (but not exclusively): new mothers, infants and children, adolescents, children with complex needs, including disabilities, older adults, adults with chronic illnesses, adults with mental health issues, people in need of palliative care, vulnerable populations including minority groups, migrants and travelling communities, victims of/those at risk of domestic violence or sexual abuse, school going children and adolescents.
Interventions:	Any intervention that manages nursing and midwifery care in the community in comparison to no intervention/usual care.
Comparator:	No intervention/usual or standard care or service delivery/another model or programme of care or integration.
Outcomes:	Any measures of patient centred, process, service or economic outcomes. Any measures/reporting of barriers and enablers relating to implementation of models of community nursing or midwifery. Any recommendations regarding education, research, service delivery, policy relating to community nursing or midwifery.
Contexts:	Community based nursing and midwifery services delivering care across a wide variety of settings including GP Practice, home, schools, community and health centres. Added post-hoc: Countries classified as high human development level (UNDP, 2014),
Studies:	Systematic reviews of reviews, meta-analysis, systematic reviews and randomised controlled studies, meta-synthesis, narrative reviews (Narrative reviews and meta-synthesis were later excluded). In addition, peer reviewed papers, evidence based policy documents or mixed method studies reporting on the implementation or evaluation of programmes/models in Ireland, United Kingdom, Netherlands, Australia, America and Canada. Published between November 1st 2005 and 31st October 2015 (later reduced to 1st November 2010 – 31st October 2015). Written in the English language.

**Fig. 1:**
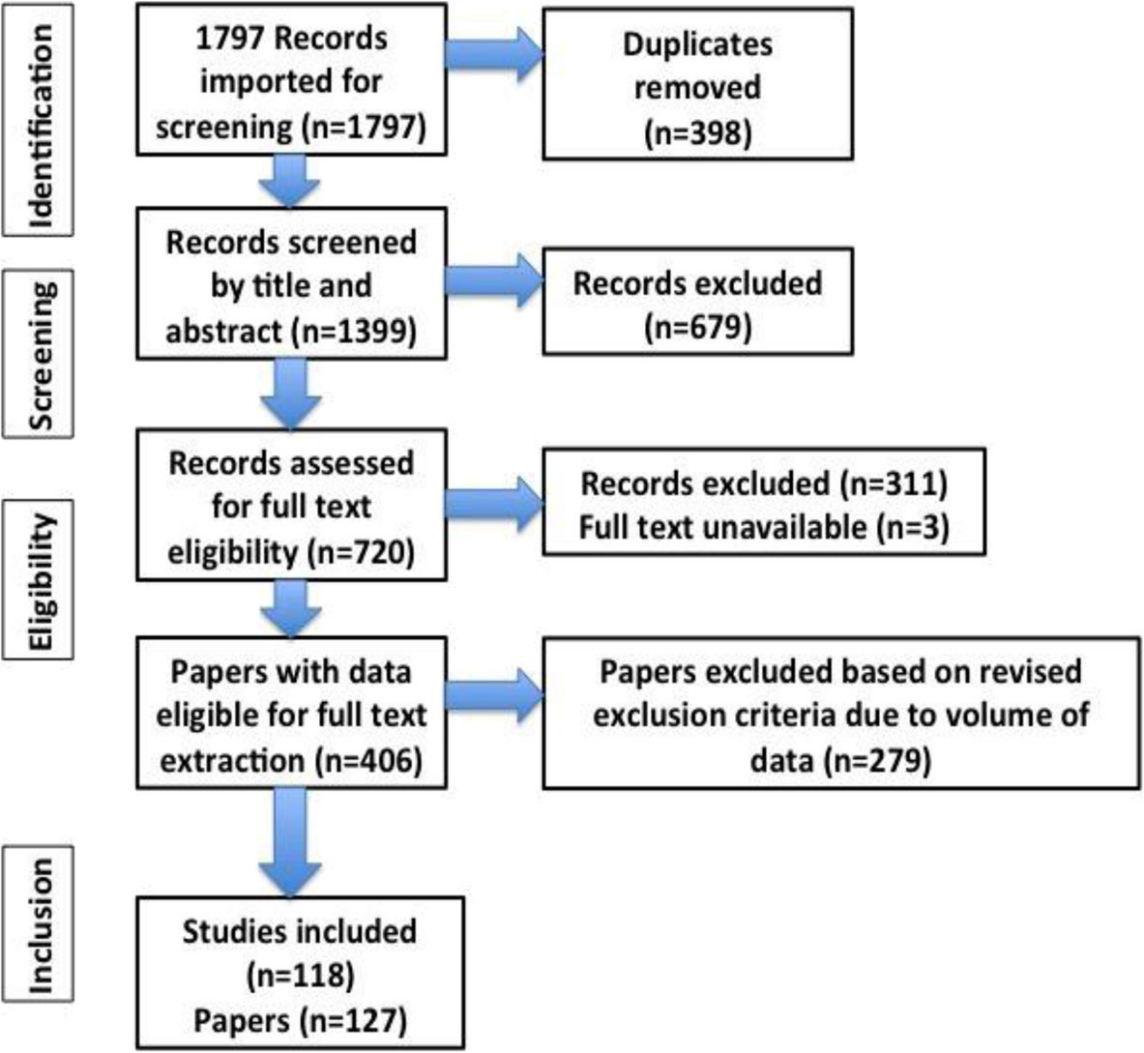
Prisma flowchart here

Studies were stratified and grouped according to study type i.e. RCTs and systematic reviews, meta-analyses or meta-reviews. A quality assessment of each study was conducted using The Cochrane Risk of Bias Tool for Randomised Controlled Trials [11, 12] as recommended by Zeng et al. [13] and the AMSTAR for systematic reviews/meta-analyses and meta-reviews, as proposed by Shea et al. [14, 15]. 1797 records were identified, 720 full text papers were reviewed and a total of 118 studies were included in the review (see Fig. 1).

For each study, a data extraction matrix was completed identifying thirteen key features of the study including an overview of the model, population group and size, the health condition of focus, the healthcare context, nursing/and or midwifery disciplines involved, outcomes assessed and components of the intervention (Additional file 1: Appendix 1).

To facilitate the review and analysis of this large number of studies (*n* = 118) it was necessary to identify distinct (but not mutually exclusive) categories based on the primary aim of each research paper. This resulted in the emergence of broad category areas and associated sub-themes.

## Results

**Fig. 2:**
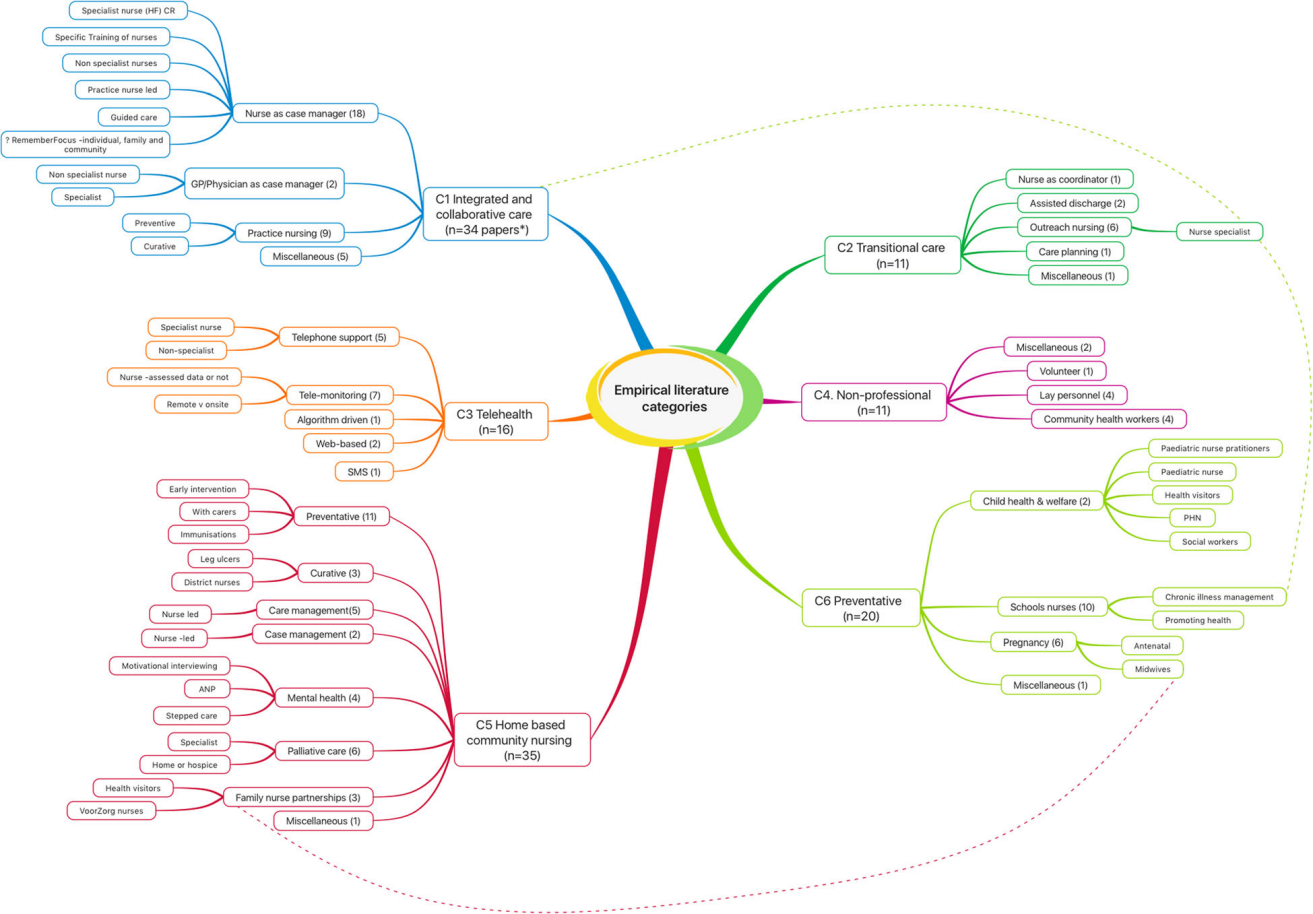
Visual overview of categories of empirical literature. Legend *n = papers rather than studies

No single overarching model of nursing and midwifery practice in the community emerged from the literature. What did emerge were evidence-based dimensions that can inform an effective model for the future. The empirical findings were organised into six categories: Integrated and Collaborative Care (*n* = 33); Home Based Community Nursing (*n* = 32); Telehealth (*n* = 15); Transitional Care (*n* = 9); Non-Professional (*n* = 10) and Preventative (*n* = 18). A visual overview is presented (Fig. 2).

These categories were analysed and condensed into four synthesised themes, namely: Integrated and Collaborative Care, Organisation and Delivery of Nursing and Midwifery Care in the Community, Adjuncts to Nursing and Midwifery Care, and an Overarching Model. Essentially these themes represent the philosophical underpinning necessary to inform a proposed model for nursing and midwifery in the community.

### Theme 1: integrated and collaborative care

In high income countries, healthcare systems have been guided by international policies underpinned by principles of public health and primary care. Inherent within this philosophy of care is the need for integration and closer collaboration between primary and secondary services and teams [16, 17]. Integrated care necessitates the inclusion of collaborative links between all sectors of the health service. Such links assist in seamless transitions for people with health issues and ensures the well-being of the entire population [6, 17]. Equally, this constitutes an important factor in guiding community-based nursing and midwifery approaches. The twin concepts of integration and collaboration are key to providing care to patients/clients with complex needs e.g. chronic diseases, as well as delivering preventative care. Papers specific to integration and collaboration referred mainly to home-based care [18–21], collaborative care [22–25], and case management [26–28], with the majority of these interventions led by community-based nurses including practice nurses, nurse practitioners/advanced nurse practitioners, public health nurses, specialist nurses, and multidisciplinary teams.

Directors of public health nursing (DPHN) revealed a number of initiatives to address the challenges relating to fragmented health services both in Ireland [29] (Nally: Response to Email to Director of Public health Nursing (DPHN), unpublished; Burke: Response to Email to Director of Public health Nursing (DPHN), unpublished) and in other jurisdictions [30, 31] with the aim of achieving better integration and collaboration. Examples include Virtual Wards [30] for the integration of health and social care for people with multiple chronic conditions in the community, and Integrated Care Pilots in the UK, yet efficacy and patient service evaluations were often mixed [31]. High quality evidence demonstrates the positive value of nurse-led interventions as an alternative to physician-led care [20, 26, 32–36]. Martinez-Gonzales et al. [37] in a high quality global systematic review and meta-analysis (*n* = 30,247) found that nurse-led care for chronic disease management compared favourably with physician-led care when nurses were adequately trained to manage complex conditions with collaboration through minor support and short communication with the physician. Substituting nurse specialist-led interventions for physician-led in primary care is recommended in relation to continence management [33] and diabetes management [34]. Furthermore, Nurse Practitioners (NPs) providing complementary or alternative primary care have equivalent or better patient outcomes than physician-led care and are potentially cost saving. Although consultations were longer, patient satisfaction was higher [36] but as evidence is sparse, more research is needed on economic analysis [18, 22, 33, 35, 36]. In addition, midwife-led models of care for low-risk women have been found to be cost effective, safe, require fewer interventions, and result in greater continuity and satisfaction for women [38–40].

To enhance clinical practice, administrators and providers of services need to undertake a needs analysis to determine the priorities for action. In enabling such a process, targets need to be clearly defined and person-centered [27, 32, 37, 41]. Access to specialist in-patient services integrated with appropriate community intervention is seen as the most efficient use of resources [19, 20, 25]. Primary and secondary prevention focused on health and wellbeing could be seen as inexpensive interventions that could be included in primary care by a nurse or other members of the multidisciplinary team [42–45].

### Theme 2: Organisation and delivery of nursing and midwifery care in the community

Organisation and delivery of nursing and midwifery care in the community is framed within the context of prevention (primary, secondary and tertiary), transitional care, and home-based care. Evidence for primary prevention comes mainly from the domain of maternal and child health. With regard to postnatal maternal and infant care, there appears to be benefits to home visits by trained specialist nurses when providing theoretically underpinned interventions with specific person-centred outcomes [46–53]. However, evidence is inconclusive due to the high risk of bias in RCTs. A Cochrane review concluded that valid and reliable outcomes for maternal and infant health, specifically educational interventions, are needed to properly establish these benefits [46]. The interventions had at their core a focus on improving maternal agency and self-efficacy [54].

At the secondary prevention level, community based nursing initiatives were found to be effective in relation to immunisations for children [55], community obesity management [56], and cardiac intervention for female CVD prevention [57]. In terms of preventative mental health care, motivational interviewing by community psychiatric nurses was effective for young adults [58]. Community Psychiatric nurses also had positive psychological outcomes in promoting improvements in functional cognitive ability, mental health scores, depressive symptomology, and self-perceived quality of health in older persons with cognitive decline [59, 60]. This high quality evidence suggests that there are opportunities to further develop nursing roles to promote better health among those with mental health risks. The care of individuals with chronic illnesses was found to empower patients to become ‘agents for change’ [61]. However, it was acknowledged to be costly, requiring investment in systems supporting client self-management at home [62].

At the tertiary level, the evidence supports interventions for asthma self-care and symptom reduction [63, 64] and non-pharmacological intervention for Type 2 (T2) Diabetes Mellitus (DM) in youth [65]. There is some evidence of the benefits of school-based RCTs on smoking cessation [66], and healthy diet [67], but this evidence is of a lower quality. Likewise, there is moderate evidence for leg ulcer prevention and management [67], and initiatives to increase condom use skills [68]. When interventions are provided by well-educated specialist nurses, using theoretically driven person-centered interventions, with specific outcomes, the evidence demonstrates that nurses are effective, efficient and satisfying to clients [69, 70]. Recommendations for such interventions include innovation in treatment methods to improve adherence [71], as well as further research into crisis resolution interventions for older people [72]. Therefore, community nurses have a significant positive role to play in primary, secondary and tertiary prevention, improving the wellbeing of certain at-risk groups and improving public health outcomes.

Transitional care refers to a point of nexus between hospital and home and is a vital part of the journey of older people with a chronic illness to either a healthier or palliative state. The evidence review team were acutely aware of the recently completed systematic review on tackling delayed discharge and (re) admission avoidance in relation to acute hospitals [73] commissioned by the Department of Health (DoH). These reviewers recommend an integrated, personalised, and multi-disciplinary approach and an interconnected, comprehensive system that spans all levels and types of care, following the individual through their care continuum. The evidence solely from a community perspective was relatively sparse. Blair et al. [74] examined 17 RCTs to assess effectiveness of home versus hospital cardiac rehabilitation. Overall, for outcomes where nursing input was separately identified, transitional care interventions appears to be largely positive in relation to reduced readmission rates [62, 74–76], reduced mortality [74], increased patient satisfaction [75, 77] and improving health-related outcomes [74, 78].

Components of interventions associated with positive outcomes appear to be home visiting [62, 74, 76, 77, 79, 80, 81], nurse-led case management or care coordination [61, 76, 78, 82], telehealth [62] and telephone support [74, 78]. The most effective transitional care models were targeted, home-based, and nurse-led in a case-management or coordinated fashion with clear objectives and measurable outcomes. Evidence from a high quality meta-analysis and SRs revealed that transitional care interventions were effective in terms of health-related outcomes, self-efficacy and patient satisfaction in the intermediate and long-term [76, 77]. However, the interventions had to be of high intensity in terms of nursing and other input to achieve short-term outcomes such as reduced re-hospitalisation rates [78, 79]. The effective models needed to have clear and close integration across settings i.e. community to clinic or hospital and could be organised and delivered in either an outreach or community-based way. Furthermore, Coffey et al. [73] advocate for supported communication between primary and secondary care in order to achieve effective transitions.

Home-based nursing is an integral component of the delivery of nursing in the community and the literature revealed it to have a primarily positive influence. Preventive and curative interventions were delivered across the lifespan from birth to old age and most interventions were nurse-led. High quality studies supported home-based primary and secondary care interventions in the context of children and families [58, 60, 83–89] and children with acute, chronic, complex or palliative care needs. Home-based service models should be carefully planned to align local need with adequate integration with existing services, as described by Rand [31]. Consequently, a strong primary health care system, which is both flexible and responsive, is imperative [90].

A large scale high quality Systematic Review [91] found clear and reliable evidence that home-based palliative care increases the chance of dying at home and reduces symptom burden for patients with cancer in particular. Luckett et al. [92] address the organisation of services for persons with life-limiting illnesses, concluding that future trials must compare the relative efficacy of different models and intensities of specialist palliative care services (SPCSs) providing home nursing. In particular, models need to prioritise the person’s preference for the setting of palliative care delivery, and to address the greater economic cost of homecare as opposed to hospital care, as identified in one Australian study [93].

In terms of chronic illness, some consultation programmes by Advanced Nurse Practitoners can be effective in reducing adverse health outcomes (acute events, falls, and hospitalizations) and were also cost effective [94]. In contrast, outcomes associated with leg ulcer interventions reveal neutral evidence with regards to healing rates or proportion of ulcers healed in the home setting [95, 96]. Nevertheless, the Leg Club initiative was deemed to enhance integration and collaboration of care, resulting in improved care and quality of life for patients [61] and was both cost effective and efficient [67]. However, these initiatives are dependent on patients/clients having the mobility and means to access the services.

Nurses and health visitor-led health promotion programmes for older people improved physical functioning linked with activities of daily living, self-care and management, improved self-efficacy and agency, decreased hospitalisation and readmission rates, and increased opportunity to receive care and remain at home [68, 96]. Whether home-based health promotion interventions offer good value for money remains unclear. Yet, a unilateral focus on cost containment alone has been identified by the Canadian Nurses’ Association [97] as limiting community nursing’s scope. Thus the implementation of a model of care must be underpinned by multiple considerations to enable positive outcomes [98].

The precise effective dose of home-based nursing has not been adequately researched. However, the review did conclude that nurse-led care is more cost-effective than medical care [92, 98]. Enabling components were considered to be the educational, supportive and preventative interventions employed [99] which could optimize population health [100, 101]. In particular, community nursing was considered important for vulnerable [102] or marginalized populations [103]. Moreover, home-based nursing facilitates increased opportunities to observe self-management barriers and creates more viable interventions for client’s own self-management [104]. Although standardised approaches to economic evaluation in cost-effectiveness needs further review, the evidence on interventions has implications for the organisation and delivery of community nursing in terms of ensuring the continuance of care delivery in the home setting [92].

### Theme 3 adjuncts to nursing and midwifery in the community

Adjuncts to nursing and midwifery in the community refer to non-licensed personnel and technological supports. Non-licensed personnel are those that work under the direction of a professional - usually a nurse, and who do not have a professional qualification. The interventions relate primarily to maternal and child health [54, 105, 106] and high-risk families with young children [107] where nurse-led interventions with unlicensed personnel had significant positive effects on maternal health. This was particularly related to emotional well-being and depressive symptomology [54, 108, 109]; improved birth outcomes, child physical health [110]; breastfeeding duration and parenting skills [105–108, 110]; attendance, and preventative child health appointments [105]. Nurse-led interventions with supporting non-licensed personnel included nutritional guidance and screening to support risk reduction in chronic disease management for young adults [111, 112].

In studies with adults with cardiovascular disease, nursing in the community with non-licensed personnel resulted in clinical improvements such as reduced cholesterol level and blood pressure [111, 112] and a reduction in the relative risk of Emergency Department (ED) visits [113]. In the provision of homecare to adults and older people, certified nursing assistants are a core element of the nursing teams and well evaluated by patients [69, 70]. However, the higher ratio of registered nurses to non-professional assistants is considered to be the component of higher levels of satisfaction in comparison with other Dutch healthcare models [69, 70]. This model is of significant international interest due to cost savings (as compared to traditional models), client satisfaction and staff satisfaction [70] and is currently being piloted in areas within the UK, America and Japan.

Tele-healthcare interventions in the community are considered an additional resource for nurses and midwives, with promising potential in supporting care. Interventions vary from nurse-led telephone and/or distance monitoring support using algorithm system from patients’ homes, to email, text messaging and electronic sensors with central reporting [114, 115]. With regard to providing access to healthcare professionals in remote/rural areas, tele-visits/video conferencing can provide a forum for patients to access nursing and midwifery care in a timely manner and reduces costs of travelling for all concerned [116, 117]. For example, in rural Australia, specialist cardiovascular nurses provided additional telephone supports which found a reduction in the number of hospitalisations for patients with heart failure [118].

Community nursing care with the use of technology has been shown to be effective and satisfying to patients, and there is a need for interventions to be underpinned by theory, person-centredness and to have specific patient clinical outcomes. Tele-health, such as telephone health mentoring intervention by community health nurses assists found an increases in self-management [62, 119], improved depression symptoms and general health [116, 120] and heart failure [117] for patients with COPD [116, 117]. Technology is being deployed to expand healthcare delivery beyond traditional hospital and clinic boundaries to the home and community setting. Furthermore, tele-health benefits include providing a support structure which empowers patients to actively participate in their care [53, 61, 121, 122] and enables provision of a person-centred approach [53, 121]. Decisions on the usefulness and effectiveness of small single technology (phone reminders) as well as whole “system” centralized monitoring [115, 120, 122] is difficult due to variability in study interventions, populations, outcome measures and training and level of engagement by nurses.

### Theme 4 An overarching model

No single model of community nursing and midwifery emerges from the literature. Both the empirical and grey literature suggest fragmentation and challenges in framing community nursing and midwifery services internationally, thus the lack of a distinct model is not unique to Ireland. Other countries are similarly reviewing the international literature in a quest for the holy grail of a nursing and midwifery model [123]. Debate in the development of models tends to focus on structuralist issues which include a generalist versus specialist approach and geographical versus non-geographical caseloads. For example, the Buurtzorg neighbourhood care model) [69] is primarily focused on district nursing. Similarly, in the UK, the DoH [124] proposed a district nursing model entitled ‘compassion in practice’, underpinned by integration, effectiveness, a quality driven focus, person-centredness, and supported by telehealth, while ensuring that ‘every contact counts’. The service builds on its strong district nursing foundations and continues to have a curative focus but now emphasises the provision of opportunistic public health interventions while promoting self-care of clients.

Unlike Ireland, Canada’s community nursing model is underpinned by nursing theory and integrated with home health and primary health care principles [104]. Even countries that did not necessarily articulate a nursing theoretical model (Netherlands and Australia) did draw on other theories such as those supporting Nurse Family Partnership [87, 125] and Maternal and Child Health Nursing [108]. The evidence from the empirical literature supports the value of theoretical underpinnings, specifying the intervention components which are then matched to appropriate health and social care outcomes. This is very important to match the ambitious goals of policy with the practical ability of the service to meet such goals [97].

Acknowledging this, such a match permits the separation of outcomes at various levels as a means to identify the components necessary to ensure effective, efficient health care outcomes. Despite these benefits, there is a paucity of trials which were theoretically underpinned. This has implications for an Irish model of community nursing and midwifery for the future. Irish models of community nursing [126, 127] have been largely confined to PHN and Community Registered Nurses in the context of primary care teams. Thus they are not universally applicable to the totality of Nursing and Midwifery in the community and there is a danger that they may be discipline-specific rather than a model at a strategic level.

Therefore, a review of nursing and midwifery models requires careful consideration of the totality of community nursing and midwifery development within a lifespan, person-centered approach and systems-based approach, which aligns to parallel health systems in acute care environments. Consequently, this paper identified key competencies for different levels of practice, which were considered in the development of Nursing and Midwifery in the Community Model.

The international literature has similarly examined required competencies for nurses and midwives practicing in a community setting, and this has implications for the development of any model. For example, in the USA, the Council on Linkages between Academia and Public Health Practice (COL) [128] recommended three levels of core competencies for Public Health Professionals. Tier 1 refers to entry level and Tier 3 is at senior managerial level. These have already been adopted in over 50% of state and local health departments and 90% of academic institutions in the USA. Supporting this view in relation to nursing, in Canada there is a requirement for a minimum of two years’ experience with mentoring, leadership and peer support to adequately prepare PHNs for practice [102]. While detail in relation to the expertise of the nurses in the Buurtzorg model [69] is not provided, there is evidence that the competencies required range from generalist to expert depending on the level of specialist care required. From information received from DPHNs, as part of this evidence review, nurses in Ireland in the community are already engaged in continuing professional development education. Some of these include: initiatives to expand scope of practice e.g. chronic disease management, anticoagulation management and ear irrigation. These initiatives demonstrate a community/practice nursing commitment to continuous professional development to enhance service integration and improve the quality of care for clients. In the context of developing a national community nursing and midwifery model, there would need to be standardization in terms of clearly articulating the initial competencies required such as demonstrated in the North American examples above and a clear plan for continuous professional development. This strategic plan would need to be developed in consultation with all stakeholders including the regulatory body Nursing and Midwifery Board of Ireland (NMBI). Implementing this strategic plan requires a national approach supported by effective leadership.

Inherent within any model is the necessity to identify core components which will underpin any effective interventions therein. From this evidence review, those identified are numerous and are frequently context specific. They can be categorised to some extent and include: adopting a care management person-centred approach; led by competent nurses or midwives educated either as specialist or who have received training specific to the interventions; providing targeted home based interventions; supported by tele-health and non-professional support. These components ideally are delivered using a person-centered, team based approach taking cognisance of the determinants of health.

## Discussion

**Fig. 3:**
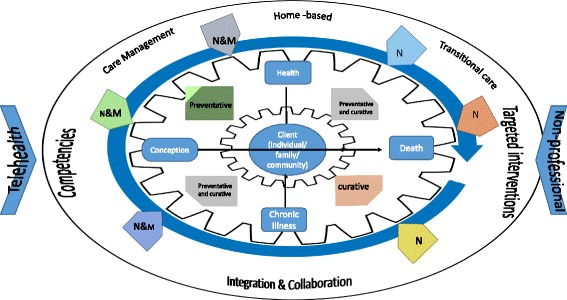
Conceptual Model. Legend N = RGN RPHN RNID RMHN RCN; M = RM

No single overarching model of nursing and midwifery practice in the community emerged from the literature. What did emerge were evidence-based dimensions that can inform an effective model for the future. Figure 3 demonstrates a conceptual framework drawn from the literature which captures the evidence visually. This can be used to inform a lifespan, person-centred approach to providing appropriate and effective nursing and midwifery care in a primary health care context which represents a model for community nursing and midwifery. The key N or M is used in the model purely to differentiate nurse or midwife or other entry points to the register as used by the Nursing and Midwifery Board of Ireland (NMBI). This was utilised to ensure the most relevant registered practitioner (nurse or midwife) is available and appropriately educated to provide care as captured in the model.

This model is underpinned by the principles of primary health care and person-centred care, supported by a philosophy of integration and collaboration. The home based client (individual, family or community) needs to be at the centre and exists on a trajectory from conception to death, and another trajectory from health to chronic illness as demonstrated by the X and Y axes. The client’s health needs on any point of either trajectory can be mainly preventative or curative or a mixture of both as shown by the quadrants. Consequently, client care needs necessitate an appropriately educated, competent, registered nurse or midwife. Depending on the needs of the client, this may entail a generalist or a specialist approach. For example, a client with health needs in the top left quadrant may be a postnatal low risk mother. Therefore, they are on the lower quarter of the conception to death trajectory and the lower quarter of the health to chronic illness. Their needs are health and maternal/child related, therefore the best care to meet their needs is for example a PHN, midwife and practice nurse. In contrast, a client on the bottom right quadrant who is nearer to the end of the lifespan trajectory and the chronic illness or palliative care end of the health trajectory, has a need for a curative focus of care. Curative in this context refers to merely the opposite of preventative. This may be appropriately provided by a generalist nurse supported by specialist nurses such as palliative care or some other specialist related to the nature of the chronic illness. As demonstrated in the model, different size fonts for N and M reflect the level of contribution that may be required at different points. For example a postnatal mother in the bottom left quadrant who had a complicated caesarean birth and whose ill baby is in the neonatal unit, could require nursing and/or midwifery care, depending on client needs.

The dimensions relevant to nursing and midwifery which are critical to effective and efficient practice are illustrated on the perimeter of the image related to: integration and collaboration; transitional care where appropriate; targeted interventions and a care management approach. In fact, transitional care can encompass all of the above and is appropriate across the lifespan and health/illness trajectory. Transitional care interventions are usually nurse led and nurse managed [129] and there is evidence of their effectiveness on patient outcomes [75, 76]. Transitional care however does require close integration across health service settings.

Effective and efficient care is supported by adjuncts including telehealth and non-professionals. In terms of person focused tele-health it could be integrated into chronic care plans especially for younger adults with illnesses such as diabetes, Cystic Fibrosis [78] or other longer term illnesses [116]. Non-professionals, such as community health workers contribute to better child physical health [130] and child and maternal health [108].

The essential components of a proposed model of nursing and midwifery in the community require the right nurse or midwife providing the right care to the right person in the right setting – with transfer of care to and from the most appropriate lead professional at each point of contact. In essence, there is space for both generalist and specialist professionals depending on the care needs of the client, whether at the individual, family or community level. Generalist nursing care supported by specialist nursing care for clients at all levels requires such service input for optimum outcomes. This approach will ensure that clinical outcomes are meaningful, lasting and more sustainable. Operationalising a model for nursing and midwifery in the community demands strong leadership and effective clinical governance, which is seamlessly linked to and interacts with the broader area of health systems.

## Conclusion

This literature documents the fundamental contribution of community-based nursing and midwifery to improving and sustaining population health and well-being. There is a distinct impetus by both individual nations and the WHO that community is the centre of healthcare, and that meeting need at this level requires both responsive nursing and midwifery personnel and the ability of the service to deliver person centred care, which comprehensively meets individualised needs. Nursing and midwifery in the community are complex services and the aim of this evidence review was to identify a model to guide community-based nursing and midwifery in Ireland. Although the empirical and grey literature did not reveal one overarching model, this is not surprising as the care of populations and communities is a complex and diverse phenomenon. Nevertheless, it was possible to identify the components of an appropriate model. The model could lay the foundations for a dynamic and responsive nursing and midwifery service in the community which meets contemporary need but is also adaptable to emerging (and diverse) population need. These components were organized within a framework to inform how a model might be conceptualized, involving either nurses or midwives as leaders or participants in community based health service innovations. These innovations are then implemented in cooperation and integration between nurses or midwives and other community health professionals, and supported by technical and non-technical resources.

No single overarching evidence based design model of nursing and midwifery practice in the community has been described and there has been little attempt at theoretical or conceptual explanation of the complexity of community nursing and midwifery involving multiple services, healthcare professionals, health conditions or physiological events across a life span continuum which require health care and interventions when necessary. In the absence of a theoretical or conceptual framework that explains a model of service provision within the context nursing and midwifery in the community, we have examined the evidence which can inform the development of an optimal model of community nursing and midwifery practice.

## Additional file

Additional file 1: Appendix 1. Data extraction tables. Description of data: Data relates to the studies within each of the six categories as illustrated in Fig. 2. Integrated and Collaborative Care (*n* = 33); Home Based Community Nursing (*n* = 32); Telehealth (*n* = 15); Transitional Care (*n* = 9); Non-Professional (*n* = 10) and Preventative (*n* = 18); which informed the final four themes which is the subject of this paper. (DOCX 172 kb)

## Abbreviations

AMSTAR: A Measurement Tool to Assess systematic Reviews
COL: Council on Linkages between Academia and Public Health Practice
COPD: Chronic Obstructive Pulmonary Disease
CVD: Cardiovascular Disease
DoH: Department of Health
DM: Diabetes Mellitus
ED: Emergency Department
N/A: Not applicable
N&MCM: Nursing & Midwifery in the Community Model
NMBI: Nursing and Midwifery Board of Ireland
NPs: Nurse Practitioners
PHN: Public Health Nurse
PRISMA: Preferred Reporting Items for Systematic Reviews and Meta-Analysis
RCT: Randomised Controlled Trial
SPCSs: Specialist Palliative Care Services
SRs: Systematic Reviews
T2: Type 2
UK: United Kingdom
USA: United States of America
WHO: World Health Organisation
