# Supplementary material for: Conceptualising a model to guide nursing and midwifery in the community guided by an evidence review
Source: BMC Nurs. 2017 Jun 29;16:35. doi: 10.1186/s12912-017-0225-3 (PMC5492933; doi:10.1186/s12912-017-0225-3)
Supplement: Additional file 1: Appendix 1. — Data extraction tables. Description of data: Data relates to the studies within each of the six categories as illustrated in Fig. 2. Integrated and Collaborative Care (n = 33); Home Based Community Nursing (n = 32); Telehealth (n = 15); Transitional Care (n = 9); Non-Professional (n = 10) and Preventative (n = 18); which informed the final four themes which is the subject of this paper. (DOCX 172 kb) [file 12912_2017_225_MOESM1_ESM.docx]

# Appendix 1: Data Extraction Tables

# Integrated and Collaborative care

| **Multidisiplinary** | | | | | |
| --- | --- | --- | --- | --- | --- |
| **Source**  **Country**  **Type of Evidence**  **Aim** | **1. Definitions**  **2. Overview of Models**  **3. Main components** | **4. Population Group and Size**  **5. Health Condition / Problem**  **6. Healthcare context / setting addressed**  **7. Nursing disciplines involved** | **8. Outcomes assessed and effects on outcomes**  **9. Components associated with improved outcomes** | **10. Resource Implications**  **11. Enablers**  **12. Barriers** | **13. Key conclusions**  **14. Recommendations for Practice, Policy, Education, Research** |
| Desiree et al. (2013).  Holland.  RCT  *“To evaluate the effectiveness of collaborative stepped care in the treatment of common mental disorders.”* (P.132). | 1. NR.  2. The collaborative care model incorporates multiple interventions and has been extensively studied in the field of depression.  Three different algorithms, for depression, anxiety and stress related disorders. These were further divided into mild and moderate.  3. This was a two-step intervention with the first step happening in primary care. Five 45 minute sessions on self-help based around psycho education, cognitive and behavioral exercises. Patients with moderate to severe disorders also received medication. Step 2 occurred in the specialist secondary mental health service. | 4. Adults 18+ Years (n=163) .  5. Common mental disorders diagnosed by GP including, panic disorder, agoraphobia, social phobia, specific phobia, generalized anxiety disorder, unipolar major and minor depressive disorder of dysthymia.  6. Step 1 in Primary Care in GP Practice and Step 2 in Out Patient Secondary Mental Health Service.  7. Psychiatric Nurses. | 8. At 4-month post test the intervention group results were superior to the TAU group 74.7% (n=68) v. 50.8% (n=31) responders (P=0.003). At 8 and 12 month post-test no significant differences were found.  9. It is suggested that this approach brings about a faster response in remission rates. The rapid provision of low intensity treatment in primary care and improved collaboration between healthcare providers was deemed to be important. | 10. Training costs for GP’s and Nursing Team.  11. NR.  12. Possibility of selection bias. Difficulty in recruiting GP’s and retaining GP’s after randomization to TAU group. | 13. Replicate study with larger participant numbers with a particular focus on cost-effectiveness.  14. NR. |
| Levine et al. (2012).  USA.  RCT  To test the effectiveness of the Choices for Healthy Aging (CHA) program vs usual primary care for reducing medical service use and improving satisfaction with care among a high-risk group of patients enrolled in a managed care organization located in Southern California. | 1. NR.  2. Choices for Healthy Aging (CHA) program  An interdisciplinary team that provided care in the home for chronically ill patients at high risk for hospitalization  3. (a) Early identification and treatment of exacerbation of the illness, (b) patient-specific health education, (c) self-management or caregiver management of the disease, and (d) advance care planning and other psychosocial issues. Follow-up home visits at least once a month. The home care physician was available to visit 24 hours a day, 7 days a week. | 4. Older adults (n=298).  5. Chronically ill patients at high risk for hospitalization.  6. Home.  7. Care delivered via an interdisciplinary team, with core team members consisting of a physician, nurse practitioner, nurse care manager, and a social worker. | 8. Satisfaction with care; Inpatient/ED Utilization; Admission rate; Predictors of medical services; Cost of medical care.  9. Satisfaction: Intervention group at 6 months reported significantly higher mean satisfaction with care than the usual care group (t = 2.24; P = .026) and significantly greater mean change in overall satisfaction with care compared with the usual care group (10.92 vs 1.93 respectively; t = 3.21; P = .002).  Inpatient: Percentages of participants in the CHA and usual care groups who utilized 1 or more hospital inpatient days in the 12 months following study enrollment were 25.6% and 37.1%, respectively, a significant difference (c2 = 4.56, P = .02).  Hospital Admission rate: The CHA group had a total of 78 hospital admissions for a 12-month admission rate per thousand of 500; the usual care group had 93 hospital admissions and an admission rate per thousand of 664.29. | 10. Costs on average more than $2000 lower than costs of care for those enrolled in usual care. However lower hospital use among the intervention group, did not translate to a corresponding reduction in overall healthcare costs when adjusted for demographics and health conditions.  11. Team of medical and social service providers.  12. The intensity of the intervention may have outweighed the medical need for some of the patients.  The risk stratification statistical program utilized for patient selection identified patients with fewer medical needs. | 13. A program using a home-based interdisciplinary team of medical and social service providers can improve patient satisfaction with healthcare.  14. Additional research is needed to determine better methods to identify high-risk patients efficiently to improve clinical and service outcomes and reduce the cost of care.  Managed care organizations need to consider targeting when designing programs for high-risk groups. |
| Low et al. (2011).  Australia  Systematic Review (n=35 i.e. 18 RCTs, 5 x non RCT and 12 x observational studies)  To evaluate the outcomes of case managed, integrated or consumer directed home and community care services for older persons, including those with dementia. | 1. Case management is defined as collaborative process of assessment, planning, facilitation and advocacy for options and services to meet an individual’s health needs, through communication and coordination of available resources, to promote quality cost effective outcomes (p1).  Integrated care has been defined as a discrete set of techniques and organizational models designed to create connectivity, alignment and collaboration within and between the cure and care sectors at the funding, administrative and/or provider levels (p2).  Consumer directed care is conceptualized as giving consumers greater awareness, control and responsibility for their health care spending (p.2).  2. Case management, integrated care, consumer directed care.  3. Case management: telephone-based case management; computer program assisted case management and case management in combination with cost subsidies. A central worker provided assessment, care planning, coordination of services and ongoing follow-up.  Integrated care e.g. Program of All Inclusive Care for the Elderly (PACE) and Kaiser Permanente Northwest i.e. models where services were formally linked and coordinated.  Consumer Directed: involves a budget for the purchase of services. Consumer choice ranging from selecting the type of services or selecting the service provider to hiring and supervising care staff. | 4. Adults > 60 years (n=53,597).  5. Chronic illness and those with dementia.  6. Home.  7. MTD Team, Nurse case managers, Physicians, case managers : social workers, primary care providers, Geriatric specialists. | 8. Clinical outcomes: increased function, medication management, QOL, Social interaction, physical health.  Decreased: depression, caregiver burden, pain, risk of mortality.  Satisfaction with care  Caregiver satisfaction  Life satisfaction  Service Use: risk of admission to hospital, ER, Nursing home, LOS; Community services use.  9. Case management –evidence of improvements in clinical outcomes.  Integrated care increases service use and does not improve clinical outcomes.  The lowest quality evidence was for consumer directed care which appears to increase satisfaction with care and community service use but has little effect on clinical outcomes. | 10. Did not consider the cost-benefits of different models of community care.  11. Combining key elements of all three models may maximize outcomes.  Key elements: a fully integrated care system which facilitates access to health and community services, in which consumers receive case management to maximize clinical outcomes and prevent unnecessary institutionalization and hospital use, and where consumers have as much control of their own care as they wish.  12. The inconsistencies in results between studies are notable - the studies reviewed were heterogeneous in their inclusion criteria, design, sample and methods of delivery. There was variability in the choice of instruments to measure outcomes, as well as outcomes measured. Most importantly, the health and social care systems in which the evaluations were conducted differ significantly | 13. Different models of home and community care have differing outcomes depending on their focus.  14. Administrators and providers of services need to be explicitly clear as to the focus of their service and prioritization of outcomes.  Future evaluations of community and home care should give detailed descriptors of the service context, intervention and care received by controls, and should measure a broad range of outcomes clinical and service outcomes. |
| Senior et al. (2014).  New Zealand.  RCT.  To evaluate the effectiveness of a restorative care service: Promoting Independence Programmes (PIP) on institutional-free survival and health outcomes in frail older people referred for needs assessment vs usual care in New Zealand. | 1. NR.  2. Promoting Independence Programmes (PIP) Case management restorative care model  3. PIP: Consists of comprehensive geriatric assessment followed by an integrated care plan and rehabilitation delivered by a multi-disciplinary team. combines case management, service coordination and home care with a restorative focus,  Usual Care: community services. | 4. Older People >65 (n=105)  5. Chronic illness and Several IADL & ADL difficulty.  6. At home.  7. Multidisciplinary Teams. | 8. Placement in residential care. Physical health of care giver. Caregiver burden. Use of personal care, home help, carer support, respite, day centre and day activity centres. Functional outcome measures.  9. Rate of decline in physical health for the caregiver in PIP was significantly slower than for usual care (P < 0.01)  Use of personal care, home help, carer support, respite, day centre and day activity centers more than the usual care group- not statistically significant. | 10. NR.  11. NR.  12. NR. | 13. Restorative care reduced permanent residential care placement compared with usual care for frail older people. Case management combined with multi-disciplinary care positively impacts on institutional-free survival for frail older people, but definitive evidence has been lacking  14. Future research required to test this service model as a positive improvement in outcomes, especially given the positive trends demonstrated. |
| Stall et al. (2014).  Canada.  Syst Rev. (n=10 i.e.  Observational study x 8  Cohort x1  RCT (Multisite) x 10  To describe the effect of home-based primary care for homebound older adults on individual, caregiver, and systems outcomes. | 1. NR.  2. Home-based primary care (HBPC). Comprehensive  ongoing primary care in the home..  3. Core program components: Interprofessional care teams,  Regular interprofessional care meetings and after-hours support. Initial in-home comprehensive geriatric assessment. Continuous systematic screening and patient care management. | 4. Older adults > 65  (n =46,154).  5. Mainly veterans from the U.S. Veterans Affairs  System.  6. Home-based primary care  7. Primary care provider /Medical directors; geriatricians; Nurse Practitioner, RN,  Social Workers, pharmacists, dieticians, OT, kinesiotherapists. | 8. Hospitalizations (pre and post) hospital bed days, emergency department visits,  long-term care admissions, and long-term care bed days,  Functional status; individual and caregiver satisfaction.  cost analyses, medical education.  9. Significant reductions  in hospitalizations (23% - 84% (P < .001 – p< 0.01) x 7 studies.  Reductions in inpatient days before  and after the HBPC intervention X 4 Studies: 37.4% (P = .04), 49.9%  (P = .001), 69% and 62% (no P-values reported).  Reduction in long-term care  admissions X 3 Studies (10% (no P-value reported), 20% (P = .001), and 25% (no P-value reported. | 10. Two reported substantial cost savings. Another two reported higher costs per patient after enrolment.  11. Ongoing house visits by  primary care provider.  Comprehensive and ongoing primary care in the home.  12. NR. | 13. HBPC for homebound older adults can positively affect several important individual, caregiver, and system outcome. HBPC demonstrated substantial reductions in at least  one of the inclusion outcomes of emergency department visits, hospitalizations, hospital beds days , long-term care admissions, and long-term care bed days.  14. More-robust financial  analyses are required to definitively determine whether HBPC is cost effective. |

| **Nurse as case manager** | | | | | |
| --- | --- | --- | --- | --- | --- |
| **Source**  **Country**  **Type of Evidence**  **Aim** | **1. Definitions**  **2. Overview of Models**  **3. Main components** | **4. Population Group and Size**  **5. Health Condition / Problem**  **6. Healthcare context / setting addressed**  **7. Nursing disciplines involved** | **8. Outcomes assessed and effects on outcomes**  **9. Components associated with improved outcomes** | **10. Resource Implications**  **11. Enablers**  **12. Barriers** | **13. Key conclusions**  **14. Recommendations for Practice, Policy, Education, Research** |
| Adlbrecht et al. (2011).  Austria.  RCT + cost effectiveness study.  To investigate *“a new disease management programme comparing usual care (UC) to home-based nurse care (HNC)”* and a BNC group (p. 315) | 1. NR.  2. Disease management programme with 3 different models; hospital-based CHF clinic, HNC and the hybrid model BNC.  3. UC = outpatient care by specialist physician  HNC = four home visits  (1, 3, 6 and 12 months after discharge) and telephone contact by specialist HF nurse. Nurse assessed vitals, recorded interpreted needs and coordinated with treating physician to implement guideline-based medication. Nurse in charge of individualized patient and caregiver education and promoting self-management.  BNC = as for HNC group + medical care decision-making based on amino terminal pro B natriuretic peptide levels. | 4. Adult patients >60 years (n=190).  5. Congestive heart failure (CHF).  6. Specialised Outpatients and Home.  7. Specialised HF nurses. | 8. The study found a substantial reduction in costs per year (after 18 mths) by 57% in BNC compared with UC, while HNC was found to be at least cost neutral, taking account of worsening heart failure re-hospitalization rates. Costs per live year saved were decreased by 74% (UC vs. BNC) to €3978 per year (P = 0Æ011). HNC also reduced costs but this did not reach statistical  Significance.  9. Knowing the NT-proBNP blood level allows the treating physician to anticipate worsening CHF and thereby reduce re-hospitalization, but also permits varying the intensity of the home care thus optimizing financial resources. | 10. Costs for the heart failure nurse were calculated based on hospital bills indicating costs of €80 per patient visit. This included expenditures for patient telephone contact.  11. NR.  12. Specialised blood test provided to BNC group supported by study funders. | 13 There are very important benefits to anticipation of worsening CHF in reducing re-hospitalization and costs.  The authors describe this as the first publication where cost-effectiveness for such models of care was calculated.  **14.** The study could assist as a statistical model for future similar cost analyses. |

| Albers-Heitner. (2012).  The Netherlands.  Randomized Controlled Trial + Cost Effectiveness Study  To determine the 12 month societal cost effectiveness of involving urinary incontinence nurse specialists in primary care compared to care as usual by GP’s. | 1. NR.  2. Targeted intervention.  3. Nurse asked patients to complete micturition diaries and advised them on lifestyle toileting habits, bladder and pelvic floor muscle training. | 4. Patients with urinary incontinence (n=350).  5. Urinary incontinence.  6. Primary Care.  7. Urology Clinical Nurse Specialists. | 8. Quality Adjusted and Life Year based on societal preferences for health outcomes. Quality adjusted life year based on patient preferences and the newly developed incontinence severity weighted life year.  9. Both QALY patient and ISLY yield slightly more favorable cost effectiveness results. | 10. NR.  11. NR.  12. NR. | 13. Adopting the nurse specialist intervention in primary care is recommended.  14. Conduct more research through careful monitoring of the effectiveness and costs of the intervention in routine practice. |
| --- | --- | --- | --- | --- | --- |
| Boult et al. (2011).  USA.  Cluster RCT  To measure the effects of ‘guided care’ model vs usual care on multimorbid older patients use of 6 health services. | 1. NR.  2. Guided Care provided by registered nurses trained in guided care model and assigned to primary care teams.  3. Comprehensive geriatric  assessment, evidence-based planning, case management, transitional care, self-  management, and caregiver support. | 4. Adults 65+. (n=850).  5. Several chronic conditions associated with aging e.g. CCF, COPD, and Arthritis.  6. Primary Care.  7. Registered Nurses working in Primary Care who completed a course on Guided Care. | 8 Annual use of health services (hospital admissions, hospital days, nursing facility admissions, ED admission, Primary Care visits, Home Health Care episodes)  9. Significant (29.7%) reduction in home health care by patients receiving guided care compared to usual care (OR, 0.70;95% CI, 0.53-0.93). | 10. NR.  11. The environment in which the intervention of Guided care is implemented affected patient outcomes.    12. NR. | 13. Evidence suggests that  the guided care model reduces annual use of health services across all areas.  14. Further research is necessary to establish which environmental factors were enablers of positive patient outcomes. Also recommended to examine the effect of guided care on medication use. |
| Clarke et al. (2010).  Canada.  Meta-analysis.  To compare the benefits and costs of home based intervention HBI with usual care UC in the context of cardiac rehabilitation. | 1. NR.  2. Usual care was defined as normal health care and/or risk factor management at the time the trial was undertaken without supplementary secondary prevention intervention.  HBI included a community focus incorporating Cardiac rehabilitation which was latterly defined as dedicated secondary prevention programs provided by health professionals in an acute (hospital) or community care provider setting.  3. The trials evaluated paper-based (n = 16), telephone based (n = 12), home-visit (n = 5), or electronic (n = 2) interventions. | 4. 39 studies reporting 36 trials were reviewed. Total number of patients not provided varied on outcome 644 to 2150.  5. Cardiac Rehab.  6. Home.  7. Nurses, usually with specialist cardiac knowledge leading intervention in 11 of the studies reviewed  . | 8. All-cause mortality (n=2150). HBI did not significantly improve mortality.  Cardio-vascular events (n=778 to n=2078)  HBI reduced risk of CV events (excluding stroke, transient ischemic attack, and heart failure) by 9%; but did not reach statistical significance (RR: 0.91, 95% CI: 0.78-1.05)  Quality of life (n=644)  HBI significantly improved QOL [weighted mean difference: 0.23; 95% confidence interval (95% CI): 0.02-0.45],  HBI compared with UC resulted in systolic blood pressure (weighted mean difference: − 4.36mmHg; 95% CI: − 6.50 to − 2.22), smoking cessation (difference in proportion: 14%; 95% CI: 0.02-0.26), total cholesterol (standardized mean difference: − 0.33; 95% CI: − 0.57 to − 0.08), and depression (standardized mean difference: − 0.33; 95% CI: − 0.59 to − 0.07). | 10. Approx. US$300 per patient adjusting for inflation.  11. NR.  12. NR. | 13. HBI for CHD are an effective and relatively low-cost supplement to hospital-based cardiac rehabilitation and should be considered for patients who are stable. . Additionally they may address patient access problems.  14. See Q13. |
| Cicolini et al. (2014).  Italy.  RCT  *“To evaluate whether a nurse-led reminder program through email (NRP-e) may improve the existing primary prevention strategy for the management of the main CVDs risk factors in hypertensive patients”.* (p834) | 1. NR.  2. All participants received usual care and had to attend routine follow-up visits 1, 3 and 6 months after enrollment. Every day, all subjects completed a self-assessment form of the adherence to treatment and educational programme. In addition to usual care, the intervention group also received weekly email alerts and phone calls from the Nurse Care Manager (NCM).  3, Recommendations were taken from current guidelines on healthy lifestyle. | 4. Adults (n=198).  5. Hypertensive (on active treatment for hypertension, or systolic blood pressure >140 mmHg; or diastolic blood pressure >90 mmHg).  6. Primary Care Centre.  7. (NCM) coordinated follow-up visits, recorded baseline and follow-up data using structured forms, and carried out the educational programme. | 8. Compared to baseline, after 6 months BMI, alcohol consumption, cigarette smoking, adherence to therapy hours, systolic and diastolic blood pressure, fasting blood glucose, LDL and total cholesterol, and triglycerides significantly decreased in both groups (all p < 0.01). Fruit intake and physical activity significantly increased, salt consumption did not significantly change. The percentage of subjects with low physical activity, uncontrolled hypertension or glycemia, high LDL and total cholesterol and triglycerides decreased in both groups (all p < 0.01). The mean number of alcohol units and cigarettes decreased, however the prevalence of drinkers and smokers did not vary.  9. At 6 months the intervention group showed a statistically significant greater improvement in BMI, alcohol consumption, cigarette smoking, fruit consumption, physical activity, systolic and diastolic blood pressure, LDL and total cholesterol (all p < 0.05). The prevalence of obesity, low fruit consumption, low physical activity, uncontrolled hypertension, high LDL and total cholesterol decreased much more in the intervention group (all p < 0.01). Fasting blood glucose and triglycerides reductions, compliance with therapy hours, did not show a statistically significant difference between groups. The intervention did not affect salt consumption. | 10. The dosing and timing of any intervention to be implemented beyond the research setting into routine clinical practice must be carefully considered, as nurse workload is typically high and time constrained. Once established, the NRP-e was simple and inexpensive, requiring an average of <20 min per day in addition to normal practice. Coordinating follow-up visits, recording data and carrying out brief educational programmes can be part of ordinary duties, and the only additional actions specifically requested by the intervention were sending email alerts (once a week) and making phone calls to non-responding subjects.  11. Use of read receipts useful to determine who required a follow up phone call.  12. Older subjects may be less competent in email management, reading, and comprehension. There may be socio-economic barriers to the use of such interventions (computer access, language, health literacy). | 13. The NRP-e improved a range of CVD risk factors and deserves further evaluation for the inclusion among existing care management approaches. Nurses play a pivotal role in healthcare promotion, and can encourage appropriate strategies to improve medication adherence and healthy lifestyle behaviors.  14. The provision of information alone may not be sufficient for behavior change, and more tailored and targeted interventions are needed. Further studies with longer follow-up and older and more general samples are required to confirm the present findings. |
| Coburn et al. (2012).  USA.  RCT  To evaluate the effect of the Health Quality Partners (HQP) programme of community based nurse care management on mortality up to 5 years post enrolment. | 1. NR.  2. Community based nurse care management – additional assessments of patients to identify physical, functional, cognitive, psychological , behavioural, social and environmental needs through individualised care plans.  3. 15 item home geriatric assessment at outset. Individualised care plan. Group interventions re weight loss/ weight management; exercise; balance; mobility. Collaboration with patients’ primary care physicians & specialists. | 4. Adults aged >65yr  (n=1736).  5. Medicare patients with one of more of six conditions (CAD, heart failure, diabetes, asthma, hypertension or hyperlipidaemia) deemed moderate to high risk of future health risk.  6. Home.  7. Community based nurses. | 8. Reduced risk of mortality. 25% overall reduction HR 0.75 [95% CI 0.57-1.00, p = 0.047] over 4.2 years of follow up (unadjusted for covariates) ; HR 0.73 (95% CI 0.55-0.98, p- = 0.033) when adjusted for sex, age group, primary diagnosis, perceived health, number of medications, hospital stays in last 6 months, tobacco use.    9. Small group analysis undertaken but unreliable due to small numbers. | 10. No statistically significant difference in medical expeditures or health service utilization between study and control groups but subgroup analysis of the high risk stratum were reported to have 29% fewer hospitalisations and 20% overall expenditure than controls. In a subgroup with heart failure, CHD or COPD and at least one hospitalization in the last year have 39% few hospitalisations, 37% fewer ED visits; and 36% less Medicare expenditures – net saving to Medicare of US$397 per participant per month.  11. NR.  12. NR. | 13. The model of community based nurse care management is associated with reduction in all cause mortality among chronically sick older adults participating fee for service Medicare.  14. Future research needs to allow time and cost to case find and enroll participants. Use of aggregated health data would also improve the recruitment process. |
| Coventry et al. (2015).  UK.  RCT  *“To test the effectiveness of an integrated collaborative*  *care model (ICCM) for people with depression and long term*  *physical conditions”* (p.1) compared with Usual care (UC) | 1. NR.  2. ICCM vs UC.  3. ICCM is a brief psychological therapy delivered by a case manager incorporating Behavioural activation, cognitive restructuring, graded exposure, and lifestyle management approaches for up to 8 face-to-face sessions. Included 2 sessions with a practice nurse at session 2 and 8 to collaborate on physical conditions and care plan.  UC was standard clinical practice provided by general practitioners and practice nurses. | 4. Aged 16+ (n=387).  5. Measured depressive symptoms for at least two  weeks in addition to a long term physical conditions -diabetes or heart disease.  6. Primary care. 36 general practices in the north west of England.  7. Practice nurses. | 8. The primary outcome was reduction in symptoms of depression on self-reported symptom checklist-13 depression scale (SCL-D13)  Significant reduction in anxiety symptoms (generalized anxiety disorder.  Improvement but not significant in relation to global QOL, self-management (health education impact questionnaire), disability  9. The integration with practice nursing was considered to be a component in improvements. | 10. Half day workshop training for practice nurses required.  11. The level of collaboration between psychological wellbeing practitioners and nurses was minimal.  12. NR. | 13. Collaborative care containing brief low intensity psychological therapy delivered in conjunction with practice nurses in primary care can reduce self-reported depression and improve chronic disease self-management in people with mental and physical multi-morbidity.  Treatment effect sizes were modest and were less than the pre-specified effect but were achieved in a natural setting with a deprived population with high levels of physical and mental morbidity.  14. Supportive evidence for integration of psychological and physical care for persons with long-term physical conditions. |
| Heise and van Servellen. (2014).  USA.  Systematic Review  To identify research in which nurses played a role in managing anti-depressant medication adherence and to identify the specific nursing components of these interventions which produced improved outcomes. | 1. NR.  2. No single model across studies but case management was the commonest model in the review. Nurse case managers or nurse practitioners working as sole or joint providers of adherence enhancement interventions (e.g. as part of a team).  3. Key components – care managers; nurses in primary care settings; nurse practitioners. | 4. Patients identified as depressed using a standardised measure of depression and as non adherent to their anti-depressant medication using a clear measure of non-adherence.  5. Depression.  6. All included studies US based.  7. Nurse case managers; online trained psychiatric nurses; advanced nurse practitioners (psychiatric nurses with prescriptive authority). | 8 Outcomes generally positive with nurse intervention associated with improved medication adherence. One study showed no significant difference. Size effect data cited for only one study which showed and odd ratio predicting higher treatment adherence in the intervention group of 2.11 (95% CI 1.02-4.36, p=0.04)  9. Care management/ monitoring; education about medicines and depression; feedback to healthcare providers; referral to mental health and social care providers. | 10. NR.  11. NR.  12. NR. | 13. Tentative conclusion that treatment programmes using nurses as case/care managers are significantly better than other approaches.  14. Further research recommended to replicate findings and to separate roles of nurses more adequately. |
| Jonkers et al. (2012).  The Netherlands.  RCT.  To determine whether minimal psychological intervention (MPI) directly or indirectly improves self-efficacy, anxiety, daily functioning and social participation. | 1. NR.  2. Minimal psychological intervention. Five phase psychological intervention by nurse utilizing a diary kept by the patient Model based on CBT and self management  3. Up to a max of 10 visits by nurse over 3 months. Phase 1 – gaining an understanding of origin of symptoms; Phase 2 – diary kept by patient; Phase 3 – diary discussed with nurse and used to help patient link mood and thinking to their behavior; Phase 4 – introduction to self management approach; Phase 5 evaluation of extent to which goals have been met. | 4. Patients aged >60.  5 Minor or mild to moderate depression and type 2 DM or COPD.  6. 89 primary care settings in southern Netherlands.  7. Research nurses trained in DELTA (Depression in the Elderly with Long-Term Afflictions) intervention. | 8. Outcomes – reduced anxiety in intervention group (mean difference 2.5; 95% CI 0.7-4.2); better self efficacy skills (mean difference 1.8, 95% CI 3.4-0.2); better daily functioning (mean difference 1.7; 95% CI 0.6-2.7); better social participation (mean difference 1.3, 95% CI 0.4-2.2) No differences noted between patients with different conditions.  9. NR. | 10. NR.  11. NR.  12. NR. | 13 MPI administered by nurses was reasonably effective in improve care for chronically ill elderly people with minor or mild to moderate depression.  14. Recommends further research to improve the efficacy of MPI. Detection and awareness of depressive symptoms, cognition and behaviours should become an integral part of the treatment of chronic disease and embedded in the regular ask of the practice nurse who monitors the patient’s chronic disease. |
| Kneipp et al. (2011/2013)  USA  A Randomized Controlled Trial  Evaluated the effectiveness of ‘a community-based participatory research–grounded intervention among women receiving Temporary Assistance for Needy Families (TANF) with chronic health conditions in increasing (1) health care visits, (2) Medicaid knowledge and skills, and (3) health and functional status’ (pg 1759)  1) genuine partnerships with the community, (2) shared decision-making, (3) capacity building, and (4) benefiting all partners. | 1. NR.  2. Community-based participatory research–grounded intervention  ‘1) genuine partnerships with the community, (2) shared decision-making, (3) capacity building, and (4) benefiting all partners’ (pg 1760)  3*.* Intervention  9 months of case management delivered in a WTP by a PHN. The case-management interventions attempted to reflected the Stages of Change approach  PHNs interaction were a minimum of 4, 1-hour meetings with intervention group either in office or home visits  Control group  usual care in the local WTP. | 4. Women (n= 432)  receiving assistance from TANF not yet employed,  ages of 18 and 60 years  1 chronic health condition,  5. 1 chronic health condition defined as ‘‘conditions that are generally not cured, once acquired’’ for this study  The condition had to have a reasonable potential for interfering with functional status and, by extension, employment performance and absenteeism.  6. Local WTP, the African American community 1 urban and 1 rural county in north-central Florida  7. PHNs. | 8. Data collected at baseline, 3 months, 6 months, and 9 months  Outcomes Measures : visits to primary care assessed knowledge of Medicaid benefits with a 20-item questionnaire possible range of scores was 0 to 20 Medicaid skills competence using a rating tool that assessed the degree to which participants’ behaviors indicated effective coping skills during standardized role play in 5 dimensions confidence to engage, emotional responses, content accuracy of responses, appropriate questions?,. range of 0 to 15 points, with a higher score indicating greater skills competence  Depression (the BDI-II),  SF-12 version calculated intervention ‘‘nurse dose’’ by having PHNs document the number of minutes spent with intervention group participants.  9. Despite slight improvement in general health, there was no group differences(P= .72).  Functional status had overall improving trends also but non significant between groups (P= .09). | 10. NR.  11. Providing services on-site in local WTP offices, using lay community personnel to deliver Medicaid training, and basing the intervention largely on PHN competencies are features that facilitate transfer into practice  12. Uptake of this intervention requires careful consideration –including, culturally sensitive, acceptable screening tool; PHNs with similar educational levels, experience, and training; and integrating community members as program personnel  partnering with nurse administrators at local health departments. | 13. PHN case-management intervention combined with Medicaid training is effective in improving health care visit rates for mental health, reducing depressive symptoms, and improving functional status among women in a WTP. Medicaid knowledge and skills competence increases were relatively small given the potential range of scores  14. NR. |
| Martin-Misener et al. (2015).  Canada.  Systematic review of 11 RCTs years 1980-2013.  Evaluated nurse practitioners (NP) in alternative and complementary ambulatory care roles and reported health system outcomes to determine the cost effectiveness of NPs delivering primary care and specialized ambulatory care. | 1. Ambulatory care; defined as health services not requiring overnight hospital stay  2. Alternative role; complementary role models for nurse practitioners.  Alternative role: provide similar services to those for who they are substituting, usually physicians; goal is to reduce cost or workload or address workforce shortages  Complementary role: provide complementary or extend existing services; goal is to improve quality of care p2  3. Not defined in adequate detail. | 4. All ages, majority adults >18 (n=7600).  5. All conditions inclusive of CV, DM, hypertension,hi users of services, allergic rx, atopic dermatitis.  6. Ambulatory care in a community or hospital base. 9 studies except are general or primary care practices, OP clinic dermatology, 1 ED, 1 endoscopy clinic.  7. Nurse practitioners. | 8. Clinical outcomes: 4 studies NP care at least equivalent to GP care in pt health outcomes.  Drop diastolic blood pressure at 6 months larger in NP gp (356 patients) (mean difference: −3.0 mm Hg (95% CI−5.54 to −0.46); p=0.04).  NP care higher patient satisfaction (1515 patients; I^2^=0%) (mean difference: 0.15 (95% CI 0.11 to 0.20); p<0.0001) and also parent satisfaction.  9. NP longer consultation times and patients who consulted NP told the cause of their illness (relative risk (RR) 1.12;95% CI 1.06 to 1.19; p=0.0001) (HQE), how to relieve their symptoms (RR 1.27; 95% CI 1.19 to 1.34; p<0.00001) (HQE), and what to do if the problem persisted (RR 1.06; 95% CI 1.02 to 1.09; p=0.002). | 10. Difficult to determine cost effectiveness d/t secondary cost r/t cost of GP practice, other costs.  -Meta-analysis 2 studies (2689 patients) NP care lower mean health services costs per consultation (mean difference:  −€6.41; 95% CI −€9.28 to −€3.55; p<0.0001)  11. NR.  12. NR. | 13. NPs in alternative provider ambulatory primary care roles have equivalent or better patient outcomes than comparators and are potentially cost-saving.  Evidence for NP cost effectiveness in alternative provider specialised ambulatory care roles is promising, but limited by insufficient studies.  14. While some evidence indicates nurse practitioners in complementary provider specialised ambulatory care roles improve patient outcomes, their cost-effectiveness requires further study. |
| Martinez-Gonzales et al. (2014).  Switzerland.    Systematic review & meta-analysis of RCTs (n=11)  *~~“~~to compare effectiveness of nurse-led and physician-lead care on clinical parameters*” p 2. | 1. NR.  2. Nurse-led primary care interventions to manage patient in general practices, community or ambulatory settings.  3. Nurse provided and led care for complex conditions  that required specialized skills . 82% of trails (9/11) = specific guideline or protocol based.  3. In 1 of 11 trials: Nurse full clinical autonomy to manage pts disease. In 10 of 11 trails: nurses made independent judgments (adopting, initiating and prescribing treatment} with minor support or short communication with physician e.g. discuss patient records, develop actions plans, sign prescriptions. | 4. Adults (n=30,247).  5. All conditions including complex conditions: HIV, hypertension, heart failure, CV diseases, DM, asthma, Parkinson’s, incontinence, mental health and addiction.  6. General practices, community, or ambulatory care setting..  7. Nurses – level of training varied. | 8. Clinical outcomes:  a. Systolic BP – significant reducing effect of nurse-led interventions (weighted mean differences WMD -4.27, 95% CI -6.31 to -2.23, p<0.0001) – from 5 trials  b. Cholesterol and triglycerides – no sign diff nurse-led care and physician –led care in reducing mean level chol(TC) (WMD -0.08, 95% -0.22 to 0.07, p = 0.29 – from 4 trails  c. Glycosylated haemoglobin concentration; no sign diff nurse-led and physician-led (WMD 0.12, 95% CCI -0.13 to 0.37, p=0.33  d. Lung and kidney function – so sign differences between nurse- and physician-led care (lung function – peak flow - at 12 or 24 months) or levels urine sodium excretion and serum creatinine at 6 months)  e. Cardiac function: more pts with nurse-led care who had “decrease or regression in levels of functional exercise capacity, N terminal pro-brain natriuretic peptide or left ventricular end-diastolic volume index.    f. Incontinence – no sign difference between nurse and physician-led care – freq(# and volume) or volume (# of pads) of incontinent episodes at 6 or 12 months.  g. Parkinson’s – no sign difference nurse- or physician-led care in fractures during the study or mobility stand-up test at 24 months.  h. HIV – one trial – CD4 cell counts used indicator of ART initiation and …nurse –led care significantly lower CD4 cell counts (p 8) WMD 20, 95% CI 9.29 to 30.71, p=.000.  9. Not explicit/inconclusive & suggested that the level of skills may be critical for the success of disease management when physician nurse substitution takes place.” | 10. NR.  11. NR.  12. NR. | 13. Nurse-led care (delivered by nurse practitioners) demonstrate stat significant systolic BP reducing effect compared to physician-led care ; no sign differences in reducing diastolic BP, total CHOL and glycosylated haemoglobin. Results from the other 32 individual trial estimates reported 9 of the trials suggest nurse-led care) may be similarly (26 estimates) or more (6 estimates) effective than physicians in managing the variety of clinical parameters evaluated” in this analysis.  14. More high quality trials with larger number of patients need to be carried out to inform nurse-led care and comparison to better clinical outcomes. Additional studies should map clinicians’ characteristics, including wider range of nurse care and tasks provided in many countries and various levels of training and clinical autonomy.  Nurses role and level of experience required to qualify for substitution need a better definition of boundaries and task allocation in clinical practice. |
| Martinez-Gonzalez, et al. (2015).  Switzerland  Systematic review of 12 RCTs  To compare ”the evidence about physician-nurse *task shifting* in primary care in relation to course [measures] of the disease and nurses’ roles” (p 1)  Compared family MD, paediatricians and/or geriatricians to nurses (NP, licensed nurse) on outcome measures related to the course of disease –symptoms, severity and complications. P3 | 1. Task shifting =”clearly delineated tasks or functions traditionally from the domain of physicians transferred to nurses” p 3 task/functions “would be delivered with autonomous or delegated responsibility” p 3 (differs from supplementation =nurse complements work of physician or extend range of services).  2 “*Task shifting* carried out in general practices, nurse clinics and healthcare centres, for wide range of possible diagnoses (diverse, minor acute, common or specific), in pts requiring single contact care, single contact and urgent care and /or ongoing care.” P 4  3.”tasks varied widely from assessment, hx taking,preparation, diagnostic, monitoring and prescription to decision on eligibility for and initiation of tx, referral, follow up and secondary prevention” p 4  Use of structured disease-specific protocols combining nonpharma interventions with pharma therapy and use of validated tools used 75-84% of studies p11  Note – Specific nurse tasks are listed within disease reviewed - wide variability. LIMITED DETAIL ON THE EXACT NURSE INTERVENTION(S) COMPONENTS ACROSS all TRIALS | 4. All ages,primarily adults, (n=22,617)  5. Patients with heart, lung, metabolic, digestive, skin, infectious diseases and diverse acute minor or common complaints (8 categories of conditions)  6. General practices, nurse clinics and healthcare centres  7 Nurse practitioners (w/ and w/o additional degree courses, registered nurse, licensed nurses | 8.  Nurse-led care statistically not significantly different from physician-led care in 84% of pt outcomes reported. Remaining 16% statistically favored nurse-led care. From 1-4 trials were used to retrieve like outcome data r/t specific conditions ---  Type of nurse: NP NP+ nurse practitioner, LN licensed nurse, RN registered nurse  (NS= non significant)  **Clinical outcomes**  -Heart disease 12-48 mo NS difference pts w/ chest pain. At 12 months **NP** nurse led care significantly fewer pts (7.1% to 10.8%) who reported worsening chest pains. (RR 0.66, 95%CI 0.44 -0.98)  -lung disease NS difference (**LN**) nurse to physician led care  -metabolic disease @6 mo **NP+** nurse-led showed lower stroke risk (WMD -2.53, 95%CI, -4.32 to -0.74, CHD risk (WMD-2.00 95%Ci -3.14 to -0.86  -digestive disease **NP+** nurse led care 6 mo statistically improved (maintained/reduced) dyspepsia over physician care (WMD -2.30 95%CI -3.19 to -1.41  -Skin disease NS difference **NP+** nurse to physician care outcomes  -infectious disease - NS difference **LN**  nurse to physician care outcomes  -diverse,acute, minor or common complaints **NP+** nurse and **NP** nurses NS difference between nurse and physician care outcomes  **Service outcomes:**  -length of consultations: longer time length by nurses than physicians WMD 1.90-3.80 95%CI 1.32 to 4.26  9. Care provided by **NP** nurse practitioners (p 11);use of clinical guidelines and validated tools – ex protocols combining non-pharma and pharma interventions p 11; suggest interventions that include “provision of info about causes of illness and pts disease” motivates pt to better outcomes; good communication skills by nurses p. 11 | 10. Trained nurses NP and NP+ can address need for healthcare providers to many pt disease in primary care at time of physician anticipated shortage, and cost of nurses less than physician in training and their delivery of healthcare (p 10)  11. Structured protocols, validated tools “might boost outcome improvement.”  “Non pharma and pt centred care approaches may also lead to successful nurse-led care interventions” p 12  “in all studies, nurses were trained and/or took courses for delivering the studies’ interventions.” P 11  12 NR | 13.  -description of nurses’ competencies and training components often lacked detail p 4  -“trained nurses, mostly NPs, appeared to achieve outcomes of at least similar effects as physicians for management of disease progression in a wide range of pt populations.” P 12  -“structured protocols and validated tools might be some of main boosters of outcomes improvement “ in nurse-led care p 12  -“implementation of non pharma and pt centered care approaches may also lead to successful nurse-led care interventions.” P 12  14. Future studies should include clear def of roles, qualifications, skills and experience of nurses (p 12)  Need studies that describe interventions in greater detail ( Note: au does not stipulate whether they refer to nurse or physician led intervention – it is assumed by aim of the paper they mean nurse led intervention ). |
| Ortego et al. (2014).  Spain.  RCT  To assess whether an exercise program supervised in primary care increases functional capacity more than unsupervised walking with incident cases of low-risk acute coronary patients, <80 years old. | 1. NR.  2. Study involved primary care research nurse acting in a supervisory capacity for intervention exercise cycle program. Study suggests based on findings primary care nurses could act in capacity of supervisor of this intervention.  3. Patients were randomly assigned to either the intervention (SE) Group or a Control Group (UW). Patient in SE participated in a 6-month cycle ergometer exercise program supervised by primary care nurses. Those in the control group were assigned to an unsupervised walking program. | 4. Adults <80 years old. (n=97)  5. Low risk Coronary Heart Disease.  6. Primary Care.  7. Primary Care Nurses working in Primary Care Centres. | 8. Functional capacity as measured by peak oxygen consumption (VO2peak) in ml/kg per minute. Blood lipids (total cholesterol, HDL cholesterol, LDL cholesterol, and triglycerides), resting blood pressure, resting heart rate and body weight.  9. VO2 peak increased significantly in the SE group without (5.56 ml/kg per minute; 95% CI 3.38–7.74) and with (3.47 ml/kg per minute; 95% CI 1.76–5.18) values carried forward.  The SE group lost more weight, with a multivariate-adjusted difference of 4.56 kg (95% CI 0.91–8.21; p < 0.05) and 3.60 kg (95% CI 0.78–6.42; p < 0.05), respectively, and had increased their diastolic blood pressure by the end of the study, 4.46 mmHg (95% CI 0.17–8.76; p < 0.05) and 4.06 mmHg (95% CI 0.59–7.51; p < 0.05), compared to the UW group. | 10. Limited resource needs including the use of inexpensive devices such as a cycle ergometer and HR monitor. The facilitation of supervision of this regime could be done by a primary care nurse or other member of the multidisciplinary team such as physical therapist, exercise specialist.  11. The supervision of exercise allowed for patients to be trained with careful control of the amount and intensity of exercise needed to achieve the optimal effect on their cardiorespiratory fitness, while preventing injuries and any adverse effects of exercise.  12. NR. | 13. The SE exercise training program increased functional capacity of low-risk CHD patients by 8% and reduced body weight by 4.5% compared to UW program.  14. This exercise program is an inexpensive intervention that could be implemented in primary care settings supervised by a primary care nurse or other member of the multidisciplinary team. |
| Selvaraj et al. (2012).  Malaysia.  RCT.  To compare COACH (Counselling and Advisory Care for Health) intervention delivered by PCP only, versus PCP assisted by nurse educators PCP-NE. | 1. NR.  2. Patient education and empowerment with decision support as interventional strategies.  3. PCP is an addition to usual care delivered by Primary Care Physicians where all subjects received a COACH health booklet completed during clinic visits.  The PCP-NE COACH programme was the standard care above plus bi-weekly telephone follow-up by trained nurse educators (NE) for 24 weeks incorporating provision of patient’s self-management support, empowerment and discussion guided by the health education booklet. During the telephone follow- up NE provided reinforcement of the health education information and reminded patients to adhere to counselling advice and prescribed medications, as well as discussion of any adherence problems with PCPs. Phone call follow up. | 4. Adults > 18years (n=297).  5. Patients at risk of Coronary Heart Disease (CHD) i.e. with a primary diagnosis of dyslipidaemia.  6. Malaysian primary care practices (n=21).  7. Trained nurse educators | 8. 36-week study and the primary efficacy outcome was the mean percentage change from baseline LDL-C at week 24 between the 2 study arms. Both study arms demonstrated improvement in LDL-C from baseline. However the difference in mean change between groups was 2.55% - not significant (p=0.288), with a greater change seen in the PCP-NE arm.  There was a significant difference in percentage change from baseline of HDL-C between the PCP-NE and PCP groups, 3.01%, 95% CI 0.12-5.90, p=0.041, at week 24 there was no significant difference in lipid outcomes between 2 study groups at week 36 (12 weeks after the programme had ended). There were no significant differences between arms in relation to statin treatment adherence, blood pressure and Framingham Coronary Heart Disease risk scores at week 36. Approx. 90% of subjects from the intervention arm expressed satisfaction with PCP-NE in helping them achieve health care goals through telephone follow-up.  9. The COACH programme delivered more comprehensive care for chronic disease for both arms of the study than usual care. As the health booklet was distributed to all subjects this diluted the difference in interventional effects between control group and intervention group. | 10. Cost of training nurses not described.  11. NR.  12. The lack of statistical significance was considered attributable to dilution of treatment effects with use of similar patient education methods in both groups, specifically, using the COACH health booklet. | 13. Patients who received coaching and advice from PCP with or without NE showed improvement in LDL-cholesterol. Disease management services delivered by PCP-NE showed a trend towards add-on improvements in cholesterol control compared to care delivered by PCP alone. However, these improvements were not maintained on completion.  14. The COACH programme delivered more comprehensive care than usual, compared to the typical Malaysian primary care settings constrained by time and resources. As a result, in this study, patients from both study arms benefited from increased knowledge of their health conditions. Authors concluded that the task of personalised patient education does not have to be solely the domain of the PCPs but can also be delivered by trained nurses.  More significant findings might be elicited with the addition of a third study arm assessing the lipid outcomes among patients who received “actual” standard care delivery by local PCPs. |
| Stewart et al (2014A).  Australia.  RCT  This study aims to assess Standard versus atrial fibrillation- specific management strategy (SAFETY)—a strategy that is specific to atrial fibrillation to reduce recurrent admission and prolong survival. | 1. NR.  2. Nurse-led,home based programme focused on management of atrial fibrillation.  3. SAFETY intervention comprised a home visit and Holter monitoring 7–14 days after discharge by a cardiac nurse with prolonged follow-up and multidisciplinary support as needed. | 4. Patients (n=335) with chronic, non-valvular atrial fibrillation (but not heart failure).  5. Chronic, non-valvular atrial fibrillation.  6. Primary care and hospital outpatient follow-up.  7. Community cardiac nurse, and multidisciplinary team (community). | 8. Event-free survival from unplanned admission or death (both all-cause) and associated days alive and out of hospital.  SAFETY intervention group achieved 146 967 (92%) of a possible 159 133 days alive and out of hospital, compared with those assigned standard management, who achieved 141 113 (89%) of a possible 158 446 days alive and out of hospital. Effect size  0.39, 95% CI 0.38–0.41; p=0250.  Median event-free survival as a proportion of maximum versus actual days alive and out of hospital (the coprimary endpoint) was prolonged significantly in the SAFETY intervention group compared with the standard managment group (99.5%, 95% CI 99.3–99.7 vs 99.2%, 98.8–99.4; effect size 0.22, 95% CI 0.21–0.23; p=0.039).  9. NR. | 10. NR.  11. NR.  12. NR. | 13. The atrial fibrillation-specific management strategy described in this trial represents a feasible and potentially cost-effective means to improve health outcomes for an increasing number of older individuals presenting with chronic atrial fibrillation and complex comorbidity, in whom recurrent admissions and premature mortality is common.  14. A specialist outpatient clinic could be established to provide rapid and definitive assessment and management of patients with chronic atrial fibrillation. |
| Swan et al. (2015).  USA.  Systematic Review (7 RCT’s, 2 economic evaluations, 1 two year follow up study of included RCTs)  To evaluate the *“safety and effectiveness of primary care provided by advanced practice nurses (APNs) and evaluate the potential of their deployment to help alleviate primary care shortages”.* (p, 2) | 1. NR.  2. Exploring the use of non-physician providers in primary care i.e. nurse practitioners (NPs), advanced practice nurses (APNs), physician assistant and midwives.  3. NR. | 4. Patients, Nurse Practitioners and Advanced Nurse Practitioners (n= 10,911).  5. Cost and quality of care provided by APNs in primary care.  6.Primary Care.  7. Nurse Practitioners and Advanced Nurse Practitioners. | 8. Physiological measure; Patient satisfaction; Cost of care; Healthcare resource; Process measures  9. Physiological- Cholesterol/high-density lipoprotein (HDL) ratio / diastolic blood pressure at 6 months were more favorable in ANP groups.  Patient satisfaction- Four studies identified greater patient satisfaction in patients who received their care from ANPs and one study reported higher satisfaction  Cost- Three studies estimated that APN care was given at a reduced cost compared with physicians using provider salary.  Health Resources- Four studies explored consultation length: three identified APN were 3.0 to 4.3 minutes longer than physician consultations; Two RCTs reported fewer primary care visits among APN patients at 2 years; Patients often requested a return visit with ANPs and were more inclined to keep the appointment.  Process Measures-One study identified APNs had providing increased rates of disease-appropriate care; patients assigned to the physician group versus ANP groups were less likely to have been told the cause of their illness (odds ratio [OR] 0.58, 95% confidence interval [CI] 0.44–0.76); how to relieve symptoms (OR 0.32, 95% CI 0.24–0.43) and what to do if the problem persisted (OR 0.61, 95% CI 0.41–0.90). | 10. APNs delivered different care than that of a physician. However, quality remained the same with equal and/or lower cost.  11. There is a need to remove barriers that impede APNs from practicing to the full of their training potential.  12. NR. | 13. APNs in primary care have accomplished equally as well as physicians in relation to clinical outcomes and patient satisfaction.  14. Future studies need to identify further outcomes that were omitted in this review. Furthermore, studies should include incidence of preventable hospitalisations; preventive care i.e. vaccines and disease screening. Future studies should include longer follow-up periods this will allow for the assessment of rates of retention in care |
| **GP/Physician as Case Manager** | | | | | |
| Katon et al. (2012).  USA.  Cost effectiveness + RCT.  To evaluate the cost-effectiveness TEAMcare  compared with usual primary care (UC) in outpatients  with depression and poorly controlled diabetes or CHD. | 1. NR.  2. TEAMcare is a multi-condition collaborative treatment program composed of Physician-supervised nurse care to identify clinical goals and to develop individualized care plans.  3. Nurses educated patients and used motivational  interviewing, behavioral activation and problem-solving strategies to help patients undertake specific self-care activities. Most participants had at least 4 visits and 3-4 telephone contacts per month from the nurse.  UC participants consulted their primary care provided for concerns relating to depression CHD or diabetes | 4. Adults (n=214).  5. With depressive disorder and poorly controlled CHD or diabetes.  6. 14 primary care settings.  7. Practice nurses. | 8 Cost as measured by QALY, Depression scores and Hemoglobin A1c (HbA1c), systolic blood pressure (SBP), Low-density lipoprotein cholesterol (LDL-C) levels.  Total intervention cost per patient was $1224.  Over 24 months, intervention patients had a mean of 114 (95% CI, 79 to149) additional depression-free days and an estimated 0.335 (95% CI, −0.18 to 0.85) additional QALYs compared with UC controls.  Intervention patients also had lower mean outpatient health costs of $594 per patient (95% CI, −$3241 to $2053) relative to UC patients.  For adults with depression and poorly controlled diabetes, CHD, or both, the intervention seemed to be a high-value program that markedly improved QALYs -  mean cost saving of $1773 per QALY.  9. NR. | 10. See Q8.  11. Intervention can be readily implemented.  12. NR. | 13. This nurse delivered intervention has important benefits in terms of cost effectiveness and increase in depression-free days.  14. NR. |
| Richardson et al. (2013).  UK.  Cost effectiveness + RCT  To present a cost effectiveness analysis, comparing costs and Health Related Quality of Life (HRQoL), as measured by quality-adjusted life years (QALYs), associated with Supporting Listening (SL), Pragmatic Rehabilitation (PR) and Treatment as Usual (TAU) for people living with Chronic Fatigue Syndrome /Myalgic Encephalitis (CFS/ME). | 1. NR.  2. SL A non-directive counseling approach.  PR involves nurses explaining symptoms in the context of a model of illness, providing a rationale for a collaboratively developed rehab programme, presented verbally and with a manual.  TAU – provided by GP.  3. PR included an individualized programme of activity and improved sleep hygiene. PR and SL delivered over 10 sessions. | 4. Adults 18+ years (n=296).  5. CFS/ME for longer than 6 months.  6. Primary Care.  7. Adult Speciality General Nurses working in Primary Care with no prior experience of CFS/ME. | 8. Costs (Private expenditures, costs of informal care and costs of lost production at 08/09 prices to NHS) and outcomes as measured by QALYs. HRQL increased in all groups at Wk. 20. Returned toward baseline at Wk.70.  9. TAU delivered in primary care slightly more effective than PR or SL and at a lower cost. | 10. NR.  11. Patients need to believe in the intervention model presented in order to engage fully.  12. NR. | 13. Evidence suggests that patients with CFS/ME are not satisfied with the care received from primary care, and GPs find management difficult. Suggesting cost-effective solutions should be implemented is of limited use if patients feel that this option is not acceptable.  14. Research into effective management options for patients with CFS/ME required. In addition, the skills of GPs in diagnosing CFS/ME and offering acceptable treatment, or referral to specialist CFS/ME services, needs improvement. |
| **Practice Nursing** | | | | | |
| Clark et al (2012).  UK.  RCT.  To determine whether screening for osteoporosis led to increased prescribing of medication for osteoporosis and reduced the incidence of new fracture. | 1. NR.  2. Administration of a four item screening tool for osteoporosis.  3. 20 minute screening procedure including height measurement, back pain score, history of previous fracture and rib-pelvis measurement. Subjects scoring below threshold (4) evaluated by plain radiograph. | 4. Women aged 65-80 years (n= 3,200).  5. Population screening study.  6. Recruited without specific exclusion criteria from 15 general practices in South West England.  7. Research nurses trained to the standard of practice nurses. | 8. Allocation to screening increased prescription of osteoporosis medications by 124% (odds ratio [OR] for prescription 2.24 at 6 months; 95% confidence interval [CI], 1.16 to 4.33) and reduced fracture incidence at 12-month follow-up (OR for new fracture 0.60; 95% CI, 0.35–1.03; p.0.063) - although this did not reach statistical significance.  9. Screening alone associated with improved outcome although radiographic confirmation of previous vertebral fracture more strongly associated. | 10. Preliminary cost effectiveness analysis suggest cost per QALY of £3000 although could be less if more women found to have fracture were prescribed medication.  11. NR.  12. NR. | 13. Use of a screening tool for osteoporosis in addition to an information leaflet is worthwhile and the tool in this study may be better than alternative risk assessment tools.  14. More education of GPs may be required to improve prescribing rates in women identified by screening and maximize benefit. |
| Elley et al. (2011).  New Zealand.  RCT.  *“To assess the cost-effectiveness of exercise on prescription with ongoing support in general practice”* (p.1223) | 1. NR.  2. The original Green Prescription intervention involved brief advice and an exercise prescription  usually delivered by the  GP, followed by monthly telephone support for 3  months delivered by regional sports trusts.  3. Ten mins of brief advice; written exercise prescription given by a primary healthcare nurse; telephone support for 9 months from an exercise facilitator; half-hour face-to-face session with the nurse at 6 months. Goal was at least 30 min of moderate-intensity physical activity five times per week. | 4. Women aged 40–74 (n=1089)  5. Women who were less active than those performing moderate intensity exercise.  6. General Practice  7. Primary care nurses | 8. Primary outcome – incremental cost of moving a person into an “active” category. Secondary outcome- incremental cost of increasing physical activity at 12 and 24 months.  9. Significant improvements in physical activity were found at 12 and 24 months (p<0.01); no significant difference in indirect costs between both groups (12 months: rate ratios: 0.99(95% CI 0.81 to 1.2) and ( 24months: rate ratio: 1.01 (95% CI 0.83 to 1.23, p=0.9); Cost–effectiveness ratios using programme costs were NZ$687 (€331) per person made ‘active’ and sustained - 12 months; NZ$1407 (€678) per person made ‘active’ and sustained - 24 months. | 10. The total cost of the  Green Prescription programme was  $93.68 per participant.  11. Extra telephone support;  6-month face-to-face nurse follow-up contribute to higher percentage of participants who were active at 12 months.  12. NR. | 13. Brief interventions involving exercise on prescription; brief face-to-face advice from a HCP; telephone follow-up; or print-based support can increase activity levels.  14. This intervention could be set up in areas other than general practice. Implementation of this intervention would also increase if it was applied systematically to daily practice, with routine screening; delivery; and follow-up. |
| Gibson et al (2013).  Australia.  Syst rev. 10 studies included  Association between chronic disease type 2 diabetes related hopitalisations and primary health care resourcing. | 1. NR.  2. NR  3. NR | 4. Patients with chronic illness of type 2 diabetes.  5. Type 2 diabetes.  6. Primary Health Care.  7. Practice Nurse part of the primary health care team including GP. | 8. NR  9. NR | 10. NR.  11. NR.  12. NR. | 13. This study is inconclusive with regards to its outcomes. The impression from this body of work is that access to primary care (as distinct from use – which will be highly confounded by health status) is probably associated with a reduced rate of hospitalisation for diabetes-related ACSC.  14. “Collectively, study findings must still be considered inconclusive, and the relationship between PHC resourcing and hospitalisation for diabetes-related ACSC remains uncertain. Thus additional studies are needed that adjust for a wide range of potential confounders and consider more carefully how best to adjust for disease severity” |

| Harris et al (2015).  UK.  RCT.  To examine if a primary care nurse-delivered complex intervention increased objectively measured step-counts and moderate to vigorous physical activity (MVPA) in people aged 60-75. | 1. NR.  2. Behaviour change techniques and feedback from pedometers and accelerometers.  3 Components – pedometers; accelerometers; practice nurse consultations based on behavioural change techniques; patient handbook; individual walking/ physical activity plan; physical activity diary. | 4. People aged 60-75 years registered with 3 GP practices (n= 289).  5. No health problem.  6. General practices in Oxfordshire and Berkshire, UK.  7. Practice nurses. | 8. Average daily step count difference at 3 months – 1,037 (95% CI 513-1560) steps per day (P<0.001).  Difference in weekly time spent in moderate to vigorous physical activity (MVPA) at 3 month– 66 (95% CI 36-96) mins/ week (p<0.001);.  Difference in MVPA bouts >10 mins @ 3 months - 63 (95% CI 40-87) mins/week (p<0.001).  At twelve months figures were:-  Average daily step count difference 609 (95% CI 104-1115) steps/day (p=0.018); difference in time spent in MVPA 40 (95% CI 17-63) (p=0.001).  9. Sub group analysis suggests effects could be larger for men than women – 1534 (95% CI 775-2294) v 591 (95% CI 125-1307) (p=0.08) and stronger for couples 1750 (95% CI 850-2651) than individuals 692 (95% CI 61-1319) (p=0.06). | 10. Intervention requires more time, training and support than is currently standard in NHS health checks or routine care.  11. Participants and nurses enthusiastic about the intervention – as documented in an accompanying qualitative study.  Pedometers and accelerometers and goal setting and monitoring were seen as enablers of increased PA.  Two stage recruitment process used in this study (with screening out of unsuitable patients) is recommended.  12. Weather and existing health problems were seen as barriers to increased PA. | 13. The intervention increased both step counts and objectively measured MVPA in>10 bouts in 60-75 year olds at 3 and 12 months with no effect on adverse effects.  14. Practice nurses can safely deliver an intervention to increase objectively measured PA in older people and the intervention is acceptable to older people and nurses. Primary care is an ideal setting for delivering PA intervention.  Future research needed to distinguish different aspects of PA intervention i.e. to separate effects of pedometer, accelerormeter, and nurse consultation. Research also needed in socio-economically deprived populations and in older people with higher levels of morbidity and disability. Inclusion of a qualitative element is also recommended. Also robust evidence on cost and cost effectiveness should be gathered before similar programmes are implemented on a large scale in the health service. |
| --- | --- | --- | --- | --- | --- |
| Hoare et al. (2011).  Australia and New Zealand.  Systematic review (n=47)  To report *“on a review that examined the role of Government policy in primary care and its association with nurse-led care in the United Kingdom, New Zealand and Australia between 1998 and 2009.”* (p.963) | 1. A practice nurse “*is responsible for implementing prescribed programmes of care, working under the supervision of a GP* (Rashid et al. 1996)” (p. 972)  2. NR.  3. NR. | 4. Practice nurses; general population; patients; general practice staff; prevention clinic staff; primary care staff.  5. A range of government policies associated with nurse-led care.  6.General Practice; Primary Care; Prevention Clinics.  7. Practice nurses. | 8. The development of practice nurse; Government policy supporting quality improvement – clinical governance; Nurse practitioners in the UK; Nurse-led care in the UK; Education and career structure.  9. Strengthening frameworks for primary care nursing education and career pathways have been statistically significant  in the UK and NZ; Quality and Outcome Framework (QOF), contributed to an  improvement in patient and population-health outcomes. Nurse lead care has led to improved lifestyle; health status; fewer deaths; fewer coronary events; optimal care; reduced HbA1c; cholesterol levels; improved medication adherence; and has shown to be cost effective. | 10. Further funding programmes will benefit nurses in becoming leaders in the field of patient care; education; collaboration and networking.  11. Clinical governance structures and the “Quality and Outcomes Framework” were vital to the development of the practice nurse workforce in England.  12. Strain on GP employer and nurse employee relationships impede collaborative practice. | 13. New Zealand and Australia have not utilising their practice nursing resource. Standalone policies are ineffective in the development of nursing lead care. Career structure is limited for practice nurses in New Zealand and Australia.  14. Reducing heath inequalities is necessary, therefore, additional research is needed to investigate the role of nurse-led care in general practice. Funding in general practices in New Zealand and Australia need to assist in the development of the role of practice nurse or nurse practitioners. A “national quality framework with a plan for implementation may stimulate practice nurse development in New Zealand and Australia” (p.975). |
| Houweling et al. (2011).  Netherlands.  RCT.  To determine whether the management of type 2 diabetes mellitus in a primary care setting can be safely transferred to practice nurses. | 1. NR.  2. Primary Care Nurse care of diabetic patients in comparison to GP care.  2. Practice nurses who treated glucose levels, blood pressure and lipid profile according to a specified protocol (intervention group)  Conventional care: general practitioner. | 4. Adults with diabetes (n=230).  5. Diabetics.  6. General Practice.  7. Practice Nurses. | 8. Mean decrease in glycated haemoglobin (HbA1c) levels at the end of a 14 month follow-up period.  No significance with regards to between-group differences with respect to reduction in HbA1c, blood pressure and lipid profile.  Both groups indicated improvements with regards to HbA1c, Blood pressure, cholesterol and lipid profiles. Significance re decrease in blood pressure in both groups; 7.4/3.2 mm Hg (intervention group) and 5.6/1.0 mm Hg (control group).  Within both groups, more patients met the target values goals for lipid profile compared to baseline  With regards to levels of satisfaction patients treated by a practice nurse (intervention group) were more satisfied with their treatment than those being treated by a general practitioner (control group)  Within the intervention group, there was some deterioration recognised in the health-related quality of life and an increase in diabetes-related symptoms.  9. NR. | 10. Time needed to deliver Practice nurse led intervention as on average GP spent 28 minutes in comparison to practice nurses who spent 128 minutes per patient. However, this study argues the merits of this additional time resource being linked with increased patient satisfaction outcomes.  11. NR.  12. NR. | 13. The results show that a nurse, when following specific guideline protocols, achieves results which are comparable to those achieved by a GP with respect to blood pressure, glucose and lipid profile regulation. Furthermore, most of the results regarding the process indicators were remarkably better in the group cared for by a nurse.  14. Authors recommend PNs should be allowed to prescribe medications in The Netherlands, as is common practice in some other countries. |
| Jansink et al. (2013).  Netherlands.  RCT.  To determine if a nurse-led structured diabetes care with a protocol, record keeping, reminders, and feedback, plus training in motivational interviewing and agenda setting (intervention) was effective in comparison to usual diabetic care consistent with current diabetes guideline. | 1. NR.  2. Nurse led intervention focused on the provision of structured diabetes care with a protocol, record keeping, reminders, and feedback, plus training in motivational interviewing and agenda setting.  3. Protocol, record keeping, reminders, and feedback, plus training in motivational interviewing and agenda setting (intervention). | 4. Primary care nurses (58 practices) educationally prepared to deliver structured diabetic care and their patients with type 2 diabetes (n=940).  5. Diabetes type 2- structured care programme vs usual diabetic care.  6. General practice.  7. Primary Care Nurses. | 8. This study reports no statistically significant improvements in diabetic outcome measures compared with usual care. The study reports small changes in cholesterol outcomes.  The intervention group was no more effective than usual care in terms of the reported consumption of alcohol, fat, vegetables and fruit, or physical activity.  9. NR. | 10. NR.  11. NR.  12. NR. | 13. The intervention did not result in significant improvements.  14. Further research on lifestyle counselling embedded in primary care and the assessment of factors influencing the use of such counselling strategies is required.  The adaptation of a health protection approach or a personalised lifestyle counselling approach as opposed to motivational interviewing technique in targeting lifestyle change with this care group should be considered. |
| Tiessen et al. (2013).  Netherlands.  RCT.  To assess the costs and  cost-effectiveness of cardiovascular prevention by practice nurses from a societal perspective in both treatment groups of the SPRING-RCT study completed in 2009. | 1. NR.  2. Cardiovascular risk management.  3. The control group received standard treatment  according to the 2006 Dutch general practitioner’s guideline, conducted by specially trained practice nurses. The intervention group additionally received counselling based on self-monitoring at home, with pedometers, weighing scales and/or BP devices. | 4. Adults (n=179)  5. Patients with an elevated cardiovascular risk.  6. 20 general practice settings in the Netherlands.  7. Practice nurse (trained in cardiovascular management) in general practice. | 8. Total costs consisted of direct costs (medication, time spent by medical staff, self-monitoring equipment, patient transport to the practice) and productivity losses (absence at work of the working individuals that participated).  In this population, the costs of cardiovascular prevention were higher in the intervention group, with annual costs per individual of €160 (control group- standard treatment) compared with €335 (intervention group- self monitoring at home).  Costs per percent decrease in estimated 10-year cardiovascular mortality of €98 compared with €187, for the control and intervention group respectively.  9. Standard cardiovascular risk management by practice nurses is more cost effective than additional intensive counselling based on self- monitoring. | 10. For both groups costs predominantly consisted of societal costs and staff time and not of medication. As a considerable proportion of the target population for cardiovascular risk management consists of working individuals, productivity losses during  practice visits have to be taken into account when deciding about cardiovascular risk management strategies.  11. NR.  12. NR. | 13. Costs for cardiovascular risk management are found to be relatively low, for protocols based on the Dutch General Practitioner’s Guideline on Cardiovascular Risk Management (version 2006).  14. This study supports the use of the Dutch General Practitioner’s Guideline on Cardiovascular Risk Management (version 2006) for the targeted individuals in general practice. An added role for self-monitoring can be considered only for females and higher educated individuals. |
| van Dillen and Hiddink. (2014)  The Netherlands  Systematic Review (n=45)  To *“describe PNs’ actual role in lifestyle counselling in primary care and their cooperation with other health professionals”* (p.2) | 1. NR.  2. Lifestyle counselling.  3. NR. | 4.Practice Nurses (n=1 to n=606).  5. Weight Management.  6.General practice/primary care.  7.Practice Nurses. | 8. Interventions where the nurse’s main role is primary care provider larger effects on clinical outcomes are noted. Outcomes of these studies were divided into three categories: positive, neutral, and negative. 10 out of 12 intervention RCTs with PN achieved positive outcomes.  9. 26 studies identified that the role of the practice nurse in lifestyle counselling is needed; three interventions identified that PN spend more time with patients in consultation and therefore, patients were significantly more satisfied with PNs’ care; 6 intervention studies indicated that PNs can attain similar health outcomes as GPs for a range of diseases. | 10. 6 studies identified funding as barrier for “lifestyle counselling”.  11.Collaboration with several disciplines.  12. Time was recognized as a barrier to nurses giving lifestyle counselling in six studies. | 13. An MDT approach is more desirable to weight management care. It remains unclear as to whether a PN can provide care as a case manager in relation to lifestyle counselling in weight management. This may depend on context and situational issues.  14. Practice nurses need further training in case management of chronic diseases in primary care settings prior to it becoming an essential component in the role of the PN.  Transferring the responsibility of such care to PN may encourage therapeutic prospects for obesity management.  The value of PNs weight loss counselling may be improved by providing support in addressing barriers; securing support; and setting collaborative goals. |

# Home Based Community Nursing

| **Source**  **Country**  **Type of Evidence**  **Aim** | **1. Definitions**  **2. Overview of Models**  **3. Main components** | **4. Population Group and Size**  **5. Health Condition / Problem**  **6. Healthcare context / setting addressed**  **7. Nursing disciplines involved** | **8. Outcomes assessed and effects on outcomes**  **9. Components associated with improved outcomes** | **10. Resource Implications**  **11. Enablers**  **12. Barriers** | **13. Key conclusions**  **14. Recommendations for Practice, Policy, Education, Research** |
| --- | --- | --- | --- | --- | --- |
| **Preventative** | | | | | |
| Behm et al. (2014).  Sweden.  RCT.  To analyse the long-term effects of preventive home visits and senior meetings with respect to morbidity symptoms, self-rated health and satisfaction with health. | 1. NR.  2. Preventive home visits and group sessions with multiple health professionals (senior visits).  3. Three arm trial comparing preventive home visits x1 with ‘senior visits’ 4 weekly and one follow up home visit with usual care. | 4. Adults >80 living at home, not requiring help with ADL and no cognitive impairment (n=459).  5. Variable levels of frailty and deemed at risk of health decline/ increasing frailty.  6. Community.  7. Registered nurses working alongside physios, OTs and social workers. All received training for the study. | 8. Progression in morbidity significantly lower at 1 and 2 year follow-ups in both groups.  OR 0.44 (p = 0.001, 95% CI = 0.27–0.73) for PHV and 0.61(p = 0.048, 95% CI = 0.38–0.99) for senior meetings after 1 year and 0.60 (p = 0.035, 95% CI = 0.37–0.96) for the PHV and 0.52 (p = .008,95% CI = 0.32–0.84) after 2 years.  No sig. differences concerning the progression of symptoms.  Self-rated health significantly lower in the senior meetings group v controls OR 0.55 (p = 0.039, 95% CI = 0.31–0.97) .  Satisfaction with physical health sig lower at 1 and 2 year for both interventions. OR 0.49 (p = 0.015, 95% CI = 0.28–0.87) for PHV and 0.57 (p = 0.049, 95% CI = 0.32–1.00) for senior meetings, at 1 year; OR 0.43 (p = 0.013, 95% CI = 0.22–0.84) for the PHV and 0.28 (p = 0.001, 95% CI = 0.14–0.59) for senior  meetings at 2 yrs.  9. NR. | 10. NR  11. NR  12. NR | 13. A decline in health outcomes in very old persons at risk of frailty can be postponed. ‘Senior meetings’ have a greater effect than preventive home visits with respect to self-rated health.  14. NR. |
| Butterfield et al. (2011)  USA.  RCT.  “To test the effectiveness of a multi-risk social/cognitive intervention on rural low income parents’ (1) environmental health self-efficacy and (2) stage of environmental health precautionary adoption.” *(p262)* | 1. NR.  2. The translational environmental research  in rural areas (TERRA)  model: Assumes that interventions for reducing environmental risk have potential to positively impact both the frequency and magnitude of risks, as well as risk perceptions of family members perceptions.  3. Home visits. Interactive book guide for risk assessment; Health information to  families & precautionary  actions; Biomarking testing  Control group – received a  letter re: biomarking tests  and values for each risk. | 4. Rural family households with at least one child aged 7 yrs or less and with potable water from nonmunicipal source (n= 235).  5. Environmental health.  6. Rural community.  7. Public health nurses. | 8. At 3 months Self-Efficacy: increased for general health environment and all risk factors (e.g. radon, water contaminants) (P<.001).  Change in behavior –increased adoption of precautions for most risks (P <0.05).  9. NR. | 10. NR.  11. NR.  12 NR. | 13. Public health nurses’ home visits should address addressing environmental risk reduction practices in addition to their focus on parenting and personal development.  14. Practice: Broaden the focus on PHN to include environmental health. |
| Corrieri et al. (2011).  Germany.  Systematic Review  To determine if preventive home visiting is cost effective in preventing falls in the elderly. | 1. NR.  2. Preventive home visiting to elderly to prevent falls - models very varied.  3. Nurse led interventions included assessment of fall risk; assessment of vision, hearing, balance mobility, feet, foot wear, cognition, functional data/ ADL, medication use and muscle strengthening. | 4. Adults > 65 years.  5. Risk of falls.  6. Home visits  7. Geriatric nurses (1 study); nurse practitioner (1 study); trained district nurse (1 study); nurse unspecified (1 study). | 8. Preventive home visiting cost effective for falls prevention (3 studies); 1 did not show cost effectiveness and one showed cost effectiveness for 1 subgroup only  Cost per QALY in one study $20,383 (2008 prices); mean savings per patient in one study $2958 (2008 prices); cost per fall prevented in another study $133 (2008 prices); cost per fall prevented $1525 (2008 prices) in another study.  Results from the different studies were not combined.  9. NR | 10. This was a systematic review of cost effectiveness – see Q8.  11. NR.  12 NR. | 13. Home visiting for falls prevention in the elderly was not shown to be comprehensively cost effective. Cost effectiveness appears to depend on careful adaption of selected measures for certain settings in special environments for designated patients and disease patterns.  14. Some specific recommendations made to improve standardisation in future research. |
| DeSocio et al. (2013).  USA.  RCT. Secondary analysis  Effectiveness trial of the new mothers’ home visitation program. | 1. NR.  2. Nurse visitation to: (a) ‘increase healthy behaviors during pregnancy and through the child’s development, (b) enhance maternal responsiveness and parenting skills’, (Pg 163)  (c) ‘improve the life trajectory of mothers using education, employment, and delay of future pregnancies’. (Pg 162)  3. Control Group received ‘free transportation, developmental screening/ referral for their children at 6, 12, and 24 months of age’.  Intervention received ‘free transportation and developmental screening plus intensive nurse home visitation up to infants’ second birthdays’(pg 162)  The goals of the  7 Community nurse | **4.** Pregnant women (28 weeks gestation) first birth  aged 12 to 33 years (n=429)   1. Having two of three criteria for social disadvantage: (a) unmarried; (b) less than 12 years education (c) unemployed   6. County Health Department, and patients’ homes.  7 Community nurse | 8.Self-agency change, using the Pearlin and Schooler Mastery Scale  Chronological age. Age used to represent development;  The Shipley Institute of Living Scale of Intelligence administered to measure cognitive ability in relationship to chronological age  Educational attainment Discretionary household income.  Neighborhood poverty.  9. Self-agency increased between intake and 24 months in both the control group (3.01 to 3.11, p < .001) and the intervention group (3.00 to 3.22, p < .001). | 1. NR   11NR.  12. Data reflect the experiences of unmarried adolescent mothers of two decades ago, | 13 Findings supported the hypotheses- intervention including nurse visitation would influence self-agency change in a group of unmarried adolescent mothers.  14 NR |
| Friedman et al. (2014).  US.    Secondary analysis of data from two arms of a larger RCT.  To gauge the impact of a home visit nurse on specific activities of daily living compared to usual care. | 1. NR.  2. Monthly home visit by nurses.  3. Assistance with self-care management; medication management and education on activities of daily living.  Does not involve direct nursing care. | 4. Medicare patients with difficulties or needing assistance in performance of six specified activities of daily living (n=499).  5. Patients needing or receiving help with ADL and recent significant healthcare use.  6. Community dwelling,  7. Specially trained nurses in aging, geriatric care, medicines management and health behavior modification | 8. Difficulty in performing or needing assistance in 6 specified activities of daily living.  Improvement in bathing but not in other ADLs (31.6% v 40.4%, p-0.01) at 22months.  9. NR. | 10. NR.  11. NR.  12. NR. | 13. Insufficient confidence in findings to make any recommendations except need for more research.  14. Research to investigate impact of interventions on individual ADLs and not just global ADL scores. May be possible to determine retrospectively on other recent other studies reporting global data. Need for more qualitative research on older person’s perspective and to assess impact on ADLs of other interventions including goal setting, disease management and medication management using educational materials and physician-patient-family-nurse conferences. |
| Imhof et al. (2012).  Switzerland.  RCT.  To evaluate the effectiveness of the in-home consultation programme by advanced nurse practitioners in terms of quality of life, health indicators and healthcare utilization of individuals of 80 years and over. | 1. NR.  2. Advanced nurse practitioners visited participants at home.  3. 4 visits over 9 months to ascertain participants health concerns, offer health promotion; used evidence based guidelines to manage prevalent health concerns (e.g. mobility, vision, hearing, pain, nutrition, cognitive abilities, and bladder control. Action planning at end of each visit | 4. Adults > 80 (n=461 of 1,182 potential participants).  5. No specific health problems. Inclusion on age and residence only.  6. Community dwelling adults in an urban area of German speaking Switzerland.  7. APNs with masters degree in Nursing Science. Experienced in home care and gerontological nursing. Specifically trained to deliver intervention. | 8. No difference detected in quality of life measure.  Significant difference in incidence of acute events e.g. number of 3-month periods with at least one acute event was lower in the intervention group (116 v 168 RR 0.70 NNT = 4.3).  Significant reduction in number of 3 month periods with falls in the intervention group (74 v 107 RR 0.71, NNT =7.1 p=0.003).  Number of 3-month periods without hospitalizations was significantly lower in the intervention group (n=47, 23%) v control group (n=68, 33%, p=0.03, RR 0.70, NNT = 10.0).  Lower use of pharmacist consultations but no difference in use of other health professionals.  9. NR. | 10. Cost per participant of home visit programme $1,250 over 9 months which authors argue (without precise data) would be a net saving due to reduced healthcare utilization.  11. NR.  12. NR. | 13. Home consultation programme by ANPs can be effective in reducing adverse health outcomes (acute events, falls, and hospitalizations) although no effect on quality of life was shown.  14. Recommends further research to confirm these findings with different doses of APN within different time frames using objective outcome measures. |
| Ming Wen et al. (2012).  Australia.  RCT  To assess the effectiveness of a home based early intervention on children’s BMI. | 1. NR.  2. A staged home based intervention for mothers of infants designed to improve infant feeding techniques, eating habits, active play, reduce TV viewing time as well as improve family behavioural risk factors for childhood obesity.encourage healthier behaviours.  3. 8 home visits from specially trained community nurses at 1-24 months’ post birth. | 4. Pregnant women aged 16+ (n=667).  5. Pregnant women attending antenatal clinics.  6. Primary Care.  7. Community Nurses. | 8. Mean BMI was significantly lower in the intervention group than in the control group.  9. NR. | 10. NR.  11. NR.  12. NR. | 13. NR.  14. To prevent early onset of childhood obesity a range of potential risk behaviours needs to be tackled. |
| Paul et al. (2012).  USA.  RCT.  To compare standard newborn care with a home nursing visit (HNV) as the initial encounter for “well” breastfeeding newborns and mothers. | 1. NR.  2. One home visit to well postnatal breastfeeding mothers.  3. Home nursing visit within 2 days of discharge; | 4. Postpartum mothers intending to BF (34 wks) (n = 1154)  5. Unplanned health care utilisation and maternal wellbeing  6. US health care with standard care for postpartum women office based (OBC)  7. Nurses. | 8. Unplanned health care utilization for mothers and newborns; proportion of women/newborns seen within 2 days after discharge, breastfeeding duration. Maternal mental health, parenting competence, satisfaction with care Assessed at 2 wks, 2 and 6 mts.  Improved BF at 2 weeks (92.3% vs 88.6%) (P=.04) and 2 months (72.1% vs 66.4%) (P=.05) but not 6 months; more mothers and newborns seen within recommended 2 days of hospital discharge (85.9% vs 78.8%) (*P*=.002), Greater parenting sense of competence at 2 weeks (mean diff 1.43 [95% CI, 0.40-2.46] P=.007) and at 2 months (mean diff 1.44 [95% CI, 0.36-2.51] P=.009),  No differences in maternal mental health, satisfaction with care or unplanned health care utilisation.  9. NR. | 10. Cost of one home visit by nurse.  11. NR.  12. Lack of primary nursing care service. | 13. Home nursing visits are a safe and effective alternative to Office Based Care for the initial outpatient encounter  14. In settings where post discharge follow-up is less optimal, a timely visit provided by HNVs could produce more positive effects than in the current study. |
| Sharps et al. (2013).  USA.  RCT  To *“test the effectiveness of*  *a structured Intimate Partner Violence (IPV) intervention integrated into health department perinatal home visiting (HV) programs”* (p134). | 1. NR  2. Domestic Violence Enhanced Home Visitation Program (DOVE) vs usual care  3. DOVE: Based on an empowerment model, combining 2 evidence-based interventions: a 10-minute brochure-based IPV intervention and nurse home visitation- 3 DOVE prenatal and 3 postnatal visits. Usual care: standard home visiting and IPV protocols. | 4. Abused pregnant women (n=239), randomised to DOVE (n=124), or usual care (n=115).  5. Pregnancy, intimate partner violence.  6. Home visiting.  7. Home visitors.- professional disipline unspecified. | 8. Retention rates of abused pregnant women in perinatal HV programs.  Retention of women in program: retention rates from baseline for DOVE women were 91% at delivery, 77% at 3 months, 75% at 6 months, and 70% of  women at 12 months compared to UC 84% of women at delivery, 75% at 3  months, 72% at 6 months,  and 70% retained at 12 months.  9. Screening for IPV appeared to promote a beneficial relationship with the home visitor. | 10. Training to deliver intervention (4-hour training included information about IPV, particularly in pregnancy, importance  of screening and intervening  + DOVE intervention  4-hour training including use of the screening and assessment instruments, delivering the brochure-based DOVE intervention, developing individualized safety plan, strategies for revisiting and reinforcing safety plan at each home visit, and appropriate documentation).  11. Home visitors were persistent in maintaining contact with participants, and strategies to increase retentionrates included sending birthday  and holiday cards to every participant, which also assisted with notification of  address changes.  12. Telephone services with nurses was inconsistent due to financial restraint  Barriers to screening for IPV: home visitors’ fear of either  being a victim of violence from the abusive partner or having the client withdraw from HV because discussing  IPV would be too sensitive and intrusive for participants. | 13. Findings suggest that many abused pregnant  women who are screened for IPV will disclose their abuse histories and will remain in perinatal HV programs and  research programs that specifically address IPV.  Results indicate that home  visitors’ confidence in screening for IPV increased with additional training and opportunities to observe health care professionals screen women for IPV and educate women on IPV.  14. DOVE strategies  for engaging and retaining abused pregnant women should be integrated  into HV programs’ federal government mandates for the appropriate identification and intervention of women and children exposed to IPV. |
| Tappenden et al. (2012)  UK.  Systematic Review. (n=14 – 11 RCTs and 3 economic evaluations).  To evaluate the clinical and economic effectiveness of home-based, nurse-led health promotion programmes in the UK. | 1. Nurse led health promotion activities described as a complex intervention.  2. Nurse led health promotion.  3. (a) health education,  involving communication directed at individuals, families and communities to influence; (b) service improvement, involving quality and quantity of service; and (c) advocacy involving agenda-setting for healthy public policy. | 4. Adults >65 (n=5850).  5. Long-term medical/social needs at risk of admission to hospital, residential or nursing care e.g. heart disease, Parkinson’s disease, stroke, venous leg ulcers.  6. Home.  7. Trained nurses / health visitors with access to MDT. | 8. Admission to hospital, residential or nursing care, mortality, morbidity including depression, falls, accidents, deteriorating health status, patient satisfaction. Outcomes measured and reported differently between studies preventing meta-analysis.  9. Mortality: reduced the risk of death [odds ratio (OR) = 0.80, 95% confidence interval (CI) 0.68 to 0.95].  Also statistically significant effects favouring the intervention on Leg ulcer recurrence, Nottingham Health Profile, Caregiver Strain Index, GHQ. | 10. There is a dearth of good-quality economic studies available to inform decisions about the cost effectiveness  of home-based, nurse-led health promotion. At best a weak suggestion that the cost-effectiveness may be dependent on the population at whom the programme is targeted.  11.NR.  12.NR. | 13. Given the limitations of the current evidence base, it remains unclear whether home-based health promotion interventions offer good value for money.  14. Recommended appropriate training of nurses, and potentially other elements of a multidisciplinary team, but may have considerable implications in terms of costs and capacity.  There may be a role for qualitative research in identifying which components of the intervention patients value or derive benefit from. |
| Wen et al. (2011).  Australia.  RCT  *“To assess the effectiveness of a home-based early intervention on infant feeding practices and “tummy time” for infants in the first year of life”* (p.701). | 1. NR.  2. Intervention (IG): A home based early intervention programme designed to improve family and behavioral risk factors for childhood obesity. Control group: One follow up home visit only.  3. Assessment and education on infant feeding practices, infant nutrition, active play, family physical activity, nutrition and social support. | 4. First-time pregnant women aged 16+ followed through to 12 months after birth (n= 527).  5. Infant feeding.  6. Home visits.  7. Community nurses (trained by health promotion practitioners to deliver programme). | 8. IG vs CG - Improved feeding practices as follows:  Breastfeeding rates higher in IG at 6/12 & 12/12; (42.2% vs 32.1% and 21.0% vs 14.9%, respectively).  Median breastfeeding at 12/12 duration was 17 weeks (95% CI, 13.9-20.4 weeks) in IG compared with 13 weeks (95% CI, 10.1-15.6 weeks) in CG.  Later introduction of solids in IG (P.001 for trend), reducing the proportion of mothers who introduced solids before 6/12 by 12% (95% CI, 4%-20%), from 74% to 62%.  IG decreased the proportion of mothers using food for reward by 7% from 25% to 18% (P=.04) and increased the proportion of children drinking from a cup by 7% from 85% to 92% (P=.01), as well as reduced the proportion of children having a bottle to go to bed by 9% from 44% to 35% (P=.04).  IG decreased the age at which infants started tummy time (P=.03 for trend) and increased daily practice of tummy time by 7% from 76% to 83% (P=.05).  9. NR. | 10. Noted that the effects of the intervention on traditional service delivery models and comparisons of cost-effectiveness vs health benefits of a large-scale intervention are unknown and merit further investigation.  11. NR.  12. NR. | 13. Early intervention programmes need to be family focused and commence in antenatal period.  14. See Q13 above. |
| **Curative** | | | | | |
| Leiva et al. (2014).  Spain.  RCT.  *“To evaluate the effectiveness of a multifactorial adherence-based intervention in a primary care setting in lowering BP”* (p. 1683) | 1. NR.  2. Nurse-led motivational interviews.  3. Usual care not described. The multifactorial intervention consisted of five components delivered during three home visits:  Nurse-led motivational interviews; simplification of dosing regimen by a pharmacist; reminder packaging; social and family support; and BP self-measurement. | 4. Patients aged 18–80 years (n=221).  5. Uncontrolled essential hypertension.  6. Primary care centres.  7. 32 nurses in 28 centres. | 8. Primary outcome of systolic BP at 12 months showed no significant difference between groups (151.3 mmHg versus 153.7 mmHg, P=0.294), as were the reductions from baseline (5 mmHg versus 2.1 mmHg).  Secondary outcome in terms of Diastolic BP showed no significant difference between groups (83.4 mmHg versus 83.6 mmHg), as was the percentage in each group who achieved BP control according to ESC/ESH guidelines (15.8% [18/114] versus 9.2% [10/109], P=0.098).  9. NR. | 10. NR.  11. NR.  12. Wide variability in adherence to medication (53.2%–94.5%) may have been due to the characteristics of nurses and overestimation of the intervention effects. This may have resulted in the absence of statistically significant differences between intervention and control groups. | 13. The main finding of the study was that a multifactorial intervention in a primary care setting based on enhancing treatment adherence did not lead to the expected improvement in BP.  14. NR. |
| Watson et al. (2011).  UK.  RCT  *“To assess the clinical effectiveness of weekly*  *delivery of low dose, high frequency therapeutic*  *ultrasound in conjunction with standard care for hard to heal venous leg ulcers”* (p1). | 1. NR.  2. Low dose, high frequency therapeutic ultrasound in conjunction with standard care.  3.Intervention:Weekly administration of low dose, high frequency ultrasound therapy of 5-10 mins, delivered at weekly dressing changes, for up to 12 weeks plus standard care, vs standard care alone. After 12 weeks of ultrasound, patients returned to usual care only. | 4 Adults (n=337).  5.Venous leg ulcers.  6. Community and district nurse-led services, community leg ulcer clinics, hospital outpatient leg ulcer clinics.  7. Nurses. | 8. Primary outcome: time to  healing of the largest eligible leg ulcer. Secondary outcomes: proportion of patients healed by 12 months, percentage and absolute change in ulcer size,  proportion of time participants were ulcer-free, health related quality of life, and adverse events.  No significant differences detected regarding outcomes except that the number of non-serious adverse events was significantly associated with the treatment received, with more events in the ultrasound group than the standard care group (model estimate 0.35 (0.02 to 0.67), P=0.04).  9. NR. | 10. NR.  11. NR.  12. NR. | 13. No evidence to support ultrasound treatment for patients with leg ulcers.  14. Centres with highest staff recruitment rates had highest healing rates - further research into this correlation is recommended. |
| Weller et al (2013).  Australia.  Systematic review (n=2 RCTs).  *“To assess the benefits and harms of interventions designed to help people adhere to venous leg ulcer compression therapy, and thus improve healing of venous leg ulcers and prevent their recurrence after healing”* (p1). | 1. NR.  2. Community-based Leg Club® clinic vs home based care. Community based  nurse-led self-management programme (Lively Legs®) and usual care vs usual clinic care only.   1. Leg Club: peer-support, assistance with goal setting and social interaction   Lively Legs: six month programme promoting physical activity (walking and leg exercises) and  adherence to compression therapy via counselling and behaviour modification. | 4. Adults (n=67, Edwards, 2009; n=184, Heinen,2012).  5. Venous leg ulcers.  6. Community setting.  7. Nurses. | 8. Primary outcomes: venous ulcer healing, time to complete healing, recurrence of venous ulcer, adherence to compression therapy (e.g. proportion reporting adherence to compression).  Secondary outcomes: Quality of Life (QoL), adverse events, pain, economic outcomes.  9. Edwards (2009). Pain: Significantly decreased in Leg Club participants, compared with home care (MD -12.75 points on  100 point scale, 95% CI -24.79 to -0.71).  Heinen (2012). No significant differences reported. | 10. The incremental cost per healed ulcer to the service provider, carers, clients and community of the Leg Club was reported as AUD 515 at six months (the cost of usual care was estimated as AUD 1546). However, the paper did not report the effect estimate used in the analysis so could not be verified (Edwards, 2009).  11. NR.  12. NR. | 13. These trials did not reveal a benefit of community-based clinics over usual care in terms of healing rates, prevention of recurrence of venous leg ulcers, or quality of life. Not possible either to recommend or discourage nurse clinic care interventions over standard care.  14. Need further innovation in treatment methods and a better understanding of strategies to improve adherence to intervention. Recommends improved methods for reporting in trials in this area. |
| **Care management** | | | | | |
| King et al. (2012).  New Zealand.  RCT.  To evaluate the impact of a restorative home care service for community dwelling older people. | 1. NR.  2. Care management with paid caregivers providing restorative home care co-ordinated by experienced registered nurse.  3. Initial assessment; goal setting (using TARGET tool) regular visits by paid caregiver (not nurses). | 4. Older people (n=186. 93 in each arm).  5. In receipt of assistance from the home care agency.  6. New Zealand primary care.  7. Registered general nurses trained for the study. | 8. Primary outcome – health related quality of life measured by SF36. Intervention showed a statistically significant improvement at 7 months (mean difference 3.8, 95% CI 0.0-7.7, p=0.05). Secondary outcomes were physical, mental and social wellbeing. No changes in these seen. There was a statistically significant difference in the number in the intervention group identified for reduced hours or discharge (29% v 0% p<0.001). A significantly higher number of intervention older people had care giver’s hours reduced or were discharged from home care agency – 22 v 0 had hours reduced or were discharged  9. Greatest change in the mental health component of SF36 which were strongly influenced by social and emotional function elements of the mental health component. | 10. NR  11. NR  12. NR | 13. Restorative home care service may be of benefit to older people and improves home care efficacy. More likely that the paid caregivers’ regular contact had a greater impact on the older people’s mental health than the (nurse) co-ordinator.  14. NR. |
| Marek et al. (2013).  USA.    RCT.  To test the efficacy of nurse care coordination with or without medication dispensing technology in helping older adults self manage chronic illness. | 1. NR.  2. Nurses acted as care co-ordinators for elderly patients who were having difficulty managing their medicines.  3. Three arm study. One arm was nurse care coordinator plus a medication dispensing system (MD.2); one arm was nurse care coordinator plus a simple medication organizer box; one control arm. | 4. 414 patients over 60 in a Mid Western urban area (US).  5. Patients who were having difficulties managing their medicines (as detected on questionnaire instruments).  6. Patients discharged home health care from one of 3 home health care agencies in Milwaukee County.  7. APNs and RNs. | 8. Missed medication doses. Clinical status measured on SF36 Physical Component and Mental Component, Geriatric Depression Scale, MMSE, functional status PPT.  Average percent of correct doses per month was 98.8% (SD.30) in MD.2 and 97.4% (SD 5.19) in the medi-planner group . Comparison of MD.2 v medi-planner was non significant for all five clinical measures.  9. Components – nurse care coordination; aids to help with medication administration. Nurse care made a clear difference; aids did not. | 10. NR.  11. NR.  12. NR. | 13. Nurse care coordination has a beneficial effect on cognitive functioning, depressive symptoms, functional status and quality of life in both mental and physical functioning.  14. Investing in methods to assist older adults with medication and chronic disease management has the potential to improve the quality of life of older adults. |
| Marek et al. (2014).  USA.  RCT- further reporting on RCT by Marek et al (2013).  To determine whether a home-based care coordination program focused on medication self-management would affect the cost of care to the Medicare program and whether the addition of technology, a medication-dispensing machine, would further reduce cost. | 1. NR.  2. Home-based nurse care coordination program (NCC).  3. Involved comprehensive admission assessments and plans of care by APN & RN’s focused on supporting participants’ and their families’ self-management behaviors. Participants visited at least every 2 weeks to fill their pill organizer or medicine-dispensing machine and more frequently if their condition required additional visits. Two different devices (pill organizer and meds dispensing machine) were used to enhance medication self-management behaviors. | 4. Older adults (n=414).  5. Older adults with problems self-managing their chronic illnesses.  6. Primary Care – home visits.  7. Advanced Practice Nurses (APNs) and Registered Nurses (RNs). | 8. Cost: participant claims data from 2005 to 2011.  9. Nurse care coordination plus a pill organizer was a cost-effective intervention for frail elderly Medicare beneficiaries. The cost of the NCC plus pill organizer intervention, yielding a net savings of $3,552 per year. The cost of the NCC plus medication-dispensing machine intervention was higher per year. | 10. During the 12-month study period, mean monthly Medicare expenditures were higher in the intervention groups than in the control group.  11. Working in participants’ environments provided opportunities to observe barriers to self-management and to create interventions that were more viable for them to use in their self-management practices.  12. The machine group required additional visits related to the functioning of the machine, increasing the cost of the intervention. | 13. The active ingredient in the NCC intervention was the home visit.  14. Given the cost of care for chronically ill individuals and the consequences of care mismanagement, investment in systems to support self-management is essential.  Results are promising and support testing of the model in a larger, longer study. |
| Poortaghi et al (2011)  Iran  RCT  The focus of this study was to “*determine whether continuing cardiac rehabilitation programs at home has positive effects on psychological and general health of the participants’ in comparison with the control group*” p.407 | **1** Home based cardiac rehabilitation programme by community nurse for patients with cardiac diseases following uncomplicated heart attack  as a model of care focused on rehabilitation resulting in increased client independence and improved general health  CHD - coronary heart disease  **2.** Home based cardiac rehabilitation conducted by community nurses focused on development of self efficacy  **3.** Both groups provided with routine cardiac rehabilitation in the centre. Case group given additional education and training in conjunction with home visits by community nurse. | **4.** N=CHD patients referred to a rehabilitation centre (n=80)  **5.** CHD patients receiving rehabilitation  **6.** Rehabilitation centre staff and Community Nursing.  **7.** Community Nurses | **8.** The main outcome variables = measurement of general health subscales including  physical symptoms (p= 0.000) anxiety and insomnia, (p= 0.004) social function (p= 0.006) depression, (p= 0.000) showed statistically significant difference between the 2 groups indicating that the intervention improved patients overall general health in the subscales highlighted above.  **9**. Home based rehabilitation has a positive effect on patient’s general health.  Appropriate and effective training of patients, continuity of care and home follow up can relieve the difficulties caused by patients not referred to ambulatory rehabilitations centers.  Community nurses play an important preventative role in the provision of continuing education, accompanying patients and performing follow up care at home. | **10.** Educational preparation of community nurses to provide this programme  **11**. The ongoing health educational and supportive preventative intervention of community nurses providing the cardiac rehabilitation programme at home had a positive outcome on patients’ general health.  **12.** NR | **13**. This study’s key recommendations include  The provision of ongoing home rehabilitation due to the positive affect this program has on patients quality of life and general health  The provision of effective training of patients, continuity of care and follow on care provided by community nurses enhances patients general health .  **14.** This study advocates the merits of this intervention linked with improved quality of life and general health linked with continuity of care and follow on care. |
| Poortaghi et al. (2013).  Iran.  RCT - further reporting on RCT by Poortaghi et al (2011)  “*to evaluate the probable positive effects of continuing cardiac rehabilitation programs at home on self-efficacy of the patients with cardiac complications*” (p.1) . | **1**. NR.  **2.** Home based cardiac rehabilitation model of care focused on self efficacy resulting in increased client independence.  **3.** Patients referred to a rehabilitation centre. Randomly divided into case and control groups. | **4.** CHD patients referred to a rehabilitation centre (n=80).  **5.** CHD patients receiving rehabilitation following uncomplicated heart attack.   1. Home   **7.** Community Nurses. | **8.** Self-efficacy: Improved self-efficacy and better results in case group (P =0.003)    **9**. NR. | **10.** Educational preparation of community nurses to provide this programme.  **11**. NR.  **12.** NR. | **13**. Community health nurses have an effective preventative role. Home-based rehabilitation program has a positive effect on patients’self-efficacy.  14. The input of community nurses are recommended as important as their professional input is with regards to having a preventative focus, providing home care services, offering continuing educational input and providing follow up care at home.  Further studies required to reveal other effects of home-based cardiac rehabilitation such as well-being, return to work, further complications and other outcomes are recommended. |

| **Case Management** | | | | | |
| --- | --- | --- | --- | --- | --- |
| Aragones et al. (2012).  Spain.  RCT (n=338).  *“To assess the effectiveness of a multi-component programme to improve the management of depression in primary care” (*P. 297) | 1. NR.  2. The intervention consists of a multi-component programme based on he chronic care model adapted to primary care.  3. Components are of a training based organizational clinical and health related educational nature. Nurses act as case managers. Care guided by NICE 2008. Carefully scheduled contact with doctors and nurses. Primary Care psychiatry interface. Patient and family education by nurses | 4. Adults 18 + Years.  5. Major depression .  6. Primary Health Care Service.  7. General Trained Primary Care Nurses. | 8.The severity of depression (mean Patient Health Questionnaire -9 score) was 1.76 points lower in the intervention group [7.15 vs. 8.78, 95% CI=-3.53 to 0.02, p=0.053]. The treatment response rate was 15.4% higher in the intervention group than in the controls [66.9% vs 51.5%, odds ratio 1.8, 95% CI=1.2 to 3.1, p=0.011], and the remission rate was 13.4 per cent higher [48.8% vs 35.4%, odds ratio 1.8, 95% CI=1.1 to 2.9, p=0.026].  9. The mental health component of quality of life evolved more satisfactorily in the intervention group in parallel to a clinical improvement in depressed mood. | 10. Resource neutral.  11. NR.  12. NR. | 13. The programme for managing depression in primary care was designed so that – if effective – it could be applied in habitual care and is straightforward to implement.  14. NR. |
| Wagg et al. (2014).  Canada.  Systematic Review (RCT’s and quasi experimental studies).  To create using evidence from a systematic review qualitative data and expert consensus an internationally applicable service specification for continence care. | 1. NR.  2. Continence service design.  3. Case coordinator takes an advisory and facilitative role filling in gaps and coordinating care where available. | 4. Community dwelling patients with either bladder or bowel incontinence (n= unspecified)  5. Incontinence.  6. Community.  7. Community Nursing. | 8. A number of themes related to current and potential future organisation of continence care were identified from the data. A modular service specification with eight core components was created including case detection, initial assessment and treatment, care coordination, caregiver support, community based support, specialist assessment and treatment, use of containment products and use of technology.  9. NR | 10. A recurring theme was the low priority of continence care in a time of restricted financial resources.  11. NR.  12. NR. | 13. Ensure robust referral pathways, shift assessment for case coordination to nurses specializing in continence care, promote self-management and technology, use comprehensive assessment tools and service performance targets based on outcome and operational measures.  Services should be integrated across primary and secondary health care settings.  14. Specialists in continence care should focus on those with severe symptoms or who are unresponsive to conservative treatment strategies. |
| **Mental Health** | | | | | |
| Bruce et al. (2015).  USA.  Cluster RCT  To determine whether improvements in depressive symptoms occurred in those randomized to a Care Path intervention by Home Health Care Nurses. | 1. NR.  2. Depression Care path for patients at home - Guideline based treatment combined with care management.  Delivered by home health care nurses trained to manage depression  3. Weekly assessment; Suicidal risk protocols; clinical protocols; short term goal setting; monitoring. | 4. Adults 65+ (n=306).  5. Those screened positive for depression.  6. Primary Care.  7. All home health care nurses | 8: Depression severity (adjusted HAM-D score), disability (ADL); medical burden (Chronic Disease Scale) in depression care path vs usual care over 3,6 and 12 months.  9. The 12-month HAM-D score difference reached statistical significance (8.7 vs 10.6 (P=.05). No intervention effect among patients with mild depression (HAM-D score, <10).  Half of the sample were taking anti-depressants. Exploratory analyses found no difference in the effect of  the intervention by whether or not patients were already taking antidepressants | 10. NR.  11 Care path was designed to fit within routine nursing practice so depression management was integrated into scheduled visits  Protocol was simple to integrate into commercial clinical software systems  12. NR. | 13. Evidence that medical home care nurses can effectively integrate depression care into routine practice.  14. NR. |
| Chien et al. (2015)  Hong Kong  Randomized Controlled Trial  To test and evaluate the effectiveness of an adherence therapy (AT) for outpatients with schizophrenia spectrum disorders, based on a motivational interviewing (MI) approach over a six-month follow-up period. | **1.** A therapy to improve adherence to medication  MI therapy involving cognitive, motivational, insight inducing and behavioral training  **2.** Eight two-hour sessions were held at participants homes every two weeks  **3**. Phase 1 – goal and action setting for change  Phase 2 – Education, identifying barriers and develop coping strategies  Phase 3 – rationalize concerns and engage social supports. | **4.** Adults 18+ years (n=114)  **5.** Schizophrenia spectrum disorders  6. Community Psychiatric Nursing Service  7. Psychiatric Nurses | 8. Participants reported significantly greater improvements in their insights into illness and or treatment, psychosocial functioning, symptom severity, number of hospitalizations and medication adherence when compared to TAU  9. There was an effective reduction in symptom severity, re-hospitalizations, improved medications adherence, functioning, insight and treatment over a 6 month period. | 10. NR  11. The patients who volunteered to participate were mainly full or part-time employed, had a relatively shorter duration of illness and had satisfactory family support, consequently tey may have been highly motivate to engage in AT  12. NR | 13. Evidence suggests that using AT which is a structures and self-empowering model of psychosocial intervention used in conjunction with psycho-pharmacological and psychiatric treatments has explicit benefits to patients with schizophrenia and other psychotic disorders.  14. Further study on the effects of AT in people with psychotic disorders in terms of diverse sociodemographic and illness characteristics and longer term follow up period is recommended. |
| Toot et al. (2011).  UK.  Syst. Rev. (n=4) (Cohort studies (n=3) and descriptive (n=1))  To report on the effectiveness of Crisis Resolution/Home  Treatment Teams (CRHTTs) for older people with mental health problems | 1. NR.  2. Crisis resolution/home  treatment teams.  3. Community oriented old age psychiatry service.  Multidisciplinary Support team in a day hospital. | 4. Adults >65 years (n=317).  5. Older people referred with mental health problems  6. Community / day care.  7. Multidisciplinary support team | 8. Number of admissions to hospital, LOS, maintenance of community residence, use of services and cost.  9. 69% of referrals (n=70) were admitted in the intervention group compared to 100% (n=65) in the comparison group (statistically significant) (x 1 study).  A higher percentage of people remained at home after two years follow up in the 24-h crisis service group (49%; n=69) compared to the overall comparison group (35%; n=42) (2 studies). | 10. Cost per patient per month in an outreach support team was £823, which was lower than the £1814 cost  per patient per month on an inpatient ward (1 study only).  11. NR.  12. NR. | 13. Very little robust evidence (Level C) indicating that crisis resolution/home treatment services for older people with mental health problems reduce the number of admissions to hospital.  For all other outcomes, including maintenance of community residence and length of hospital stay, the evidence is very weak  14. Further research into crisis resolution interventions for older people with mental health problems should be based on sound theory, so that a robust evidence base can be created to drive future policy development. |
| Ukawa et al (2011)  Japan  RCT  To determine whether mini mental state examination (MMSE) scores improved  in older participants of a Functioning Improvement Tool (FIT) home visit programe. | 1. NR.  2. Use of the Functioning Improvement Tool (FIT)  3. During a Home visit  Medical personnel help the person look back on the previous day’s tasks  Using a six step approach: Visualise activity and Describe feelings | 4. Older People >65 (n=199).  5. Receiving preventive services or a community long term care prevention project.  6. At home.  7. Five nurses and one dental hygienist trained in the appropriate use of the FIT | 8. MMSE scores  Demographic information  9. MMSE scores in the intervention group at the end of follow-up were significantly improved compared with those at baseline (from 24.2 ± 4.3 to 25.0 ± 4.8, p = 0.004). | 10. NR  11. A non-pharmacological  intervention. Most effective in persons with mild cognitive decline.  12. NR | 13. FIT home‐visit program improved MMSE scores in older participants with mild cognitive  decline  14. Results suggest the  possibility of reducing long-term care expenses with the FIT home visit program; thus, future studies including  Cost-effectiveness evaluation is needed. |
| **Palliative Care** | | | | | |
| Aydede et al. (2014).  Canada.  Systematic Review (n=17 studies i.e. 10 cohort, 4 non- compliance, 2 cross-sectional and 1 randomized).  To identify studies examining home care intervention’s among adult CKD patients incorporating all outcomes. | 1. CKD was conceptualized as consisting of five stages following the Kidney Disease Outcomes Quality Initiatives definition. This suggests that CKD could be classified by treatment type: kidney transplant recipient, CKD independent of dialysis and CKD on dialysis.  Home Care “is an array of services which enables clients, incapacitated in whole or part, to live at home, often with the affect of preventing, delaying or substituting for long-term or acute care alternatives” (Pg. 2).  2. Most studies focused on nurse assisted home care with patients on dialysis.  3. NR. | 4. Chronic Kidney Disease Patients (n=15058).  5. Chronic kidney disease.  6. Home Care Setting.  7. General Nurses working in homecare setting with people with kidney related conditions. | 8. In peritoneal dialysis studies with comparators peritonitis and technique survival rates were similar across home care assisted patients and comparators. The risk of mortality however was higher for home care assisted PD patients  9. NR. | 10. NR.  11. NR.  12. NR. | 13. Home care may be helpful in providing a more efficient and higher quality care for CKD patients however a synthesis of evidence in this regard has not been undertaken. While this study presents a synthesis of the literature regarding home care in chronic kidney disease, specific gaps in the literature are identified and the true impact of providing home care for this population remains uncertain.  14. There are significant gaps in the literature. In particular further research is needed in the areas of home support for activities of daily living, palliative care at home or respite care for the caregivers of CKD patients. |
| Gomez et al (2013).  UK.  Systematic Review N=23 studies i.e.16 RCTs, 6 of high quality),  *“a. To quantify the effect of home palliative care services for adult patients with advanced illness and their family caregivers on patients’ odds of dying at home; b. to examine the clinical effectiveness of home palliative care services on other outcomes for patients and their caregivers such as symptom control, quality of life, caregiver distress and satisfaction with care; c. to compare the resource use and costs associated with these services; d. to critically appraise and summarise the current evidence on cost-effectiveness”.* | 1. Home Palliative Care Services: A team delivering home palliative care with the following four elements.  (i) Primarily for patients with a severe or advanced disease (malignant or non-malignant), no longer responding to curative/maintenance treatment or symptomatic (or both), or their family caregivers, or both. (ii) Aiming to support patients or family caregivers, or both, outside hospital and other institutional settings as far as possible and to enable patients to stay at home. (iii) Providing either specialist or intermediate  palliative/hospice care, as defined in a previous systematic  review and (iv) Providing comprehensive care and aiming at different  physical and psychosocial components of palliative care.  2. Palliative Home Care serices.  3. As above (Q1). | 4. People living with advanced illness (n=37, 561) and/or their family caregivers (n=4042).  5. Mainly advanced cancer but also congestive heart failure (CHF), chronic obstructive pulmonary disease (COPD), HIV/AIDS and multiple sclerosis  (MS), among other conditions.  6. Home.  7. Nurses. To be considered specialist one or more nurses must have undergone higher specialist training, | 8. Primary outcome - Death at home.  Meta-analysis showed increased odds of dying at home (odds ratio (OR) 2.21, 95% CI 1.31 to 3.71; Z = 2.98, P value = 0.003; Chi2 = 20.57, degrees of freedom (df ) = 6, P value = 0.002; I2 = 71%; NNTB 5, 95% CI 3 to 14 (seven trials with 1222 participants, three of high quality)).  Secondary outcomes included:  time the patient spent at home,  satisfaction with care, pain,  other symptoms, physical function, quality of life, caregiver pre- and post-bereavement outcomes.  Narrative synthesis showed evidence of small but statistically significant beneficial effects of home palliative care services compared to usual care on reducing symptom burden for patients (three trials, two of high quality, and one CBA with 2107 participants) and of no effect on caregiver grief (three RCTs, two of high quality, and one CBA with 2113 caregivers).  Economic data included:  hospital costs, other institutional care costs, community care costs, informal care costs, equipment and medication prescribed.  Evidence on cost-effectiveness (six studies) is inconclusive.  9. NR | 10. NR  11. NR  12. NR | 13. Results provide clear and reliable evidence that home palliative care increases the chance of dying at home and reduces symptom burden in particular for patients with cancer, without impacting on caregiver grief. This justifies providing home palliative care for patients who wish to die at home. New home palliative care interventions must respond to the challenges ahead, posed by rapidly ageing populations with increased complexity and growing need for home palliative care.  14. More work is needed to study cost-effectiveness especially for people with non-malignant conditions, assessing place of death and appropriate outcomes that are sensitive to change and valid in these populations, and to compare different models of home palliative care, in powered studies.  Although the findings on cost-effectiveness were inconclusive, even evidence of no effect onother outcomes with slightly higher costs would be enough to justify the existence of home palliative care services as they exist to fulfill the desire of many people to live at home in the last days of their life.  Policy makers and service planners can now calculate the extent to which current home palliative care services may need expansion  locally on the basis of current and target home death rates, applying NNTB from this meta-analysis to current and projected local need.  Further meta-analyses on symptom burden and  caregiver grief would produce more robust data Future studies need to harmonise measurement and reporting practices. |
| Hudson et al. (2013).  Australia.  Phase III parallel group (three-armed)RCT  To *“prepare caregivers for the role of supporting a patient with*  *advanced cancer receiving home-based palliative care by offering a one-to-one psycho-educational*  *intervention”.* | 1. NR.  2. Intervention based on transactional model of stress and coping. Two variations based on whether the person received 1 or 2 visits. Control group – usual palliative care support.  3. Intervention delivered over 4 weeks and involved phone calls with family, a family caregiver guidebook, written information about the intervention, assessment of caregiver needs, development of a care plan with caregiver, patient and team, home visits, education resources, strategies to promote wellbeing, preparation for death and information about bereavement and evaluation. | 4. Primary family caregivers >18 years (n=298).  5. Caregivers of patients with advanced cancer receiving home-based palliative care.  6. Home.  7. Family Caregiver Support Nurse (FCSN) who assisted the local palliative care service to assess caregiver needs, establish a care plan and provide additional caregiver support. | 8. Relative to participants in the control group; the psychological well-being of participants in the intervention condition improved by a small, non-significant amount. No significant reduction in unmet needs or improvements in positive aspects of caregiving amongst the intervention group were identified.  9. The intervention demonstrated significant improvements in participants’ levels of preparedness and competence when the intervention consisted of 2 visits (as opposed to 1). Effect sizes of the one visit, two visits and both groups combined relative to the control group were 0.08, 0.28 and 0.18 | 10. Training of FCSN.  11.NR  12. NR | 13. Relatively short psycho-educational interventions can enable family caregivers to feel more prepared and competent in the role of supporting a dying relative.  14. Further investigation is required to determine the longer term outcomes of such interventions.  Constructs such as preparedness and competence may be linked to overall psychological wellbeing. Further empirical work is required to explore this.  Future research is needed to identify the levels and criteria by which caregivers’ coping responses might be considered maladaptive and therefore potentially amenable to intervention. |
| Luckett et al. (2013)  Australia.  Syst rev and meta-analysis (n=10 articles reporting 9 studies – 2 RCTs, 1 NRS and 6 retrospective record reviews).  *“To establish whether community specialist palliative care services (SPCS) offering home nursing*  *increase rates of home death compared with other models”.* P. 279 | 1. Nursing care can be independent in nature (e.g., nurse practitioner-based models where nurses diagnose and prescribe care) or dependent (i.e. based on delegation). Other models include substitution (e.g. physician assistants) and enhancement, such as in nurse-coordinated models  2. Community-based SPCS providing home nursing for people with life-limiting illnesses.  3. NR | 4. People with life limiting illness.  5. Cancer and other life limiting illnesses.  6. Home.  7. NR. | 8. Meta-analysis indicated a significant effect for SPCSs with home nursing (odds ratio 4.45, 95% CI 3.24e 6.11; P < 0.001). However, the high-quality studies found no effect (odds ratio 1.40, 95% CI 0.97e 2.02; P <0.071). Bias was minimal.  9. NR | 10. The resource requirements of providing community palliative care through generalist services (e.g. primary care providers and community nurses in partnership with domiciliary carers) should not be underestimated.  11. Preferences for place of care and/or death are only true choices where they occur in the context of best-practice services in all settings.  12. When services are scarce, home deaths may occur simply because inpatient services are not available as an alternative.  Patients who lack a home caregiver and who are socioeconomically disadvantaged are less likely to have access to community  A better understanding of the barriers and facilitators for remaining at home is  needed, including detailed exploration of known drivers of hospital admission near the time of death, such as problems with symptom  management. | 13. Caregivers have highlighted home nursing as the most important service component, but it is also likely to be the most resource intensive. Evidence inconclusive that community SPCSs offering home nursing increase home deaths without compromising symptoms or increasing costs.  14. Future trials should compare the relative efficacy of different models and intensities of SPCSs.  The role of the SPCS nurse vis-a-vis other community service providers is of special interest. Research needed to guide optimal specialist-generalist communication and coordination.  Future evaluations should provide detailed cost-effectiveness data to inform the optimal combination of specialist and generalist provision, the role of nurses vs. other health professionals, and the necessary scope of practice and expertise in different service contexts.  A better understanding also is needed of who will benefit most from community-based  SPCSs and the configuration of nursing services required to meet patient and caregiver  needs.  Health professionals assessing the suitability of individual patients for home care would be assisted by validated tools for evaluating the viability of home death. Tools for assessing prognosis (with the aim of avoiding acute care admissions for people who are dying and prefer to die at home) also are required.  Improved targeting of services will become increasingly important as Western populations age and informal caregivers diminish in availability and capacity.  Even where evidence-based support for home care is available, patients and family caregivers should remain at liberty to choose the place of care and death. Need to support families better in making an informed decision between home vs. institutional care, acknowledging that preferences may change over time and may differ for patients vs. caregivers and place of care vs. place of death. |

| Parker et al. (2012).  UK  Systematic Rev.iew (n= 37 papers on 26 studies of which 11= RCTs & 15 = health economic studies) | 1. Care closer to Home: “any model of care  that acted to prevent immediate inpatient admission and/or enable a reduced length of stay for children (up to the age of 18 years) with acute, chronic, complex or palliative care needs” (p. 3).  2. Most common model is generic community children’s nursing teams & condition specific services, nurse led only or multidisciplinary teams.  3. NR. | 4. Children care. Samples in studies ranged from 15 to 210.  5. Long-term conditions/ short term health needs/in need of palliative care.  6. Home care.  7. NR for some studies reviewed. Nurses/primary care provider/case manager where reported. | 8. Paediatric home care has equivalent clinical outcome results to in-patient hospital treatments e.g. Weight gain in premature infants; HBA1C for diabetes care; Adaptation to school 7 work re mental health problems; Readmissions to hospital; Adverse events from chemotherapy.  Costs: Savings associated with paediatric home care.  9. NR. | 10. Care closer to home offers potential for reduced health service costs, but this is sensitive to case mix, skill mix and change in the local health economy.  11. NR.  12. NR. | 13. See Q14  14. Research: Areas for further investigation on care closer to home (CCTH) in minority communities or socio-economically deprived groups and in rural areas. Consider factors influencing  whether families feel able to use CCTH services, the views of children about service and and the impact of CCTH on primary and community care services. |
| --- | --- | --- | --- | --- | --- |
| Uitdehaag et al. (2014)  Netherlands  RCT  *“To compare nurse-led follow-up at home with conventional medical*  *follow-up in the outpatient clinic for patients with incurable primary or recurrent esophageal, pancreatic, or hepatobiliary cancer”. P518.* | 1. NR  2. Conventional medical  follow-up at the OPD clinic or nurse led follow-up at home.  3. Nurse-Led Follow-Up:  Performed by home visits of a specialist nurse,  Follow-up at 14 days and then monthly after randomization, up to 13 months or death. If necessary, telephone  contact was possible between visits.  The nurse-led care focused primarily on relief of suffering and complaints    Conventional Medical Follow-Up: Appointments  At the OPD clinic. Follow-up after one month and then every two months after randomization, up to 13 months or death. If patients were unable to come to the hospital, appointments could be done by telephone. | 4. Consecutive patients (n=138)  5. Unresectable or recurrent upper GI cancer  6. At home  7. Specialist nurse, with more than 10 years’ experience in oncology  nursing care. The nurse worked under the guidance of the attending medical specialist(s) and had regular contact with both the attending physician and the patient’s general practitioner. | 8. Outcome measures were based on structured questionnaires assessing patient (and relative) satisfaction, health-related QoL (HRQoL), and health care consumption (use of GP and hospital admission). Patients in the nurse-led follow-up group were significantly more satisfied with the visits, whereas QoL and health care consumption within the first four months were comparable between the two groups. Nurse-led follow-up was less expensive than conventional medical follow-up. However, the total costs for the first four months of follow-up in this study were higher in the nurse-led follow-up group because of a higher frequency of visits.  9. NR | 10. NR  11. NR  12. NR | 13. Results suggest that conventional medical follow-up is interchangeable with nurse-led follow-up. A cost utility study is necessary to  determine the preferred frequency and duration of the home visits.  Palliative care for patients with upper GI cancer can be provided at home by specialized oncology nurses with high patient and relative satisfaction. Findings also document that this strategy is less costly per visit than the conventional medical follow-up.  The higher level of satisfaction is largely determined by the fact that the home visits were perceived as less burdensome compared with the visits to the outpatient clinic. This is probably related to the burden of travel to the hospital, delays in physicians’ schedules, and the fact that most patients prefer to receive palliative care at home and prefer to die at home.  14. Further research on the frequency and the duration of visits is necessary in a cost utility study. Because the costs are largely determined by travel time and travel expenses, a study also should investigate if the home-based follow-up can be performed by nurses with less travel time and costs (e.g., community nurses) or by innovative interventions such as tele-health. |

| **Nurse-Family Partnerships/ VoorZorg nurses** | | | | | |
| --- | --- | --- | --- | --- | --- |
| Mejdoubi et al. (2013).  Netherlands.  RCT  To assess the effect of  VoorZorg, the Dutch Nurse-Family Partnership (NFP)on addressing self-reported Intimate Partner Violence (IVP) victimization and  perpetration among young, low-educated pregnant women and mothers compared with young mothers receiving the usual  care in the Netherlands. | 1. NR.  2. Intervention group received usual care and home visits from nurse throughout pregnancy, and until child turned 2. Control group received usual care.  3. Usual care includes physical examination, monitoring development of foetus, health education, postnatal advice, and monitoring of infant’s health.  Home visit intervention offered up to 50 structured standardized home visits (10 in pregnancy, 20 first year, 20 second year). Included raising awareness of IPV, identifying abusive relationships, communication, negotiation and emotional regulation strategies, reducing stress, aiming to increase women’s financial independence, and housing assistance. | 4. Disadvantaged pregnant women <26 years, with no previous live births. (n= 460). Randomised to intervention (n=237) or control (n=223).  5. Intimate partner violence, pregnancy.  6 Home care.  7. Home visits : VoorZorg nurses. Usual care: midwives/ obstetricians. | 8. Primary outcome: self-reported psychological, physical or sexual violence, and injury towards the participant (victim) as well as towards her partner  (perpetrator). Secondary outcomes were a summation of forms of violence, and both experiencing and perpetrating IPV.  9. Home visit program was effective in reducing victimization by IPV. Improvement greater in intervention group than control group at 32 weeks of pregnancy in relation to level 2 psychological aggression (C: 56% vs. I: 39%), physical assault level 1 (C:58% vs. I: 40%) and level 2 (C: 31% vs. I: 20%), and level 1 sexual coercion (C: 16% vs. I: 8%).  Reduced perpetration of IPV, with greater reduction in intervention group, in relation to level 2 psychological aggression (C: 60% vs.I: 46%), level 1 physical assault (C: 65% vs. I: 52%), and level 1 injury (C: 27% vs. I: 17%).  At 24 months after birth, IPV victimization was significantly lower in the intervention group for level 1 physical assault (C: 44% vs. I: 26%), and IPV perpetration was significantly lower for level 1 sexual assault (C: 18% vs. I: 3%).  Intervention appears to improve outcomes as it addressed risk factors for IPV (stress), and specific factors (identifying abusive relationships). | 10. NR.  11. Open and non-judgemental dialogue between nurse and patient; trusting and professional relationships with HCP  12 NR. | 13. Home visiting interventions shown to be successful in reaching high risk young pregnant women and identifying at risk individuals.  Suggests the need for healthcare professionals to focus on this group due to their vulnerability.  14. Need to screen at risk individuals/ groups and address IPV risk factors e.g. alcohol, financial dependency. Need for open dialogue and non-judgemental therapeutic relationship with nurse.  Need to reduce impact of IPV where it is already present. Must address violent and controlling behavior among perpetrators, as this can lead partner to also use violence.  Further research should examine how to decrease loss of follow-up in low income pregnant women.  Future interventions should address perpetration of IPV by women. |
| **Mejdoubi et al 2014.**  Netherlands  RCT – further reporting on RCT by Mejdoubi et al (2013)  To assess whether Voor Zorg programme is effective in reducing smoking in young high risk pregnant women, also effect on pregnancy outcomes and breastfeeding at 6 mths. | 1. NR.  2. VoorZorp programme (NFP/USA) to address risk factors and prevent child abuse.  3. 40–60 home visits from pregnancy until two yrs after birth | 4. 460 Pregnant women with low SES: < 25 yrs, low education, 28/40, nullips, + risk factors (no social support, hx domestic violence, psychosocial symptoms, unwanted/unplanned pregnancy, financial/housing problems, unemployment, drug/alcohol use)  5. Cigarette smoking, birth outcomes, BF at 6 mts.  6. Youth health care organisation (ambulatory and well-baby services for monitoring child health and development and promoting positive parenthood).  7. Nurses trained in the programme. | 8. Cigarette smoking status, infant wt and gest, BF at 6mts assessed at 16, 28, 32 wks preg + 2 and 6 mts postnatal.  9. Reduced smoking and prolonged BF in intervention group. | 10. Funding for programme.  11. NR.  12. NR. | 13. Programme effective at reducing cigarette smoking and increasing BF duration. No effect of pregnancy outcomes.  14. NR. |
| Robling et al. (2015).  UK.  RCT  To *“assess the effectiveness of giving the programme to teenage first-time mothers on infant and maternal outcomes up to 24 months after birth”* (p.1) | 1. NR.  2. Family Nurse Partnership (FNP) intervention. Control group: Usual care only.  3. Usual care consisted of screening, immunisations, health education, and support until the child turned two.  FNP: Up to 64 home visits by nurse (average 39). | 4. Nulliparous pregnant teenagers aged ≤19 years, at <25 weeks gestation (n=1,645). Randomised to intervention group (n=823) and control group (n=822).  5. Pregnancy  6. Community midwifery partnerships (local authorities, primary and secondary care organisations)  7. Trained family nurses | 8. Primary: tobacco use in late pregnancy, birthweight, emergency attendance and hospital admission of infant within 24 months of birth, and proportion of women with second pregnancy within 24 months.  Secondary: measures of pregnancy and birth, child health and development, and parental lifecourse between birth and 24 months postpartum.  9. Tobacco use: no difference between groups in number of women smoking in late pregnancy (56%). No difference in number of cigarettes per day for women classified at baseline as smokers.  Hospital visits for child: 81% in FNP, 77% in controls.  Second pregnancy within 24 months: No difference between groups (66%).  Secondary outcomes suggested small positive effects of FNP: intention to breastfeed, maternally reported cognitive and language development, levels of social support, quality of relationship with partner, and self-efficacy. Greater documentation of child safe-guarding concerns in FNP group. | 10. Incremental cost for FNP ￡1993 per participant. Individual types of resource use were similar across trial groups, intervention delivery costs (FNP calls and visits) accounted for the substantial  incremental cost of FNP. Sensitivity analysis of only complete cases o (217 in FNP group *vs* 186 in  usual care group) suggested the incremental cost of the FNP was ￡4670 (95% CI 3322–6017). Not cost-effective for main outcomes – smoking cessation, and second pregnancies.  11. NR.  12. NR | 13. Substantial additional cost, without benefit for main outcomes, and only slight  advantage for some secondary outcomes after adding FNP to existing services  14. Longer term follow up may provide evidence for greater child development benefits. |

| **Miscellaneous** | | | | | |
| --- | --- | --- | --- | --- | --- |
| Alicea-Planas et al (2013)  Randomised experimental (pretest/posttest intervention) trial.  USA  To investigate whether self efficacy and self-rated health would improve following a goal setting intervention by nurses during primary care visits , for Spanish speaking patients | 1 NR  2 Goal setting intervention  Chronic care model (CCM) used as theoretical framework- focuses on empowerment and support to self manage health  3 ‘Healthy changes’ action plan - identified small health goal, and identifie3d barriers and facilitators to this goal. Rated confidence in ability to achieve same.  Follow up appointment at one month with reminder telephone call 1-2 days before. | 4 Spanish speaking adult patients (n=184) divided between intervention (n=91) and control (n=93).  5 Chronic diseases  6 Community health centre  7 Staff nurses | 8. Self rated health scores: no significant difference between pretest to posttest by study group F(2, 156)=0.13, p=.72 Mean scores decreased from pretest to post test in control group (M=3.79, SD=0.82 vs M=3.55, SD= 0.94), and intervention group (M=3.68, SD 0.86 vs M=3.38, SD= 0.86)  Self efficacy: not statistically significant from pretest to posttest by study group, F(2, 156)= 1.88, p=.17. Self efficacy of usual care group changed from M=5.89, SD=2.41 , to M=6.05, SD=2.69. Self efficacy of interention group changed from M=6.62 SD=2.41, to M=7.29 SD=1.94).  9 NR | 10 Intervention required no extra money or staff. Intervention was implemented into practices of the community health centre easily.  11 NR  12 Social desirability may have been a factor in patients’ reporting of whether they reached their goal. | 13  Self efficacy appears to be related to ability to manage chronic disease.  Although not statistically significant, the intervention group did demonstrate a a larger increase in self efficacy than the usual care group did.  14 Likert scales presented difficulties for Latino patients with low levels of literacy - verbal responses or other methods may be more appropriate in future research with this patient group.  Ongoing follow up, a reminder phone call and greater encouragement may help patients stay motivated , when implementing this type of intervention in future.  Assessment of system constraints e.g. Waiting time, may help improve this patient group’s engagement and retention in such interventions.  Future studies should examine resource availability, culturally appropriate methods of delivering interventions, and translation of materials. |

# Transitional Care

| **Source and type of evidence**  **Country**  **Aim** | **1. Definitions**  **2. Overview of Models**  **3. Main components** | **4. Population Group and Size**  **5. Health Condition / Problem**  **6. Healthcare context / setting addressed**  **7. Nursing disciplines involved** | **8. Outcomes assessed and effects on outcomes**  **9. Components associated with improved outcomes** | **10. Resource Implications**  **11. Enablers**  **12. Barriers** | **13. Key conclusions**  **14. Recommendations for Practice, Policy, Education, Research** |
| --- | --- | --- | --- | --- | --- |
| Parsons et al (2012)  New Zealand.  Meta-analysis of 3 RCT’s  To establish the impact of intermediate care on institutional free survival in frail older people referred for needs assessment in New Zealand (NZ). | 1. NR.  2. Intermediate care initiatives X3.  3. Intervention A: a home care service.  B. Slow stream rehabilitation service delivered within a residential facility.  C. Team of health professional care managers working at an advanced level of practice, located in the community | 4. Older people >65 (n=348).  5. Older people assessed at risk of institutionalization.  6. Community & residential care.  7. Team of health professionals: the regional geriatric assessment service and/or hospital clinical team. | 8. Death or permanent residential care placement.  ADL, IADL, Cognitive performance, Depression.  9. The combined primary outcome of institutional-free survival is 31% lower (95% CI: 9, 47) for the care-managed service models (combined) compared with usual care  Only the IADL involvement scale demonstrated a significant treatment effect for the intervention group at 0.31 (95% CI: 0.04–0.58) points lower than usual care. | 10. NR.  11. Key staff work in defined geographical boundaries. A strong care management focus. A well-coordinated home-based package of care Services coordinated and configured appropriately.  12. NR. | 13. The three intermediate care models, each following a strong care management approach designed to facilitate independent living, tested in a pre-planned meta-analysis, increased institutional-free survival.  14. When well-developed intermediate care services are utilized, public concern around the potential risks faced by older people when remaining at home does not appear to be supported |
| **Nurse as Coordinator** | | | | | |
| Feltner et al. (2014).  America.  Systematic Review and Meta-Analysis (n=47 RCTs).  *To assess the efficacy, comparative effectiveness and harms of transitional care interventions to reduce readmission and mortality rates for adults hospitalized with heart failure.* | 1. Transitional care interventions: Designed to prevent readmissions among populations transitioning between care settings.  2. Aim to avoid poor outcomes caused by uncoordinated care.  3. Categorised and defined by (a) Home Visiting Programmes; (b) Structured Telephone Support; (c) Telemonitoring; (d) Outpatient clinic based; (e) Primarily educational or (f) Other (e.g. Peer Support). | 4. Adults (18+ years).  5. Heart Failure (Mainly moderate – severe).  6. Recruited during or after inpatient admission.  7. Home or OPD clinic depending on intervention. | 8 & 9. Home visiting programmes and Multidisciplinary Heart Failure clinics reduced all-cause readmission and mortality (low SOE) at 30 days. Over 3 to 6 months, home-visiting programs and multidisciplinary heart failure (MDS-HF) clinic interventions reduced all-cause readmission (high SOE). Home-visiting programs reduced HF-specific readmission (moderate SOE). Structured telephone support (STS) interventions reduced HF-specific readmission (high SOE) but not all-cause readmissions (moderate SOE). Home-visiting programs, MDS-HF clinics, and STS interventions produced a mortality benefit.  Neither telemonitoring nor primarily educational interventions reduced readmission or mortality rates. | 10. NR.  11. NR.  12. NR. | 13. Home-visiting programs and MDS-HF clinic interventions currently have the best evidence for reducing all cause readmissions and mortality up to 6 months after an index hospitalization for persons with HF. These interventions should receive the greatest consideration by systems or providers seeking to implement transitional care interventions for persons with HF.  14. Future studies should evaluate  whether interventions that reduce readmission rates over 3 to 6 months also reduce 30-day readmission rates and could directly compare 1 intervention with another e.g. home-visiting program vs. multidisciplinary clinic). Given that many patients do not have access to specialty care (e.g. in rural settings) or may prefer care based in primary care clinics, future studies should evaluate the efficacy of transitional care  interventions in primary care clinics. |

| **Assisted Discharge** | | | | | |
| --- | --- | --- | --- | --- | --- |
| Utens et al. (2013).  Netherlands  RCT  Investigates preference and satisfaction in hospital-at-home & usual care. | 1 NR.  2 NR.  3 Hospital-at-home involves early discharge from hospital at day 4 of admission, + 1 home nurse visit on that day & for next 3 days + telephone access to hospital ward to day 7. | 4 COPD n=139, 69 UC, 70 HaH.  5.COPD.  6 Hospital care for 7 days followed by UC or discharge day 4 and follow up nurse care at home until day 7 with phone access to hosp.  7 Comm Nurse. | 8 Regarding patient satisfaction 49% (n=34)UC and 41% (29) HaH a score was obtained thus from those numbers, 70% were satisfied in UC group & 71% HaH group. Only significant difference was the group at home felt more unsafe at night than UC group (p<0.029) and less ability to resume activities (p, 0.018).  9 Negative comments mostly related to medical care, around medication. Intervention group were very satisfied to be at home as environment more familiar, clean, quiet, provided privacy, and ability have own regular routines. | 10. NR.  11. NR  12. Visiting arrangements for home visits agreed and arranged prior to discharge; expectations of and content of home care visits need to be made explicit. | 13 Preference for home treatment from both groups 42% (UC) and 86% HaH group.  14. Patient preference should be considered an option for these patients given the similar outcomes fro hospital and home care health outcomes and satisfaction is greater with home care. |
| 9. Utens et al. (2015).  Netherlands.  RCT-further reporting on RCT by Utens et al (2013)  To determine the effectiveness of early  assisted discharge for chronic obstructive pulmonary disease (COPD) exacerbations, with home care provided by generic community nurses, compared with usual hospital care. | 1. NR.  2. NR.  3 Hospital-at-home involves early discharge from hospital at day 4 of admission, + 1 home nurse visit on that day & for next 3 days + telephone access to hospital ward to day 7. | 4. COPD n=139, 69 UC, 70 HaH.  5. COPD.  6. Hospital care for 7 days followed by UC or discharge day 4 and follow up nurse care at home until day 7 with phone access to hosp.  7. Comm generic Nurses | 8. No difference between groups was found in change in CCQ score at day 7 (difference in mean change 0.29 (95% CI −0.03 to 0.61)) or at 3 months (difference in mean change 0.04 (95% CI –0.40 to 0.49)). No difference was found in secondary outcomes. At day 7 there was a significant difference in change in generic HRQL, favouring usual hospital care.  9. NR. | 10. NR.  11. NR.  12 Barriers refer to the challenges researcher met in trying to recruit and maintain sample; no blinding possible, treatment at home is not allowed if patients require nebuilsers or oxygen if this was not part of their prior tx prior to admission. This extension of sending patients with higher levels of care would reqire community nurses to have more specialist training. | 13 ‘Early assisted discharge with home visits by  community nurses is a feasible and an alternative to usual hospital care for selected patients with an acute exacerbation of their COPD’ (p. 7)  14. This intervention needs to be further investigated as there are no differences of outcomes for these patients and if it is patient preference should be facilitated. |

| **Outreach Nursing** | | | | | |
| --- | --- | --- | --- | --- | --- |
| Blair et al. (2011).  Scotland.  Systematic review  To examine the evidence for home versus hospital cardiac rehabilitation | 1. Inconsistency in terms of what was being provided as ‘home’ or ‘community based’ cardiac rehabilitation (CR).  2. Home’ or ‘community based’ cardiac rehabilitation.  3. Nurse led models had a  variety to components e.g. home visits, phone calls, education, support, coordination. | 4. 17 studies met the inclusion criteria. Not possible to combine studies but sample sizes ranged from 30 to 1376 adults.  5. Cardiac patients.  6. In a patients’ home or in a local, non-hospital location.  7. There were six studies in which nurses provided the intervention | 8. Outcomes of SR related to home based CR:  - A relative reduction in mortality of approx. 25%.  - a reduction in re-hospitalisation  - a greater reduction in serum cholesterol  - A significant reduction in angina frequency  - little or no difference in physical activity outcomes  - Reduction in anxiety and depression.  9. Nursing component not extracted or described. | 10. There was no significant difference in healthcare costs between the two patient groups over a 9 month period.  11. NR.  12. For patients living in remote and rural areas, the most prominent barriers are accessibility and distance. | 13 There is no consistent difference in outcomes between home and hospital-based CR.  14. More work is needed on the long-term safety and effectiveness of different modes of home service delivery. This includes home CR and also for more modern approaches such as 'tele-rehab' |
| Fischer et al. (2012).  USA.  RCT.  The focus of this study was to determine whether algorithm-driven  telephone care by nurses improves lipid  control in patients with diabetes attending a federally funded community health centre in the USA. | 1. NR.  2. Nurse led outreach programme of care.  3. Community nursing using telephone based care to target lipid control in diabetic patients. | 4. Adults with diabetes (n=762).  5. Diabetes- lipid control.  6. Community Family Health Centre.  7. Community Nurses. | 8. Primary outcome: Proportion of patients with a low-density lipoprotein (LDL) less than 100 mg/dL following the intervention. Secondary outcomes: numbers of hospital admissions, hospital charges per patient,  the proportion of patients meeting other lipid, glycemic, and blood pressure guideline.  The percentage of patients achieving the LDL goal of less than 100 mg/dL increased in the intervention group and decreased in the usual-care group.  The intervention group performed significantly better than the usual-care group on our primary outcome, the percent of patients with an LDL less than 100 mg/dL in the preceding year (increased from 52.0% to 58.5% vs decreased from 55.6% to 46.7%, P <.01).  The percent of patients with an LDL <100 mg/dL increased from 52.0% to 58.5% in the intervention group and decreased from 55.6% to 46.7% in the control group (P <.01). Intervention patients trended toward fewer hospital admissions (P = .06). The intervention did not affect glycemic and blood pressure outcomes.  9. An “on-intervention” analysis compared lipid outcomes for the control group versus intervention patients with 3 or more contacts by the nurses during the study period. A higher percentage of the patients in this intervention subgroup were at goal at study end for the primary lipid outcome of LDL less than 100 mg/dL (69.1% vs 46.7%, P <.01). Among those patients with cardiovascular disease, only the “on-intervention” patients achieved the goal of LDL less than 70 mg/dL more than the control  patients (50.0% vs 30.4%, P = .02). The intervention did not impact glycemic and blood pressure outcomes. | 10. Average cost per patient to the healthcare system was less for the intervention group ($6600 vs $9033, P = .03). Telephone-based outreach may decrease resource utilization especially inpatient and emergency department usage.  11. NR.  12. NR. | 13. Nurse-led, telephone-based case management program served a vulnerable, underinsured population and was associated with improved lipid control and a decrease in overall healthcare utilization.  14. Nurses can improve lipid control in patients with diabetes in a primarily indigent population  through telephone care using moderately complex algorithms, but a more targeted  approach is warranted.  Telephone-based outreach may decrease resource utilization, but more study is needed. |
| Stewart et al. (2014B).  Australia.  RCT.  This study compared the longer-term impact of the two most commonly applied forms of post-discharge  Management, outpatient hospital clinic care and home based management, designed to minimize recurrent hospitalization and prolong survival in typically older patients with chronic heart failure (CHF). | 1. NR.  2. Focused on forms of post-discharge management designed to minimize recurrent hospitalization and prolong survival in typically older patients with chronic heart failure (CHF).  3. Patients allocated to home-based or specialized CHF clinic-based intervention. | 4. Older adults (n=280)  5. Older patients with CHF.  6. Hospital Clinic and Home Setting.  7. CHF nurse specialist  home intervention managed via an out-reach program of home visits with multidisciplinary team. | 8. No difference was found in the primary endpoint; 20 (14.0%) home-based versus  13 (7.4%) clinic-based patients remained event-free (adjusted HR 0.89, 95% CI 0.70 to 1.15; p= 0.378).  Significantly fewer home-based (51/143, 35.7%) than clinic-based intervention (71/137, 51.8%) patients died (adjusted HR 0.62, 95% CI 0.42 to 0.90: p = 0.012).  Hospitalisation lengths indicated- Home-based versus clinic-based intervention patients accumulated 592 and 547 all-cause hospitalizations (p = 0.087) associated with 3067 (median 4.0, IQR 2.0 to 6.8) versus 4410 (6.0, IQR 3.0 to 12.0) days of hospital stay (p b 0.01 for rate and duration of hospital stay).  9. NR. | 10. NR  11. Favorable outcomes in the home-based intervention group are consistent with some of the observed benefits of home visits to develop a more therapeutic relationship with older  individuals to empower them to feel more in control and proactively manage their condition(s); focusing less on the syndrome of CHF and more on the affected individual overall.  12. NR. | 13. A home based management approach to the post-discharge management of typically older patients with CHF provides a potentially cost-effective (pending a formal health economic analysis) means to both prolong CHF-related survival and limit recurrent hospital stay. Home-based intervention was, however, associated with significantly fewer all-cause deaths and significantly fewer days of hospital stay in the longer-term but not associated with prolonged event-free survival.  14. Urgent need to undertake more head-to-head trials to determine the most cost-effective form(s) of CHF management. |
| Stewart et al. (2012).  Australia.  RCT - further reporting on RCT by Stewart et al (2012).  Comparison of home based intervention and a specialised clinic based intervention for people with CHF. Hypothesised that a home-based approach would be more effective in optimizing health outcomes due to a better overall understanding of the patient and their environment. | 1. NR.  2. CHF nurse specialist  home intervention  managed via an out-reach program of home visits  Nurse led chronic heart failure management  programs [CHF-MPs] in specialist hospital clinic  3. NR. | 4.Older adults (n=280)  5. Older Patients hospitalized with CHF.  6. Specialist Hospital Clinic and Home Setting.  7. Nurse led chronic heart failure management  programs [CHF-MPs] in hospital clinic with multidisciplinary team  CHF nurse specialist  home intervention managed via an out-reach program of home visits with multidisciplinary team | 8. Minimal difference with respect to the primary endpoint of (all-cause) death or unplanned readmission  during 12 to 18 months of follow-up.  HBI had a better survival profile (6% absolute difference), this did not reach statistical significance.  HBI patients accumulated significantly fewer days of total all-cause (35% days fewer) and cardiovascular-related hospitalization (37% days fewer), with a consistent (borderline) trend with respect to less unplanned hospitalization (30% days  fewer).  HBI was also associated with a non significant reduction in CHF stay.  9. NR. | 10. Reduced cost of care with Home Based Intervention. Significantly more prolonged days out of hospital alive in favor of HBI reflect decreased total healthcare costs. Costs were nearly one-third less within the HBI group  11. Study highlights patients prefer and respond better to a health intervention that appears to be more flexible to their needs.  12. NR | 13. The combination of greater uptake of HBI overall, a more favourable profile free from hospitalization and death, and more favorable cost dynamics (largely mediated through a  reater reduction in non-CHF–related hospitalization) are of clinical and public health significance.  HBI facilitates individualised care for a cohort of patients with complex needs.  14. NR |
| Verhaegh et al. (2014).  Netherlands.  Systematic Review & meta-analysis (n= 26 RCTs)  Examine if transitional care interventions associated with reduction of readmission rates in short (30 days or <), intermediate (31-180) and long term (181-365 days). | 1. Transitional Care Model  (Naylor et al 2011 Health Affairs).  2. A bundle of discharge interventions.  3. 26 studies reviews. :54% included comp t assessment at admission, 81% self manage education during admission, 54% involved caregiver as secondary recipients of intervention. 69% had care coordination by nurse. | 4. 7932 (3992 in control grps) age 18 or > (p 1536)  5. Variety of conditions ex: heart failure, COPD, asthma, general surgery  6. “Hospital to home” continuum setting.  7. NR. | 8. Transitional care associated with risk reduction of 5% in intermediate-term readmissions  (OR: 0.77; 95% CI: 0.62, 0.96) and 13% in long-term readmissions (OR: 0.58, 95% CI:0.46, 0.75) (Exhibit 1–3; not effective in reducing short-term readmissions (OR:0.76; 95% CI: 0.52, 1.10).  High-intensity interventions  associated with reduced short-term readmissions  (OR: 0.59; 95% CI: 0.38, 0.92), intermediate term (OR: 0.69; 95% CI: 0.51 0.92), and longterm readmissions (OR: 0.57; 95% CI: 0.35,0.92)  Low-intensity interventions sig. associated with reduced long-term readmission (OR: 0.62; 95% CI: 0.46, 0.82).  Transitional care associated with a 5% lower rate of intermediate-term readmission(OR: 0.74; 95% CI: 0.59, 0.93) and 8%lower rate of long-term readmission (OR:0.71; 95% CI: 0.56, 0.91) in patients > 60 yrs.  No evidence transitional care associated w/ reduction short-term readmissions.  Care coordination by a nurse (OR: 0.60; 95% CI: 0.37, 0.98; p ¼ 0:04), communication between the hospital and the primary care provider (OR: 0.33; 95% CI: 0.12, 0.87;p ¼ 0:03), and a home visit within three days of discharge (OR: 0.44; 95% CI: 0.26, 0.76; p < 0:001) significantly associated with reduced rates of short-term readmission.  9. Home visit within 3 days, care coordination by a nurse (nurse or ANP), communication between hospital and primary care provider components of transitional care significantly associated with reduced short-term readmission rates. | 10. Different approaches may be needed to prevent readmits in short, intermediate, and long terms.  To reduce hosp readmission, high intensity interventions might be needed such as home visit within 3 days.  Interventions for effect on intermediate and long term readmit lasted >30 days.  11. NR.  12. NR. | 13. Transitional care effective in reducing all-cause intermediate-term and long-term readmissions. Only high-intensity interventions effective in reducing short-term readmissions. To reduce short-term readmissions, transitional  care should consist of high-intensity interventions that include care coordination by a nurse, communication between the primary care provider and the hospital, and a home visit within three days after discharge. Transitional care effective for pts >60 yrs. Ideal content of transitional care programs and target gps are still unclear.  14. Transitional interventions important where countries have a target to reduce 30 day readmissions. |

| Wong et al. (2011).  Cochrane SR (N= 9 RCTs).  Australia.  *“To evaluate the effectiveness of outreach respiratory health care worker programmes for COPD patients in terms of improving lung function, exercise tolerance and health related quality of life (HRQL) of patient and carer, and reducing mortality and medical service utilisation.’* (p.1) | 1. NR.  2. NR.  3. Outreach nurse visiting  patients homes, providing support, education, monitoring health and liaising with physicians. | 4. 9 RCTs with 1498 patients with moderate-severe COPD.  5. COPD.  6. home visits by a respiratory nurse or respiratory health worker, to facilitate health care, provide education, provide social support, identify respiratory deteriorations promptly and reinforce correct technique with inhaler therapy. Eligible control groups were patients who received routine care, without respiratory nurse/health worker input.  7. NR. | 8. 8 studies found a non-sig. reduction in mortality at 12 months (OR 0.72, 95% CI 0.45 to, 1.15). 4 studies that assessed disease-specific heath-related quality of life (HRQL) found a sig. improvement in HRQL (mean difference -2.61, 95% CI -4.82 to -0.40). 5 studies found no statistically significant difference in the number of hospitalisations  (OR 1.01, 95% CI 0.71 to 1.44), there was significant heterogeneity.  9. NR. | 10. One study assessed cost, indicated that home care was an expensive form of care.  11. NR.  12. NR. | 13. Home care resulted in an improvement in people’s quality of life, but has an unpredictable effect on the risk of being admitted to hospital.  15. More research needed to confirm usefulness of home visits for COPD regarding costs. |
| --- | --- | --- | --- | --- | --- |
| **Care Planning** | | | | | |
| Wong et al. (2014).  China.  RCT.  *To explore effects of transitional care programme among group of discharged patients with chronic diseases.* | 1. Transitional care model of care  2. Pre hospital discharge (d/c) assessment (Omaha system); post discharge application of Omaha system to assess, intervene and implement transitional care programme constructed with same design for home and call groups. Post d/c follow up two home visits week 1 and 3, two phone calls week 2 and 4 .  3. 3 groups – intervention group two arms: home visits w/ tele call, other tele call only. Control gp usual care – basic health advise, med instructions and arrangements for out pt follow up. detail lacking on the actual components of the calls and home visits. | 4. Adults (n=610).  5. Discharged with various conditions.  6. Post hospital discharge follow up in community.  7. Nurse case manager (NCM), assisted by trained nursing students. | 8 Service outcomes: home visit (HV) group significantly lower readmission (17.6%) OR=0.541, p=0.041 rate than control (17%) but no difference in call group ((11.8%, OR = 0.624, P = 0.102 at 4 weeks.  Self efficacy among groups over time improved (F=6.15, P=0.002) but interaction effect between group and time not significant. Intervention groups significantly higher satisfaction scores than control ((F=76.99, p<0.001)).  9. Combination of home visit and call had a significant effect. | 10. Use of nursing students to support Nurse Case Manager allows NCM to focus on work requiring skill set of RN (assessment, prescription of intervention plan and case management).  11. NR.  12. NR. | 13. Affirmed the effects of transitional discharge care.  Telephone calls alone may not be sufficient to bring about significant reductions in readmissions. Bundled interventions of home visits and calls are more beneficial for patients after discharge,  14. In systems facing resource constraints skill mix using support workers to assist qualified health professionals is advocated. |

# Telehealth

| **Source and type of evidence**  **Country**  **Aim** | **1. Definitions**  **2. Overview of Models**  **3. Main components** | **4. Population Group and Size**  **5. Health Condition / Problem**  **6. Healthcare context / setting addressed**  **7. Nursing disciplines involved** | **8. Outcomes assessed and effects on outcomes**  **9. Components associated with improved outcomes** | **10. Resource Implications**  **11. Enablers**  **12. Barriers** | **13. Key conclusions**  **14. Recommendations for Practice, Policy, Education, Research** |
| --- | --- | --- | --- | --- | --- |
| **Telephone support** | | | | | |
| Fischer et al (2015).  US.  RCT.  To evaluate the ability of an additional nurse directed telephone intervention to reduce primary medication non-adherence. | 1. NR.  2. Nurse reminder to patient to pick up medicines not collected at the pharmacy.  3. Up to three attempts over 2 days to call the patient on the phone at different times of day. Message left on patient’s phone if not contacted. | 4. Patients identified as PMN (primary medication non-adherent) i.e. who had not collected their newly prescribed medicines after 14 days and three automated and one live call from the pharmacy (n=290).  5. Patients who were on medicines for hypertension, hyperlipidaemia, asthma and type 2 diabetes.  6. Geisinger Health System – network of over 40 community based clinics in Pennsylvania.  7. Nurses working with the patient’s healthcare provider who prescribed the eligible medication. | 8. Primary outcome was pick up of prescription within 30 days. 25% of intervention patients and 24% of control patients picked up their prescriptions within 30 days. Difference non significant Multivariate analysis OR 1.08 (95% CI 0.62-1.89, p=0.79).  9. Antihypertensives showed the highest rate of pick up (47%), hyperlipidaeamia meds lowest (5.3%). | 10. NR  11. NR.  12. Delay before contact by nurses initiated was identified as a possible factor in apparent ineffectiveness of intervention. Low level of primary medication nonadherence (6% - much lower than found in previous studies) was also a factor in the lack of effectiveness of the intervention | 13. Nurse reminder calls for patients who were primary medication non-adherent for newly prescribed medicines for certain chronic diseases did not prove to be effective.  14. More research needed on current rate of primary medication non-adherence (which may have changed from when first researched). Intervention may need to be changed to include re-engagement with the primary care provider who prescribed the medicine. |
| Fortney et al. (2013).  USA  RCT. Pragmatic trial (n=364)  ‘To compare the process outcomes and clinical outcomes of patients randomized to Telemedicine Based Collaborative Care (TBCC) TBCC versus *Practice Based Collaborative Care (PBC)C* in small remote primary care clinics lacking on-site mental specialists’, (Pg 3) | 1 NR  2. Intervention  Telemedicine Based Collaborative Care (TBCC) an off-site team of MH specialists collaborating with on-site PC providers, using telephones, interactive video and electronic health records.  Control received  Practice Based Collaborative Care (PBCC)  3. Telemedicine-Based Collaborative Care (TBCC)  five types of providers: on-site PC providers, and off-site DCM (RN), pharmacist (PharmD), psychologist (PhD) and psychiatrist (MD). All interactions were delivered by telephone and via interactive video, following a protocol .There were weekly team meeting who made treatment, recommendations of treatment were delivered via the DCM . Patients not responding to treatment were reviewed by a telephone pharmacist who identified medication changes as needed. A psychiatry consultation was also available via interactive accessed cognitive behavioral therapy (CBT) via interactive video, | 4&5  Patients (n=364) with depression screened using the PHQ9  5. Depression  6 5 Federally Qualified Health Centers in areas with poor medical services . Small remote PC clinics associated with the largest publically-funded healthcare system in the country  7 Registered nurses with no Mental health background | 8  Blinded telephone interview using following instruments Depression Outcomes Module, Mini International Neuropsychiatric Interview, Duke Social Support and Stress Scale, Quality Improvement for Depression Treatment Acceptability scale, and the Depression Health Beliefs Inventory  MH service utilization, (Hopkins Symptom Checklist SCL-20), treatment response and remission.  9 Compared to patients randomized to PBCC, TBCC group had significantly and substantially greater treatment response rates, remission rates, reductions in depression severity and increases in mental health status and quality of life. Improved outcomes were achieved in the TBCC group without increasing the number of PC visits.  ‘The greater fidelity to the care manager protocol did not translate into improvements in the quality of pharmacotherapy in the TBCC group. The majority of patients in both groups initiated antidepressant treatment at therapeutic dosages and adherence was high. Likewise, the psychotherapy available to patients in the TBCC group via interactive video was not highly utilized, and was not likely to have contributed substantially to improved outcomes’. (Pg 7) | 10NR  11 ‘Improved outcomes appear to be due to higher fidelity to the care manager protocol in the TBCC model, despite the fact that off-site and on-site DCMs had similar levels of clinical experience at baseline and underwent identical training’. (Pg 8)  12 a high degree of treatment resistance may have contributed to low response/ remission rates among the PBCC group | 13 & 14 results clearly indicate that contracting with an off-site depression care team yields better depression outcomes than implementing collaborative care with staff available on-site.  ‘Future research should examine whether having on-site nurse care managers supported by off-site tele-psychiatrists, tele-psychologists and tele-pharmacists is an effective organizational approach to delivering collaborative care.’( Pg 8) |
| Krum et al. (2013).  Australia.  RCT.    To test the effect of a telephone support strategy, as an addition to usual care (CHAT) UC+1, in reducing major events in rural and remote Australians with chronic health failure (HF) who have limited access to healthcare. | 1. NR.  2. Telephone support in disease management.  3. CHAT (UC+1) was Telephone support comprised an interactive Telecommunication software tool (TeleWatch) with follow-up to 12 months by trained cardiac nurses. | 4. Adult Patients (n=405).  5. Chronic heart failure.  6. Primary care - managed in general practice.  7. Nurses with specialist Cardiovascular experience. | 8. The primary endpoint was the Packer clinical composite score (relating to 1. Death. 2. Hospital admission for HF. 3 worsening heart failure. 4. Seven-point overall well-being). Found no difference between the two groups (P = 0.98).  In terms of secondary endpoints there were fewer patients hospitalized for any cause (74 vs. 114, adjusted HR 0.67 [95% CI  0.50–0.89], P = 0.006) and who died or were hospitalized (89 vs. 124, adjusted HR 0.70  [95% CI 0.53–0.92], P = 0.011), in the UC+I vs. UC group.  9. NR. | 10. Patients in the UC group visited their general practitioner more frequently compared with those in UC + I (12.55 GP visits/patient [UC] vs. 5.85 GP visits/patient [UC + I]). Overall, more than 65% of patients in the intervention made an average of 24 unscheduled calls/patient to the TeleWatchTM system.  11. NR.  12 Participants were required to possess and use a keypad phone at least on a monthly basis. | 13 The intervention significantly reduced the number of HF patients hospitalized in the rural and remote groups.  14. Based on the beneficial impact on hospitalization in patients with chronic HF, automated telephone support should be considered for other chronic disease requiring ongoing management and multidisciplinary approaches for patients living in rural and remote regions. These diseases may include chronic arthritis, COPD, diabetes mellitus, and osteoporosis.  Findings support further research to analyse the cost-effectiveness and feasibility with other chronic disease states. |
| Mohammadi et al. (2013).  Iran.  RCT  The aim of the study was to assess the effect of home-based nursing pulmonary rehabilitation on fatigue, ADL and QOL in patients referred to a subspecialty training hospital | 1 NR.  2. The model is termed home based nursing pulmonary rehabilitation …however the only nursing discipline input is in-hospital training and follow up by a research nurses while the patient is home in the community.    3. The home-based rehabilitation programme consisted of 3 x 1hr sessions of face-to-face, individual training in the intervention group on education of COPD symptoms, correct methods of walking and pursed lip breathing and a pictorial booklet followed by practical sessions and follow-ups by nurses at the patients’ home by telephone. Control group usual care consisting of drug tx. Patients were telephone contacted daily by research nurse. | 4. Patients (n=106) at stage 2 or 3 COPD based on GOLD criteria (2009)  5. COPD.  *6.* Initial training in-hospital and follow up at home after 7 weeks.  7. In-hospital nurse training of patients in intervention and research nurses follow up with telephone calls. | 8. Home-based nursing pulmonary rehabilitation had a significant effect on fatigue (p<0.001); ADL (p<0.001). and QOL (p<0.001).  9. Nursing input was remote but deemed significant in motivating and providing feedback to patients. | 10. NR.  11. Willing and suitable patients.  12. Need for training of patients on the HM systems that are person-centred and followed up by nurses. | 13. Home-based nursing pulmonary rehabilitation *could reduce fatigue and improve ADL and QOL in patients with stage 2 or 3 COPD offering an alternative to institution based rehabilitation.*  14. Research suggests that although home-based  nursing rehabilitation has been shown to be effective, there is still a need to design an applied model in order to present it. It seems that home-based rehabilitation programmes should be moderated based on patients’ needs and contextual conditions. |
| Walters et al. (2013).  RCT.  Australia.  ‘*This study investigated the hypothesis that telephone-*  *delivered health mentoring by nurses in community-*  *recruited patients with stable moderate or severe COPD would increase self-management capacity and improve QOL and psychological well-being’ (p. 2).* | 1. NR.  2. The intervention was a telephone home monitoring service delivered by trained community health nurses as health mentors.  3. Intervention training of community nurses was underpinned by cognitive behaviour theory with ultimate goal of patient self-management using five core components: Psycho education; self-management skills; cognitive coping skills; communication skills; promoting self-efficacy. | 4. Adults aged 68 +/-8yrs (n=182). 62% moderate COPD, 53% men).  5. COPD + hx >10 yrs smoking; FEVI forced vital capacity ratio <0.7 and FEVI 30-80%.  6. At home in the community.  7. Community nurses trained in health mentoring. | 8. There was no difference in quality of life between groups, but self-management capacity increased in the HM group (PIH overall 0.15, 95% CI 0.03 to 0.29; knowledge domain 0.25, 95% CI 0.00 to 0.50). Anxiety decreased in both groups (HADS A 0.35; 95% CI −0.65 to −0.04) and coping capacity improved (PIH coping 0.15; 95% CI 0.04 to 0.26).  9. It is thought that the nursing component enhanced the findings relating to self-management and knowledge. However as the UC group also received calls but it is unknown who made those calls (/ research person or a qualified nurse) and they also had positive clinical outcomes e.g. self-management coping capacity. | 10. NR.  11. Patients willing to engage in telephone assessment and management. Nurses were trained in HM underpinned by theory and also focused on prevention and measured adherence to delivery of a behavioural intervention.  12. High withdrawal rates from HM group | 13. Health mentoring delivered by community health nurses increased the self-management capacity of people with COPD in the community but did not change QOL. A short empathetic phone call had some positive effects and should be further assessed.  14. Optimising training and supporting HMs skill development in routine community nursing and/ general practice will be an important area for future research. |
| **Telemonitoring** | | | | | |
| Cruz et al. (2014).  Portugal.  Systematic Review (N= 17 articles include 12 studies i.e. 5 RCT; 4 pre-posttest; 3 quasi).  To describe the methodologies used in home tele monitoring (HM) interventions for COPD and to explore patient’s adherence and satisfaction with use of tele monitoring systems | 1. Home tele monitoring facilitate self-care management of COPD. It is defined as ‘the use of telecommunication  technologies to transmit data on patients’ health status (e.g. oxygen saturation, vital signs) from home to a healthcare center’.  2. HM for COPD can include a number of physical symptom assessments which can be recorded electronically; uploaded onto data centre and assessed remotely by clinicians/nurses or electronically to determine any abnormalities or exacerbation of condition  3. NR. | 4 Patients with COPD.  5. COPD.  6. Patients using HM from home to a healthcare centre.  7. One study included an in-home nurse visit. Remainder of studies included nurses assessing clinical data at healthcare centre as member of clinical teams or ‘usual care’ (unclear definition). | 8. No clinical outcomes measured. Patients were generally satisfied and found HM useful to help them manage their disease, some difficulties is their use were related to lower compliance. Compliance was higher if data transmitted once a day and lower if more frequent,( 98% vs 79% for oxygen saturation and 83% vs 60% for Resp rate).  9. As nurse visit was included in only one study no nursing outcomes were reported | 10. NR.  11. Active role that nurses can have as members of the community-based rehabilitation team.  12. Need for HM systems to suit the patient and more patient training for HM. | 13 HM can be beneficial for COPD patients in supporting their self-management and knowledge of their condition. HM systems and methodologies need to be specific to needs of patients (touch screen, resp belts etc. may not suit all patients; poor dexterity).  14. Use of HM can be cost effective as opposed to hospital rehabilitation, however there is a need for more training for patients/person-centred systems/easy to use technology for people with disabilities/flexibility of data collection and transmission use of robust questionnaires to measure satisfaction. |
| Gellis et al. 2012/14  USA  A Randomized Controlled Trial (n=115)  **Aim** ‘examining the impact of a multifaceted telehealth intervention(Telehealth for Heart Education Activation Rehabilitation and Treatment ( teleHEART ) on health, mental health, and service utilization outcomes among homebound medically ill older adults diagnosed with HF or COPD’ (Pg 541) receiving home health skilled nursing care | 1. NR  2 . Builds on the model of an empowering- person centered care approach  3 Intervention “ HomMed ” Health Monitoring System consisting of home monitor unit which provides Audio prompts instructed and transmits patient data to to a Central home health care agency. Daily monitoring of weight, noninvasive blood pressure, pulse, oxygen saturation, and temperature at preset scheduled time guided by patient preference. Text prompts present on monitor cueing patients until the task completed. Patient’s data reviewed daily by telehealth Nurse who contact Patients having abnormal readings for further evaluation and where necessary referred to wider Community care team .  *Usual Care* Participants assigned to the usual care plus education control group received standard home care services provided by registered nurses in the role of case managers | 4.  Adults 65 years or older, (n=115). Homebound,  frequent health care encounters  Willing to learn how to use system  5. Heart Failure or COPD  6. Large hospital-based Medicare-certified home care programs. Participants recruited from hospital discharge planners physicians, surgeons, and community health centers  7. Homecare Nurse | 8. Depression Center for Epidemiologic Studies Depression (CES-D) Scale and the Patient Health Questionnaire (PHQ).  Medical Outcomes SF-36.—  Patient Satisfaction Survey  9. 89% of patients (51/57) randomized to the telethealth services.  At 3 months, depression improved significantly in the tele-HEART intervention group PHQ-9 ( F = 6.47, p < .008) and the CES-D (7.81, p < .004)  Telehealth patients reported more interest in daily activities, less sadness, and increased energy than controls. They also  experienced significantly higher increases in two of SF-36 scales: general health ( F = 3.91, p < .016) and social functioning ( F = 3.64, p < .014) but not bodily pain.  Both groups were generally satisfied with their care with no observed significant differences.  Control group had significantly more visits to the emergency department.  The telehealth group had fewer hospital days but was not significant at 12 months. No significant group differences in number of home health care visits over 12 - months . | 10. NR  11. These positive outcomes for intervention patients a reduction in the number of emergency department visits and a trend, toward a reduction in days in hospital and home visits needed during hospital readmissions.  11 With assistance from the telehealth nurse, were empowered the client to become more active in ther health monitoring and management  ‘an empowering-centered care approach ,where the patient is coached to fi nd their strengths and be self-directed in problem solving and management of the chronic illness with a telehealth nurse as a coach’(pg 547).  12 What constitutes Tele-HEART intervention requires further clarity as it may be difficult to work out which components have the effects on the study outcomes | 13. ‘Telehealth may be an efficient and effective method of systematically delivering integrated care in the home health sector. The use of telehealth technology may benefit homebound older adults who have difficulty accessing care due to disability, transportation, or isolation  Findings from this randomized study may be generalizable to other Medicare-certified home health care agencies public or private because they all utilize a uniform centralized system to support the delivery of home-based nursing skilled care’ (Pg 550).  14 A deconstruction study was recommended.  Further investigation into understand the variable ant their relationship to patient motivation for behavioral change.  an economic analysis would be important |
| Konstam et al. (2011).  USA.  RCT.  To examine the additive value of automated home monitoring (AHM) to a previously described nurse-directed Heart Failure (HF) management program (SPAN-CHF), with attention to Health related QOL. | 1. NR.  2. Automated home monitoring.  3. SPAN-CHF =Specialised Primary and Networked Care in Heart Failure containing a) specialized nurse managers, b) pharmacologic guidelines; c) visit by nurse and/or telephonic weight and symptom monitoring; and 4) compliance education  AHM is a technological component designed to convey weight and vital signs information and a text message component addressing symptoms, functional status, and medical regimen compliance. | 4. Adult patients (n=188).  5. Recent history of decompensated HF.  6. Home.  7. Specialised nurse managers. | 8. SPAN-CHF with and without AHM improved HRQL at 45 and 90 days compared with baseline with respect to Physical, Emotional, and Total domain scales but no significant difference emerged between with and without AHM groups.  9. Researchers acknowledge that it is not possible of assess the relative contribution of each component | 10. NR.  11. NR.  12. NR. | 13 The study did not support the added benefit of a technological intervention over a non-technological intervention.  14. AHM has the potential to allow for greater nursing workloads which should be further researched. |

| Steventon et al (2013)  UK  Cluster Randomised trial (n=2426)  Telecare was compared with ‘usual care’ | 1. NR   2. Telecare using functional monitoring  3’Functional monitoring, including the ‘Lifeline’ base units and pendants, bed and chair occupancy sensors, enuresis sensors, epilepsy sensors, fall detectors and medication dispensers. • Security monitoring, including bogus caller buttons, infrared movement sensors and property exit sensors. • Environmental monitoring, including gas, monoxide and smoke detectors, heat sensors, temperature extremes sensors and flood detectors  • Standalone devices not linked to a monitoring centre, such as big button phones, key safes for careers and memo minders.  Data from the peripheral devices were sent to a monitoring centre via a telephone line and alerts were monitored continuously’ (Pg 502).  Compared to usual care | 4. People with social care needs, aged over 18  5 people with social care needs e.g. minimum level of social care service , mobility difficulties; At risk of falling;  cognitive impairment  6. All general practices in Cornwall, Kent and Newham with 238 practices allocated to control or intervention groups, of which 217 ultimately supplied participants for the trial  7 practice nurse | 8. Followed up for 12 months  proportion admitted to care  Secondary endpoints included: mortality Health care utilization: number of weeks receiving domiciliary care , inpatient hospital bed days, emergency admissions, elective admissions, admissions for falls, outpatient attendances and accident and emergency visits.  9 Of intervention group 46.8% were admitted to hospital compared with 49.2% of controls. This difference was not statistically significant There were also no significant differences in the number of weeks receiving domiciliary social care between groups General practitioner contacts were significantly higher among intervention than controls (incidence rate ratio: 1.18, 95% CI: 1.01–1.38, P = 0.033), There were no significant differences between groups in the cost associated with hospital care and social care; Mortality rates were not significantly different between groups. There were no significant differences in lengths of hospital stays | 10. NR  11 Decision-making should wait until results relation to the quality of life, carer outcomes and experience are available  12NR | 13. Telecare as implemented in the Whole Systems Demonstrator trial did not indicate any significant reductions in service use assessed over 12 months  14. No major recommendations |
| --- | --- | --- | --- | --- | --- |
| Vuorinen et al (2014)  Finland  Randomized controlled trial (two-arm Stepwise  Matched pair design) (n=94)  **objective ‘**to investigate whether the multidisciplinary care of Heart failure patients could be improved with telemonitoring at the Cardiology Outpatient Clinic of Helsinki University Central Hospital (HUCH), primarily in terms of reducing HF-related hospitalizations’.(pg2) | 1. NR  2. A Telemonitoring-Assisted Self-Care Model  3. Intervention  patient regularly reported relevant health parameters to the nurse using a mobile phone app  Patient provide home care pack which including:  Weighing scale, BP Monitor a mobile phone with a pre-installed software app  Self-care instructions which advise when to carry out and report the results together with the assessment of symptoms once a week.  On submission of results, they receive automatic machine-based feedback if parameter are within the Individualized targets set by the nurse.  The nurse reviews status and weekly or more frequently if necessary  **Usual Care**  multidisciplinary care approach including patient guidance and support for self-care . | 4. 18-90 year old heart failure  5 Heart failure (HF) patients  whose left ventricular ejection fraction was lower than 35%, NYHA functional class ≥2, requiring regular follow-up.  6. Outpatient Clinic of Helsinki University Central Hospital (HUCH),  7. Heart failure nurse | 8. Follow-up at 6 months  The primary outcome was number of HF-related hospital days  Secondary outcomes: Use of health care resources, (medication. ED and Outpatient visits, nursing & physian time), user experience  Self-care behavior  (European Heart Failure Self-Care Behaviour Scale)  9. No difference was found in the number of HF-related hospital days (incidence rate ratio [IRR]=0.812, P=.351), which was the primary outcome. The intervention group used more health care resources: they paid an increased number of visits to the nurse (IRR=1.73, P<.001  spent more time at the nurse reception (mean difference of 48.7 minutes, P<.001  and there was a greater number of telephone contacts between the nurse and intervention patients (IRR=3.82, P<.001  for nurse-induced contacts and IRR=1.63, P=.049 for patient-induced contacts). There were no statistically significant differences in patients’ clinical health status or in their self-care behavior. The technology received excellent feedback from the patient and professional side with a high adherence rate throughout the study  telemonitoring-assisted care approach led to increased use of health care resources while showing no quantified improvement in the patients’ condition | 10 NR  11 Patients of the telemonitoring group took self-measurements more regularly and had internalized the importance of regular self-monitoring. Reception visits were more efficient, no time was wasted on irrelevant issues. Greater adherence to taken drugs more precisely.  Data provided important support for physicians in decisions about the patient’s treatment  12 A potential disadvantage nurse identified issues with the patient inputting results and there was a possibility that some users sometimes inputted false data.  The usage of the nurse’s time was somewhat appeared to have intensified as part of delivery of telemonitoring technology | 13 ‘The telemonitoring increased significantly the nurse’s workload by increasing the number of reception visits and the number of telephone contacts. Extra work is required on top of the multidisciplinary care approach. To lessen the increased workload of health care professionals, the potential of active assistance technology is worthy of further consideration to respond to patients’ queries and to keep them motivated’ (pg8).  14. The increased workload should be carefully considered when implementing telemonitoring in the care of HF patients |
| Wakefield et al (2013)  RCT  USA  ‘To evaluate the efficacy of a nurse managed home tele-health intervention to improve outcomes in veterans with co-morbid diabetes and HTN’ (p254). | 1 NR  2 Home tele-health intervention involving nurse led remote monitoring.  High and low intensity interventions compared  3 High intensity: daily BP, blood glucose as directed. Branching disease management algorithm focusing on diet, exercise, smoking cessation, foot care, medications, weight management, preventative care and behaviour modification and lifestyle modifications. Prompts, questions and education received each day.  Low intensity intervention: daily BP, blood glucose as directed. Smaller subset of questions used, without the branching algorithm.  Usual care: usual follow up appointments in primary care clinic. | 4 Adults (n=302) divided between high intensity (n=93), low intensity (n=102) and usual care (n=107).  5 Comorbid diabetes and hypertension  6 Home  7 Tele Nurses (n=unspecified) | 8 HbA1C: reduced in intervention group during the 6 months of the intervention (P= 0.0003 - 0.0001) but comparable with control group by 6 months after intervention.  Systolic Blood Pressure (SBP): significant reduction for high internsity group at 6 and 12 months (p=0.01)  Adherence: Improved in all groups, with no differences between groups.  9 NR | 10 NR  11 Nurse monitoring and collaboration with a primary care physician  12 NR | 13  Home telehealth increases contact with patients and enables earlier detection of problems. Home telehealth can improve outcomes in patients with comorbid chronic illness in the community.  14 Further research neeeded to establish optimal frequency and intensity for these types of interventions  Home telehealth is a viable intervention for patients with chronic conditions in the community setting. |
| **Algorithim driven** | | | | | |
| Finkelstein et al. (2013).  USA.  RCT  To determine the relative performance of a computer-based Bayesian triage algorithm compared with a manual nurse-based triage system in terms of patient health and health-related quality of life (QOL) in  lung transplant recipients participating in the Home Spirometry Research Program (HSRP). | 1. NR.  2. Home monitoring (HM) of pulmonary function by patients and assessment and interpretation either by specialist nurse or by using the algorithm.  3. Control arm: patients’ Home Monitoring reports assessed by nurse and determined if physician follow up necessary. Intervention: HM reports assessed by the algorithm and determined if need for physician follow up. | 4. Lung transplant patients (n=65) >14 years and discharged from hospital to home stratified and randomised by age >/=50 and </=50 and functional capacity.  5. Lung transplant patients.  6. Home.  7. Research nurses. | 8. Primary clinical was Pulmonary Function Tests (PFT) i.e. Forced Expiratory Volume FEV) % decline from baseline to 1 year and secondary outcome was after 2 years. QOL secondary outcome measured using the SF-36  9. There were no significant differences between groups in FEV1 and SF-36 scores at baseline, indicating the groups were comparable.  A comparison of annual pulmonary function  (FEV1) changes from baseline within each arm showed no significant differences in percentage FEV1 decline between groups after year 1 and year 2. | 10. NR.  11. Use of computerized systems as good as nurse assessment once nurses are educated on such procedures.  12. Quality and safety need to be established with further research . | 13. Could be cost effective if safety is established.  14. NR. |
| **SMS** | | | | | |
| Blank et al. (2011).  USA.  RCT.  *“The objective of this study was to test the effectiveness of a community nursing based APN intervention (PATH) to promote adherence to HIV and psychiatric treatment regimens”* (P. 1318) | 1. NR.  2. Preventing AIDS Through Health (PATH )where an ANP collaborated other health/mental health care providers to help improve adherence and self-care.  3. Minimum of one visit per week for a year. Cascading approach building intensity. Activation of social networks, use of beepers with alphanumeric displays then prepaid phones to encourage particiapnts to follow regimen. Final step was directly observed therapy | 4. Adults 18+ years (n=238)  5. HIV + with Serious Mental Illness.  6. Community based.  7. Community based advanced nurse practitioner. | 8. The intervention group exhibited a significantly greater reduction in lg viral load at 12 months (d=-.361 log 10 copies per milliliter p<.001). Differences in CD4 counts from baseline to 12 months were not statistically significant.  9. Reduction in Viral Load within intervention group. | 10. NR.  11. NR.  12. ANPs in this study were university based and trained in research so it may be difficult to replicate the findings of this study with community based nurses. | 13. A tailored intervention based on the PATH Model and provided by an ANP in the community can improve outcomes for individuals with HIV and co-occurring serious mental illnesses. People with these conditions can be treated successfully with the right range of supports and their viral loads can be reduced.  14. Consider redesigning the health service provision for this vulnerable group to include the use of ANP’s and the PATH model. |
| **Web based** | | | | | |
| Stasiak et al (2014)  Netherlands  A cluster RCT  ‘This study evaluated the effect of E-health4Uth and E-health4Uth and consultation on well-being (ie, mental health status and health-related quality of life) and health behaviors’ Pg 2 | 1. NR  2. Web based intervention  3. Web-based tailored messages (E-health4Uth and E-health4Uth and consultation)  The tailored messages focused on topics related to health-risk behaviors  preventive youth health care setting.  Both interventions used the same Web-based but in the consultation group, adolescents who were at risk of mental health problems were also referred to a school nurse for a consultation.  Adolescents in the control group received no messages following the initial assessment. | 4. Adolescents average 15-16 years of age (n=1702)  5. NR  6. Two youth health care organizations 12 secondary schools  7 School nurse | 8. 4-month follow-up assessing alcohol consumption, smoking, drug use, condom use, mental health via the Strengths and Difficulties Questionnaire , Health related quality of life using Child Health Questionnaire-Child Form  9. Adolescents in the *E-health4Uth group* used condoms significantly more often compared to the control group (52.1% vs 40.6%; OR 2.09). Health-related quality of life in the E-health4Uth group was significantly better compared to the control group (mean 75.34, SD 16.56 vs mean 73.73, SD 18.17 ) Both intervention groups reported a significantly better mental health status compared to adolescents in the control group (SDQ: mean 8.42, SD 5.05 vs 9.07, SD 5.38). No improved effects found on health behaviors from the intervention.  Adolescents in the E-health4Uth and consultation group, who were at risk of mental health problems and referred for consultation with the nurse, had significantly better mental health status (SDQ: mean 12.79, SD 5.63 vs 14.57, SD 5.03) and a better health-related quality of life (mean 69.56, SD 18.37 vs 62.53, SD 20.08) at follow-up than those in the E-health4Uth group and the control | 10 NR  11 NR  12NR | 13 ‘Findings from this study support the use of the E-health4Uth and consultation intervention in promoting the well-being of adolescents at risk of mental health problems’.Pg13  14. ‘Future research is needed to further evaluate the effects of the consultation as a standalone intervention, and the dual approach of further tailored eHealth messages and a consultation’. (Pg 13) |
| Finkelstein et al 2011  RCT  USA  ‘To evaluate the perception, satisfaction and utilisation of a home telehealth service for frail elderly people living independently in their home communities’ (p288) | 1 NR  2 Virtual Assisted Living Umbrella for the Elderly (VALUE) Home telehealth service  3 Web portal which allowed videoconferencing and electronic messaging between nurses and clients. This provided access to health information, ordering health and home care services, e.g. Medication refills and appointments, and general internet access.  Physiological monitoring deices were used appropriate to patients’ health conditions. | 4 Older adults (n=99)  5 Frail  6 Home  7 Nurses (n=unspecified) | 8 Perception of telehealth service: intervention group significantly more positive towards technology at 60 days compared with their baseline scores , and compared with the control group at 60 days (P<0.001)  Satisfaction: Intervention group reported that the system met their overall expectations (mean+ 9 out of 10), and would recommend it to others (Mean= 9.5 out of 10).  Utilisation: All able to use portal effectively after a demonstration by the nurse. 5% of videoconferencing calls disconnected due to technical difficulties  Reduced rates of ED visits and home care use among intervention group following intervention, compared with control group. Higher rates of visits to eye care doctor among intervention group (P=0.008).  Lower use of transportation services among intervention group than controls following intervention (P=0.017).  9 NR | 10 Telehealth intervention was reported by some patients as saving them money.  11 Simple system and basic instructions from nurses necessary for participants to learn to use system.  12 Lack of Broadband availability in some areas | 13 Frail older people were able to utilise a home telehealth system easily and this was associated with a decrease in healthcare utilisation. The system also appeared to have high levels of patient satisfaction.  14 Web portal home telehealth system was effective and had high rates of patient satisfaction and so could be implemented in other settings. |

# Nursing in Community with Non-Professional Community Health Workers

| **Miscellaneous** | | | | | | | | | | |
| --- | --- | --- | --- | --- | --- | --- | --- | --- | --- | --- |
| Filene et al. (2013).  Meta-analysis.  (n=51 studies. Type of studies NR other than noting that effect sizes could be calculated from studies).  “*to determine which individual home visiting program components*  *have the most power to predict key parent and child outcomes*” (p. S101). | 1. NR   2. NR other than home visiting  3. Typical child development information, child care & routines, focus on home, environment – safety & cleanliness,  attending to child’s emotional needs,  promoting cognitive & socio-emotional development,  instrumental assistance,  Selecting alternative caregivers, parental relationships & health,  parenting practices,  family planning,  self-, stress & anger management, support & social networks, adult literacy & academic achievements, problem solving, goal setting,  case management,  rehearsal or role-playing, matching home visitor to client e.g. race, ethnicity,  standardized curriculum. | | | | 4. Pregnant women and families with children from birth to 3 years in U (n=unspecified)  5. Early childhood development (typically developing).  6. Children’s family homes and clinics  7. Professional (e.g. nurse, psychologist, social worker) & non-professional home visitors. | | 8. Clinical/Health outcomes: (6 in total)  Maternal life course, child cognitive outcomes & parenting behavior & skills: significant positive average effect sizes.  Birth outcomes, child physical health, & child. Maltreatment: no significant differences in effect sizes.  9. Non-professionals, matching of home visitors to clients & problem solving associated improved birth outcomes (e.g. birthweight, absence of complications).  Parent education on developmental norms, behavioural management & discipline techniques, responsive associated with improved parent behaviours & skills outcomes.  Parenting practices associated with better parenting behaviours & skills and with cognitive outcomes in children.  Professional home visitor predicted better child physical health,  Parent support groups associated with small effect sizes for child physical health,  Selecting alternative caregivers & problem solving associated with better outcomes for child maltreatment.  Note: Based on sensitivity analysis effects of problem solving on birth outcomes no longer significant. | 10. NR.  11. NR.  12 NR. | | 13. NR.  14. NR. |
| Tafts et al (2015).  Australia.  RCT  “*To test whether a theory-informed, maternal and child health (MCH) nurse-*  *designed model increased and sustained DV screening, disclosure, safety planning*  *and referrals compared with usual care”* (p1). | 1. NR.  2 MOVE model  (Intervention informed by Normalisation Process Theory, a nurse-designed good practice model).  3. Intervention: nurse mentors, strengthened relationships with DV services, nurse safety, a self-completion maternal health screening checklist at three or four month consultations and DV clinical guidelines. Usual care: government mandated face-to-face DV screening at four weeks postpartum and follow-up as required. | | | | 4. Community based maternal and child health teams (n=8).  5. Maternal and child health.  6. Community.  7. Nurses (n=163), and domestic violence liaison workers. | | 8. Primary: women screened for DV; Women’s disclosure and nurse safety planning; Referrals. Secondary: Prevalence of any DV in the previous 12 months; DV during pregnancy and maternal reporting of abuse as a child; difference in proportions of women reporting harm.  MCH team screening: No significant differences between groups at 4 months but increased proportion of screening (36.5% in intervention vs 23.5% in controls). Maternal heath checklists at 3 months show average screening rate of 63.1%.  Safety planning rates: Increased for intervention group, 3 to 4 times higher than control group, at 2 year follow up ((RR 2.95, CI 1.11–7.82) to four times those of CG (RR 4.22 CI 1.64–10.9).  9. Self-completion DV screening was  welcomed by nurses and women and contributed to sustainability. | 10. NR.  11. Longer screening time focused on the mother and her needs.  12 MOVE process evaluation identified  implementation barriers, such as lack of nurse reflective practice and the coinciding introduction of a  new practice framework.  Using survey data online may reduce response rate. | | 13. Safety planning rates may be improved by nurse designed model of screening and care. The involvement  of staff is critical both for effectiveness and sustainability of DV interventions in health care settings  14. Greater attention should be given to how screening is implemented in primary care and further research should be undertaken on intermediate outcomes such as safety planning and its benefits. |
| **Community Health Workers** | | | | | | | | | | |
| Allen et al. (2011).  USA.  RCT.  To evaluate the clinical effectiveness of a comprehensive program (COACH) of CVD risk reduction delivered by teams of nurse practitioner(NP)/ Community health Worker (CHW).  HM | 1. NR.  2. Risk reduction based on a theoretical framework using community-based participatory research (CBPR).  3. EUC = Enhanced usual care by primary provider containing feedback regarding CVD risk factors to the patient and their provider.  COACH = Community Outreach and Cardio-vascular Health which focused on behavioural interventions to bring about therapeutic lifestyle changes (TLC), medication adherence , attendance at appointments as well as prescription and titration of medications. The NP functioned as a case coordinator for each participant and was delivered by home visit and telephone. | | | | 4. Adults (n=525) > 21yrs old.  5. Documented cardio-vascular disease (CVD), type 2 diabetes.  6. Community health clinics.  7. Nurse practitioner (NP). | | 8. Patients in COACH compared to EUC group had significantly greater improvement in total cholesterol(difference,19.7mg/dL), LDL cholesterol (difference,15.9 mg/dL), triglycerides (difference, 16.3 mg/dL), diastolic blood pressure (difference, 3.1 mm Hg), systolic blood pressure (difference, 6.2 mm Hg), HbA1c (difference, 0.5%), and perceptions of care quality of their chronic illness (difference,1.2 points) at one year.  9. It was considered that it was the combination of NP/CHW in the intervention that achieved significant improvement in CVD risk profiles. | 10. NR.  11. Using CHWs was considered an enabler as the trust between them and the patient was considered to enhance the intervention delivery.  12. Authors suggested that the higher dropout rate in the intervention group may have been due to the increased commitment to participate in more visits to the clinic resulting in higher costs to participants. | | 13. An intervention delivered by a team of NP/CHWs using customised treatment regimens based on treat-to-target algorithms can be an effective approach to improve risk factor status and perceptions of chronic illness care in hard to reach high-risk patients.  14. Further analyses will evaluate the cost effectiveness of NP/CHW model. Further study is needed to determine if this type of intervention translates into improved morbidity and mortality from CVD. |
| Allen et al. (2014).  USA  RCT further reporting on RCT by Allen et al 2011.  To analyse the cost-effectiveness  of a comprehensive program (COACH) of CVD risk reduction delivered by teams of nurse practitioner(NP)/ Community health Worker (CHW) *“to improve lipids, blood pressure, and HbA1c levels in patients in federally-qualified metropolitan community health centers”* (p.1) | 1, 2 &3 as above in Allen et al 2011 | | | | 4. Adults (n=525) > 21yrs old. Costs collected on a sample of 30% of intervention patients for one year compared costs calculated on a chart review with a sample of UC patients.  5. Documented cardio-vascular disease (CVD), type 2 diabetes.   1. Community health clinics.   7. Nurse practitioner (NP). | | 8. 70% of participants had at least four in-person visits with the nurse. The NP averaged 17 (CI 16, 19) minutes per direct encounter time with the patient and another 16 (CI 14, 17) minutes for non-encounter activities. The highest percentage of time with the patient was spent in counselling regarding medications (43%). In terms of cost-effectiveness over 1 year: $157 for every % drop in systolic blood pressure and $190 for every % drop in diastolic blood pressure; $149 per % drop in HbA1c; and $40 per % drop in LDL-C. Exceptionally for HbA1c, $1255 for a drop of one unit (i.e. from 8% to 7%).  9. The total cost of intervention from the NP/CHW team exceeded the cost for  Medical Doctor (MD) care; however, the average per patient incremental total cost (NP/CHW –without MD) was only $627. | 10. NP and CHW time spent delivering the intervention – number of visits’ time and the types of activities; time for preparation and follow-up activities; consultation with other health care providers, contacting the pharmacy or insurance agency. Hourly salary including benefits for the NP ($51.14) and the CHW ($25.78). Other provided, lab and drug costs were calculated and analysed.  11. Using CHWs was considered an enabler as the trust between them and the patient was considered to enhance the intervention delivery.  12. NR. | | 13. A nurse-led team which includes CHWs is a clinically and cost- effective model of care.  14. Evidence-based treatment algorithms should be used as they are a successful, cost-effective, strategy to implement national guidelines to manage of, hypertension, hyperlipidaemia and diabetes in high risk vulnerable populations. |
| Hamid et al (2014).  USA.  RCT  The focus of this study was “*to examine the impact of a successful 12 month behavioral intervention to improve diabetes control on healthcare utilization in American Samoa*” (p.1). | 1. NR.  2. Nurse- community health worker intervention focused on diabetic control over a 12 month period in comparison to usual diabetic care  3. 12 month nurse-community health worker (CHW) team intervention, compared to usual care. | | | | 4. Samoan adults with type 2 diabetes (n=unspecified)  5. Diabetes type 2.  6. Community primary health care centre.  7. Nurse case manager (NCM) and nurse-community health worker (CHW) | | 8. Healthcare utilization, including ED visits, hospitalizations, primary care physician (PCP) visits, association of utilization with change in HbA1c.  With regard to ED visits, hospitalisation and PCP visits at 12 month follow up this study reports a statistically significant increase in PCP visits in the CHW group during the intervention year.  9. The CHW intervention had a greater effect in reducing ED visits in those who had more ED visits before study enrollment; each additional ED visit before enrollment was associated with a 20% (95% CI: 12%, 26%) reduction in the relative risk of ED visits during the intervention year.  The 12-month CHW diabetes intervention increased primary care utilization by 71% in the CHW group compared to the usual care group, | 10. Nurse- CHW diabetes intervention increases primary care utilization by 71% in the CHW group compared to the usual care group. This increased utilisation results in improved diabetic control, self-management and monitoring.  11. NR.  12. NR. | | 13. This study reports the positive input of CHW and PCP visits with regards to diabetic control, self -management behaviours and overall monitoring of the condition.  14. This study makes recommendations for further research linked with conducting a cost effectiveness analysis of healthcare utilization, and use of longer follow up periods for evaluation of long-term impacts of CHW interventions on utilization. |
| Meghea et al. (2012).  USA.  RCT  To evaluate *‘the advantage of an enhanced prenatal and postnatal services (EPPS) programme using nurse–CHW home visitation in improving infant health in low-income families, when compared with usual community care (CC) including nurse home visitation’* (p.28). | 1. ‘Home visitors are  usually nurses who assess and manage health problems, co-ordinate care, provide health education and other activities to improve maternal and child health and development’ (p.27).  2. Home visitation programme, state sponsored through Medicaid enhanced prenatal & postnatal services (ESSP) with the addition of nurse-CHW home visits.  3. Standard Medicaid ESSP during pregnancy & 1^st^ 12 months of life: Risk screening, transportation, psychosocial counselling, nutritional guidance  and pregnancy and parenting education, primarily through  home visitation by a nurse during pregnancy.  Additional components for IG were CHW phone contacts & home visits with specific activity modules: self-esteem; positive health behaviors; self-awareness of stressors, causes of stressors and active problem solving; self-determination through development of personal life goals; and community resources. | | | | 4. Medicaid-insured pregnant women (n=530).  5. Pre-natal & post natal infant health care.  6. Home visits.  7. Community Care nurses (IG & CG groups) & community health workers (CHW – IG only). | | 8. Outcomes Assessed: (IG vs CG)  Fewer incidences of mother-reported asthma/wheezing/croup among babies whose mothers have low psychosocial resources (13% vs. 27%, P = 0.01) & whose mothers have low psychosocial resources and high stress (17% vs. 29%, P = 0.08).  No other statistically significant infant health effects of the nurse–CHW team home visitation programme compared with CG. in the subgroups defined by low psychosocial resources and high stress.  No improvements in recurrent ear infections or feeding problems. No differences in immunizations, hospitalisations or ear infections.  9. No applicable since IG Nurse–CHW team intervention did not improve general infant health compared with standard CC including nurse home visiting. | 10. NR.  11. Targeting such team interventions at common health problems of infancy and childhood or at managing diagnosed chronic conditions may prove more successful.  12. NR. | | 13. There is no strong evidence that infant health in low-income families was improved by the addition of CHWs to a programme of enhanced prenatal/postnatal services that included nurse-only home visitation.  14. Cost effectiveness research on home visiting programmes needed. |
| **Volunteer** | | | | | | | | | | |
| Kemp et al. (2011).  Australia.  RCT  *“To investigate the impact of a long-term*  *nurse home visiting programme, embedded within a universal child health system, on the health, development*  *and well-being of the child, mother and family”* (p1). | 1. NR.  2. MECSH: Structured nurse home visiting and parenting education and  support programme vs usual universal care.  3. Structured home visits, post natal child development education, access to secondary and tertiary early childhood health services, volunteer home visiting  services and family support services within the local area, group activities and community links. | | 4. At-risk mothers living in a  socioeconomically disadvantaged area (n=208).  5. Child and maternal health.  6. Home care, socioeconomically disadvantaged area.  7. Child and family health nurses. | | | 8. Primary Outcomes: Quality of the home environment for child development (12–24 months): parent child interaction during free play; Child mental, psychomotor and behavioural development at 18 months.  Perinatal outcomes: low birth weight and preterm birth, extracted from hospital clinical records.  Child respiratory and gastrointestinal illness and immunization status, measured by maternal self-report, being fully immunized or not at child-age 24 months.  Maternal and household smoking during pregnancy and over the first 2 years of life.  Breastfeeding over the first 2 years of life,  Maternal health (SF-12), depression (EDS) and social support, the number of stressful life events in the previous year and family functioning, and experience of being a mother.  Parent–child interaction: No significant difference in parent–child interaction between the intervention and comparison groups  Mothers receiving the intervention were  more emotionally and verbally responsive than comparison group mothers (mean difference 0.5; 95% CI 0.1 to 0.9). Duration of breastfeeding was longer for intervention mothers than comparison mothers (mean difference 7.9 weeks; 95% CI 2.9 to 12.9).  Child mental, psychomotor and behavioural development at 18 months: no significant overall group differences in child mental, psychomotor or behavioural development.  Birth weight: No significant difference between groups.  Mothers assessed antenatally as having psychosocial distress benefitted from the intervention across a number of areas.  9. NR | | | 10. NR.  11. NR.  12. The number of participating nurses, and hence mothers, was  limited by the funding, infrastructure and management capacity within the service. | 13. The MECSH programme showed that in many outcome areas there were no differences in outcomes for first-time  and multiparous mothers. This challenges the assumptions of many nurse home visiting programmes that their benefits are confined to first-time mothers.  14. The MECSH trial showed some significant results and some trends that require replication in larger samples of mothers drawn from a similarly widely defined at-risk group, including older, multiparous mothers, and mothers with higher levels of education. |
| **Lay personnel** | | | | | | | | | | |
| Dennis and Dowswell. (2013).  Cochrane Systematic Review  To assess the effect of psychosocial and psychological interventions to reduce the risk of developing postpartum depression. | | 1. NR.  2. Postpartum home visits provided by public health nurses or midwives/ lay (peer)-based telephone support/ interpersonal psychotherapy  3. Psychosocial and psychological interventions vs usual AN/,PN care to reduce risk PND. Also to examine (1) effectiveness types interventions, (2) effectiveness of professional versus lay interventions, (3) effectiveness individual vs group interventions, (4) effects of intervention onset and duration, (5) whether interventions more effective in women selected with specific risk factors. | | 4. 28 trials, N= 16912/ almost 17,000 pregnant women and new mothers including adolescents and younger mothers.  5. Primary care.  6. Postnatal depression- EPDS used most frequently to assess but other tools also used.  7. Nurses, midwives and others (physicians, mental health specialists, therapists, lay (peer) individuals. | | 8. Less prevalence of PND: outcome assessments varied considerably between studies, ranging from three to 28 weeks.  9. Psychosocial interventions: antenatal/postnatal classes/groups, home visits/ telephone support/, early postpartum support/ continuity of care models.  Psychological interventions: debriefing, CBT, interpersonal psychotherapy.  There was beneficial effect on the prevention of depressive symptomatology in the meta-analysis of all types of psychosocial and psychological interventions.  Intervention group significantly less likely to develop PND (ave RR 0.78, 95% CI 0.66 to 0.93; 20 trials, 14,727 women).  Promising interventions:  (1) intensive, home visits by PHNs or midwives (RR 0.56, 95% CI 0.43 to 0.73; two trials, 1262 women);  (2) lay (peer)-based telephone support (RR 0.54, 95% CI 0.38 to 0.77; one trial, 612 women);  (3) interpersonal psychotherapy (st m diff -0.27, 95%CI -0.52 to -0.01; five trials, 366 women).  Professional- and lay-based interventions were both effective in reducing the risk to develop depressive symptomatology.  Individually-based interventions reduced depressive symptomatology at final assessment (RR 0.75, 95% CI 0.61 to 0.92; 14 trials, 12,914 women) as did multiple contact interventions (RR 0.78, 95% CI 0.66 to 0.93; 16 trials, 11,850 women). Interventions that were initiated in the postpartum period also significantly reduced the risk to develop depressive symptomatology (RR 0.73, 95% CI 0.59 to 0.90; 12 trials, 12,786 women). Identifying mothers ’at-risk’ assisted the prevention of postpartum depression (RR 0.66, 95% CI 0.50 to 0.88; eight trials, 1853 women). | | | 10. NR.  11. Midwifery-based flexible postpartum care and lay telephone support interventions incorporated screening with the Edinburgh Postnatal Depression Scale (EPDS) for the early identification of depressive symptomatology  12. NR. | 13. Psychosocial and psychological interventions significantly reduce the number of women who develop postpartum depression. No evidence that a specific health professional providing an intervention increased the likelihood of a preventative effect.  14. Interventions that are individually based and initiated postnatally may be beneficial. Interventions targeting ’at-risk’ mothers may be more beneficial and feasible than those including a general maternal population. |
| Radcliffe et al. (2013).  USA.  RCT  *“To demonstrate whether participation in this home visiting program led to differential changes in referral to early intervention*  *and receipt of early*  *intervention services”* (p.S154). | | 1. Home visiting is a programme designed *“to improve the physical and mental health of mothers,*  *such as increasing time to subsequent pregnancies and improving parenting*  *skills”* (S154). Noted: that such programmes usually run in parallel to child health programmes but can be in collaboration with paediatric services.  2. IG: the MOM Programme which is *“an innovative home visiting program*  *designed to serve urban, low-income mothers and children’* (p. S154). Focuses on both parents and child health i.e. home visits and well child clinics.  Delivered over 3 years  CFG: no home visits.  3. Home visits (HV) pre clinic visits (CV); use of a team of home visitors rather than a single visitor per family; weekly supervision meetings to monitor the progress of all children enrolled; structured, model-driven checklists for each visit; use of regular reminder calls before HV & before/ after CVs. | | 4. Mothers from areas with high poverty rates and had to have given birth to a singleton healthy infant (weight 2500 g; no identified genetic or developmental disorders).  302 recruited. 89% retained (n=271).  5. NR.  6. Home visits and scheduled well child clinics.  7. Home visitors included both lay workers and  pediatric nurse practitioners. | | 8. Outcomes Assessed: Time staff spent with each family per month: no statistically significant differences between groups. Likelihood of recurring health visits: Home-visited mothers in the IG were 10.77 times as likely to keep pediatric appointments, compared with those not visited (OR: 10.77 [95% confidence interval (CI): 6.05–19.17]; P<.0001.).  9. The number of home visits (completion of at least 7 out of 9 visits). | | | 10. NR.  11. NR.  12. Staff at child clinics often discouraged mothers from these visits or refused to makeappointments unless child was ill. | 13. Home visiting programs can provide important partnerships with pediatric health care providers. Integrating home visiting services with pediatric care can enhance child health, and warrant expansion.  14. Schedule home visits just before scheduled clinic visits. |
| Segal et al. (2012).  Systematic Review (Nonrandomised controlled studies n=14; cohort studies n=2; RCTS n=36).  *“to gain a new understanding of the home visiting literature for the prevention of child maltreatment by taking a program logic approach that incorporated a theory of change”* (p55). | | 1. NR.  2 Home visiting programs.  3. Mean number of  visits ranged from 2-41, and the length of visit varied from  20 mins – 4 hours. home-visiting programs were, in order of frequency: education/  training/information (*n* = 46), emotional support (*n* = 43), referral and linking to services/advocacy (*n* = 38), modeling/role model (*n* = 19), problem solving (*n* = 16), counseling/therapy (*n* = 16), case management (*n* = 5), provision of goods and services (*n* = 5), responsive clinical services (*n* = 2), and provision of child care (*n* = 2). | | 4. Teenage/adolescent parent (n=7); high-risk families (n=4); parents using illicit drugs (n=2). Population of many programs overlapped two or three risk categories. Most programs targeted people at elevated risk e.g. abuse, current drug or alcohol problems, or several risk characteristics (*n* = 23).  5. Child abuse and maltreatment.  6. Home.  7. Nurses most often involved in home visiting (*n* = 19), but also social workers (*n* = 15),  “paraprofessionals” (*n* = 9) or laypersons (*n* = 6). Formal multidisciplinary team (n=3). | | 8. *Overall Success of the Program/Intervention Arm:* 25 (48%) of programs were  defined as successful and 27 (52%) not successful  *Relationship between Match and Program Success:* For the 7 programs for which a complete match was observed *between Theory, Population, Program* *Components, and Child Abuse Prevention*  *Objective* , all were defined as successful  For the 15 programs for which a clear mismatch was observed, none was defined as successful; that is, the home-visiting group did no better than the  control in any of these programs.  9. Studies failed to find any single program component e.g. type of professional, timing of intervention, or target population to predict the success of home visiting. | | | 10. Need for adequate resources, appropriately skilled team, access to training and quality assurance processes.  11. Appropriately skilled team; access to training and quality assurance processes, understanding of population, their needs and strengths, and underlying theory regarding mechanisms of change.  12. NR. | 13. A match between the underpinning theory and program components together with a match between the theory and target population predicted program success.  14. Results suggest that a way to maximize success  is through fidelity to a program logic model with clear objectives to ensure understanding of population’s needs , associated theory or mechanisms of change.  Adopting “off-the-shelf £program models” is no guarantee of success, particularly if they are not designed for the target population and their specific circumstances. |
| Turnbull et al. (2012).  Australia.  Systematic Review (n=7 random or quasi-randomised trials).  *“To determine the effects of home visits during pregnancy and/or after birth for women with a drug or alcohol problem*” (p1). | | 1. NR.  2. Home visits mostly after birth vs no home visits.  3. Home visits that commenced during pregnancy and/or after birth by teams or individuals consisting of doctors (obstetricians, general practitioners or paediatricians), nurses (midwives, drug and  alcohol workers or early childhood nurses), social workers, counselors or trained lay people. | | 1. Pregnant or postpartum women (n=unspecified)   5.Pregnant or postpartum women with a drug or alcohol problem.  6. Home visiting.  7. Community health nurses, paediatric nurses, trained counsellors, paraprofessional advocates, midwives. | | 8. A very large number of outcomes associated with drug / alcohol; Pregnancy/ Postpartum; Infant/child; psychosocial and economic outcomes were considered.  9.There was no significant difference in main outcomes i.e. continued illicit drug use (three studies, 384 women; risk ratio (RR) 1.05, 95% confidence interval (CI) 0.89 to 1.24), continued alcohol use (three studies, 379 women; RR  1.18, 95% CI 0.96 to 1.46), failure to enrol in a drug treatment program (two studies, 211 women; RR 0.45, 95% CI 0.10 to 1.94), not breastfeeding at six months (two studies, 260 infants; RR 0.95, 95% CI 0.83 to 1.10), incomplete six-month infant vaccination schedule (two studies, 260 infants; RR 1.09, 95% CI 0.91 to 1.32), the Bayley Mental Development Index (three studies, 199 infants;  mean difference 2.89, 95% CI -1.17 to 6.95) or Psychomotor Index (MD 3.14, 95% CI -0.03 to 6.32), child behavioural problems (RR 0.46, 95% CI 0.21 to 1.01), infants not in care of biological mother (two studies, 254 infants; RR 0.83, 95% CI 0.50 to 1.39), non-accidental injury and non-voluntary foster care (two studies, 254 infants; RR 0.16, 95% CI 0.02 to 1.23) or infant death (three  studies, 288 infants; RR 0.70, 95% CI 0.12 to 4.16). Individual studies reported a significant reduction in involvement with child protective services (RR 0.38, 95% CI 0.20 to 0.74) and failure to use postpartum contraception (RR 0.41, 95% CI 0.20 to 0.82). | | | 10. NR.  11. Well trained nurse; frequent visits; trusting relationships;  short term intense interventions by trained counselors.  12. Home violence; lay home visitors. | 13. Insufficient evidence to recommend the routine use of home visits for pregnant or postpartum women with a drug or alcohol problem.  14. Further large, high-quality trials are needed particularly those incorporating antenatal home visits with  encouraging pregnant women with a drug or alcohol problem to  access early and frequent antenatal care, stabilise drug use, reduce or eliminate alcohol use in pregnancy and remain engaged with  services during their child’s first years of life. Trials also need to include dynamic models incorporating case management. |

# Preventative Focus

| **Miscellaneous** | | | | | |
| --- | --- | --- | --- | --- | --- |
| Fahs et al (2012).  USA.  Multisite RCT  *“To compare 2 strategies, stage-matched nursing and community intervention*  *(SMN+CI) and community intervention (CI) alone in changing cardiovascular risk factors in up to 3 behavioral areas: diet, physical activity,*  *and/or smoking among rural women”. P248* | 1. NR.  2. Transtheoretical model included assessment of stage of change (SOC) at 5 levels. The Moos model addressed environmental system limitations associated with rural lifestyle.  3. CI – invite to attend a community visioning meeting to discuss ways to increase awareness of female CVD and local resources available to improve heart health. Included websites and a Church based health fair.  SMN+CI – developed using Transtheoretical Model (TM) across multiple behaviors. Interventions were team developed, designed according to the 10 processes of change, and consideration of Moos model. | 4. 274 Rural Women age 35-65 years (n=117).  5. Framingham score  ≤20 points with no history of coronary heart disease (CHD) or diabetes.  6. 2 rural counties in US to enable ethnic mix.  7. 12 Registered Nurses delivered interventions using protocols developed by a TM consultant. | 8. SMN+CI group had higher fruit intake by meal (P = .005) and showed greater pre-post change in total fruit and vegetable intake (P = .03). There was no significant difference on Framingham by group after the intervention (t (115) = 1.07, P = .28), yet, Framingham scores decreased significantly for the entire sample (t (116) = 6.01, P = .000). Impact on other outcomes e.g. physical activity and reducing lipids were not significant between group  9. Targeted nursing intervention had the largest effect on fruit and vegetable intake. | 10. 2 day workshop to train nurses.  11. NR.  12. 21% attrition usual for such studies. | 13. More work needs to be done to reduce CVD risks to promote heart health among rural women.  14. Nursing interventions focused on lowering cholesterol through behaviour modification should receive consideration. A design focusing on intervention fidelity, a larger sample size, and ways of preventing contamination across interventions in small rural areas is needed. Further work needs to be done among rural women on the most effective means of reducing CVD risks, including anthropometric measures. Rural women and particularly black rural women are often underrepresented in CVD research. |

| **Child Welfare /Child health** | | | | | |
| --- | --- | --- | --- | --- | --- |
| Kempe et al. (2014).  USA.  RCT  *“To assess effectiveness and feasibility of public–private collaboration in delivering influenza immunization to children”* (p110). | 1. NR.  2. Joint community clinics and Public Health Department nurses aiding in delivery at practices vs usual care (without Public Health Dept. Nurses).  3. NR. | 4. Children attending paediatric/ family medicine practices (n= unspecified).  5. Influenza (vaccine).  6. Pediatric and family  medicine private practices with a single common public health department (n= 7 sites) .  7 Public Health Department nurses. | 8. Primary outcome: receipt of one influenza vaccine  Secondary outcome: receipt of at least one vaccine within: different age groups and children with a high-risk medical condition.  9. Receipt of at least one influenza vaccination at end of each study year for each eligible child: Overall rates increased from baseline to Y2 by 9.2% in intervention and 3.2% in control (p b .0001).  Secondary outcomes:  Receipt of at least one vaccine within different age groups (6 months to 5 years, 6–12 years, and 13–18 years): largest increases seen among school-aged and adolescent children. Differences for 6-month-old to 5-year-old children did not reach significance.  Comparison of missed opportunities (yes/no) for each eligible child during the second intervention year between intervention and control practices: 51.3% (n= 5490) of children in the control practices missed an opportunity for influenza vaccines, compared to 40.8% in intervention group (n= 7078) (p b .0001). | 10. Regarding collaboration in vaccine supply, there is potential for financial loss if quantity needed is misjudged.  11. Further collaboration between public and private; pooling of vaccine supplies between collaborators  12.Barriers to collaboration included uncertainty  regarding the delivery of vaccine supplies, concerns about using up all purchased vaccine by practices, and  concerns about documentation of vaccination if collaboration occurred. | 13. Public–private collaboration resulted in significantly higher influenza  immunization rates, particularly for older, healthy children who visit providers less frequently.  14. A coordinated community-wide  approach is required, involving all potential vaccinators, schools,  pre-schools, and pharmacies, is needed to enable universal yearly vaccination to be achieved. |
| Kokkvoll et al. (2013).  Norway.  RCT  To compare a new comprehensive lifestyle programme performed in groups of families with overweight (included obese) children with a more conventional single family programme. | 1. NR.  2. Family based intervention.  3. Multiple family intervention (MUFI) compromised of a 3 day inpatient programme at the hospital with other family and interdisciplinary team. Single family intervention (SIFI) comprised of individual counselling by pediatric nurse consultant and nutritionist at the hospital. Both interventions had community based follow-up. | 4. Children aged 6-12 years (n=97).  5. Cut off point was children with a BMI of ≥27.5.  6. Community Health.  7. Pediatric Nurse. | 8. BMI increased by 0.37units in the MUFI compared to 0.77units in the SIFI (P=0.18). BMI SDS decreased by 0.16 units in the MUFI group compared to 0.07 units in the SIFI group (P=0.07).  9. Interim analysis after 12 months showed no between group difference in terms of BMI or BMI SDS. The MUFI group had a significant decrease in waist circumference compared to the SIFI group. | 10. NR.  11. NR.  12. NR. | 13. The modest difference between the two treatment groups after 2 years raises the question whether the cost of the MUFI approach can be justified**.**  14. Obesity interventions in children and adolescents should examine health and evaluate mental health and wellbeing in addition to other health outcomes. |
| **School Nurses** | | | | | |
| Ahmad et al. (2011)  Systematic Review (n=9 studies)  USA  To evaluate ‘the effects of asthma self-management education for schoolage children on number of school days missed, emergency department visits and hospital admissions’ (p  282). | 1 School based asthma education programmes: ‘teach self-management knowledge and skills to children and adolescents with a diagnosis of asthma’ (p282). Aims to improve quality of life and reduce morbidity and mortality related to asthma.  2 School based asthma self-management education programme  Refers to Health Promotion Model for use as a functional tool to examine relationships between variables in the delivery of health education. Health Promotion Model includes focus on self-efficacy.  3 Several studies reported on the Open Airways for Students programme. | 4 Children and adolescents (n= 4163 in total)  5 Asthma  6 School  7 School nurses and school staff (n=unspecified) | 8. Missed school days: Decrease in absenteeism in all studies. Statistically significant decrease in school days missed in 6 studies  However. authors state it is unknown whether this effect persists beyond 1 year after intervention.  Number of ED visits: 4 studies reported decrease in number of ED visits following the intervention.  Hospitalisations within 1^st^ year of completion: 2 studies reported statistically significant decrease in asthma-related hospitalisations.  9 Repetition and reinforcement of knowledge is needed. | 10 NR  11 Convenient location needed.  12 NR | 13 Asthma self management education is important and can be effective. Provision of this through schools and using school nurses can improve access to such programmes.  14 School based asthma self management education programmes should be widely implemented.  Programmes should include repetition and reinforcement of information.  School nurses are in an excellent position to implement such programmes as they can see students on a regular basis.  More detailed research is needed regarding the effect of self management education for children on the child’s ability to take their medication properly |
| Bannink et al. (2014).  The Netherlands.  Cluster RCT  To *“evaluate the appreciation, application*  *and effects of an intervention (Your Health), in which adolescents received a consultation with the school nurse”* (p.773). | 1. NR.  2. “Your Health” Intervention.  3. Consultation with the  school nurse utilising structured assessment tools (Self Sufficiency Matrix -SSM-D). The nurses role included support; health promotion; referral to HCP. | 4. Adolescents (n=418).  5. Behavioral problems.  6. School.  7. School nurse. | 8. Primary outcomes: adolescents’ mental health (i.e. mental health status and depressive symptoms); school absenteeism; debts. Secondary outcomes: quality of life; alcohol consumption; and soft drug use.  9. No statistically significant effects were seen within the “Your Health” intervention at 6-month post-intervention assessment compared with the control group (all P>0.05) in relation to mental health status; depressive symptoms; school absenteeism; debts.  No statistically significant differences were identified in relation to quality of life; alcohol consumption; soft drug use either (all P>0.05). | 10. NR.  11. NR.  12. NR. | 13. Your Health” may have potential to be an important intervention for vulnerable adolescents at the beginning of “vocational education.  14. Further studies should assess the suitability and effects of referrals and the subsequent help that adolescent participants attained during consultation; this intervention can be simply embedded into existing practice. “Your Health” could be enhanced by strengthening collaboration between the school nurses and the other school health professionals. |
| Borawski et al. (2014).  USA.  Group-randomized intervention study  To *“examine whether*  *the effectiveness (i.e. improved knowledge, self-efficacy, intentions, compared to a control group) of a well-established HIV/STI prevention curricula (Be*  *Proud! Be Responsible! [BPBR]) would vary based on facilitator type (health education classroom teacher vs school nurse)”* (p.190) | 1. NR.  2. The BPBR curriculum consists of 6 teaching modules (50 minutes each).  3. Teaching methods i.e. group discussions; role model stories depicted in videos; interactive exercises; and role-playing. | 4. High schools (n=10), participants (n=1576).  5. Sexually transmitted infections.  6. High Schools (n=10).  7. School Nurses. | 8. Knowledge; Intentions; Efficacy; Beliefs; Perceived peer beliefs; Curriculum fidelity; Descriptive characteristics; Facilitator performance; and student assessment.  9. Students gave higher ratings to the classroom teacher facilitators than to the school nurse in relation to: how comfortable the facilitator was with materials (p<.006); extent to which the curriculum challenged how students thought about their health (p<.02); classroom environment being more orderly (p<.07). A significant increase was noted in students HIV/STI and condom use knowledge immediately post-intervention and at 4 months (p<0.5). A change in student intentions when taught by school nurses was reported in relation to the use of a condom. A significant increase was noted in technical skills and condom negotiation skills. Intervention students taught by school nurses reported significant increases in their condom use beliefs at 4 months. | 10. NR.  11. NR.  12. NR. | 13. Both classroom teachers and school nurses are effective in conveying reproductive health information to high school students; however, teaching the technical (e.g., condom use) and interpersonal (e.g., negotiation) skills needed to reduce high-risk sexual behavior may require a unique set of skills and experiences that health education teachers may not typically have.  14. Utilising school nurse resources may improve reproductive health teaching as students may be more comfortable and familiar with healthcare professionals. For this intervention to be effective in influencing risk behaviors, schools should utilise school nurses in teaching the technical and interpersonal skills  needed for self-protection. |
| Brackney et al. (2015).  USA.  Systematic Review (n=35).  To “*determine effective nonpharmacological interventions for prevention of T2 DM in youth*” (p.6) | 1. NR.  2. Non-pharmocological interventions.  3. Included: Daily activity; decreasing caloric intake; increasing muscle mass; psychological and social support | 4. Adolescents (15- 19 years).  5. Type two diabetes.  6. School; Family; Community; and Clinic.  7. School Nurse. | 8. School based interventions (n=9); Family based interventions (n=4); Community based interventions (n=7); Clinic Interventions (n=15).  9. Components for effective intervention strategies include; different types of physical activity; nutrition education; behavior therapy; cultural sensitivity; and ongoing support i.e. telephone sessions, newsletters, periodic meetings. Less common, components comprised of peer leadership opportunities; CST; and motivational interviewing.  School Based Interventions: Positive results were mainly noted in relation to BMI, weight, fitness levels, cardiac blood profiles and health behavior changes,  Family based Interventions: Decreased BMI, increased walking lower fat and cholesterol intake, higher fiber intake; improved systolic blood pressure, and improved cardiac blood profile.  Community Based Interventions: Positive results mainly included improved BMI, waist circumference to height ratio; decreased prevalence of metabolic syndrome; improved cardiac blood profiles; diastolic blood pressure; improved diet and increased physical activity; self-esteem, depression, and anxiety; increased health knowledge.  Clinic Based Interventions:  Improvement was noted mainly in dietary intake; BMI; improved insulin sensitivity, glucose control, and adiposity measurement; improved strength, waist circumference, cardiac blood profiles; strength; blood pressure | 10. NR.  11. Follow-up or “continued opportunity” for participants was identified as most effective. Implementing the intervention in a school setting- easily accessible; support from peers an teachers.  12. Transportation to after-school community centers; poor school attendance; funding and training; environmental. | 13. The school nurse has the potential to influence future health prevention of T2 DM.  14**.** More research evaluating the effectiveness of interventions between boys and girls; high school students; and among youth at risk of developing T2 DM is needed. |
| Halterman et al. (2011).  USA.  RCT.  To *“evaluate the impact of the School-Based*  *Asthma Therapy trial on asthma symptoms among urban children with persistent asthma”* (p. 262). | 1. NR.  2. School-Based Asthma Therapy intervention.  3. Direct observation  therapy with preventive asthma medication reduces asthma symptoms in school settings. | 4. Children (aged 3 to 10 years, n=530).  5. Asthma.  6. School.  7. School nurses. | 8. i)The average number of symptom free days every 2 weeks during winter months-assessed by blinded interviews. ii) Number of nights with asthma symptoms, days with activity limitation, days needing rescue medications, and days of missed school.  9. Participants receiving preventive medications in the school setting had significantly more; symptom-free days (adjusted difference=0.92 days per 2 weeks; 95% CI, 0.50-1.33); fewer nighttime symptoms; less rescue medication use; fewer days with limited activity (all P.01); exacerbation requiring treatment with prednisone (12% vs 18%, respectively; relative risk=0.64; 95% CI, 0.41-1.00) than that of the children in the control group.  Positive intervention effects seen in participants with smoke exposure (n=285; mean symptom-free days per 2 weeks: 11.6 for children in the treatment group vs 10.9 for those in the control group; difference=0.96 days per 2 weeks; 95% CI, 0.39-1.52). | 10. NR  11. NR  12. Family stress; poverty; poor access to care; difficulties in communication | 13. This School-Based Asthma Therapy program has proven to significantly improve persistent asthma symptoms and decrease exacerbations amongst urban children than that of the children in the control group.  14. Further efforts are now needed to estimate the costs of this intervention and to progress forward with dissemination. |
| Kinter et al. (2014).  USA.  RCT.  To “*evaluate the effectiveness of Staying*  *Healthy–Asthma Responsible & Prepared, an academic asthma health education*  *and counseling program, on fostering the use of effective asthma self-care*  *behaviors”* (p.62) | 1. NR.  2. Staying Healthy–Asthma Responsible & Prepared (SHARP).  3. Addresses psychosocial, behavioral, and quality of life in asthma patients. | 4. Caregivers of diagnosed asthma students; Students (dyads, n=216).  5.Asthma.  6. Schools.  7. School nurses. | 8. Episode management; risk reduction/prevention; health promotion.  9. SHARP- the SHARP main effect was significant compared to the control group (t[151] = 2.77, p = .006) i.e. SHARP participants showed increased use of symptom management techniques; use of pillow (t[115] = 5.34, p < .001) and mattress (t[121] = 2.24, p = .027) protectors showed statistical significance i.e. SHARP participants increased use of protectors at posttest  An increase was noted from pre- to post intervention for all participants in relation to 7-9 hours undisturbed sleep, which continued to increase over time (t[148] = 2.12, p < .036). | 10. NR  11. NR  12. NR | 13. Collaborative work with school teachers and nurses may increase the use of asthma self-care behaviors.  14. The use of SHARP in school and community setting can be led by pediatric nurse specialist’s utilsing interdisciplinary teams. This may in turn increase the use of: effective episode management; risk reduction/prevention; and health promotion self-care behaviors that impact asthma control. |
| Pbert et al. (2013).  USA.  RCT.  To *“evaluate the feasibility and efficacy of a school nurse-delivered intervention in improving diet and activity and reducing BMI among overweight and obese adolescents”* (p.1) | 1. NR.  2. “Lookin' Good Feelin' Good”- School nurse-delivered counseling intervention.  3. Involves 6 one-on-one counseling sessions conducted over 2 months; 5-3-2-1-0 approach involving five key behavior changes. | 4. Adolescents (n=82; mean age 15.8 years).  5.Overweight/ Obesity.  6. School.  7. School Nurse. | 8. Continuous outcomes - physiological measures, physical activity, diet, and psychosocial measures; Dichotomous outcomes- diet, activity behaviors.  9. Physiological- Small favorable effect in waist circumference (Adjusted mean change; 95% CI ;( −1.10 (−4.34, 2.13).; systolic BP (Adjusted mean change; 95% CI ;( −2.93 (−8.14, 2.29) at 6 months only  Physical activity- No effect  Diet- A significantly lower amount of total sugar (difference = −45.79g/day; 95% CI −88.34, −3.24) and added sugar (difference =  −51.35g/day; 95% CI −92.45, −10.26) was consumed by the intervention group at 2 months.  Psychosocial  Statistical significance was seen in students who were in involved in the intervention as identified the school nurse as helpful in the understanding of “how to eat healthy” and be “physically active”, (98% vs. 71% of visits, respectively; p = .003); those in the counselling intervention felt more comfortable in discussing with the nurse their weight-related behaviours (88% vs. 62% of visits in intervention vs. control schools respectively; p = .01). | 10. Nurses reported: no extra hours were needed in their work schedule to deliver this intervention; two nurses reported hiring additional cover staff; no issue with lack of time; not difficult to incorporate in to daily schedule.  11. School nurses are in a greater position to provide weight management interventions.  12. NR. | 13. Findings from this study showed that it is feasible for a school nurse to provide this counseling intervention for overweight and obese adolescents; students encouraged the intervention being provided by a school nurses; and it has shown to improve selected self-reported obesogenic behaviors, but not BMI.  14. Future research should be conducted on expanding the counseling intervention in a school setting so that it includes; additional visits extended over a longer period of time; opportunities to engage in physical activity  The need for a “feasible, cost-effective behavioral interventions” has been well documented and the school nurse is in a positive position to provide this intervention to adolescents in an easily accessible location. |
| Cicutto et al. (2013).  USA.  RCT.  “*To evaluate a school-based, multifaceted asthma program that targets students with asthma, and the broader school community*” (p 876). | 1. NR.  2. School based asthma management program.  Both control and intervention group received their usual asthma care from clinics and doctors.  3. Intervention involved:  a) Roaring Adventures of Puff (RAP) interactive asthma self-management education programme for children. Delivered by PHNs and certified asthma instructors. Included education on self-monitoring, inhaler technique, triggers, medications, symptom recognition, asthma action plan, lifestyle and exercise, and managing asthma attacks.  b) Creating Asthma Friendly Schools Resource Kit for broader community. | 4. Children from grades 1 to 5 and their families (n=1316 children + family members n= unspecified).  5. Asthma.  6. Elementary school.  7. Public Health Nurses trained as RAP instructors. | 8. The reported use of urgent care or unscheduled visits to the emergency department, physician office, walk-in clinics, or community clinics related to asthma for the 1-year period following study enrolment; School absenteeism; Days of interrupted activity: Quality of life.  9. Fewer children in the intervention group had a school absence (50% vs 60%; p<.01), required urgent care for asthma (41% vs 51%; p<.0001), or reported a day of interrupted activity (51% vs 63%; p<.01), and had improved quality of life (5.8Å}1.2 vs 5.4Å}1.4; p<.0001). Schools in the intervention group were more likely to have practices supporting an asthma-friendly environment.  Inhaler training was associated with improved inhaler technique in the intervention group. | 10. NR.  11. Parental involvement.  Resource kit requires support for implementation, by public health nurses. Use of Kit increased by meetings with principals/ assistant principals, in-service training workshops about asthma, asthma education folder for staff, hard copies of Resource Kit accessible, school assembly about asthma, review and guidance of school policy.  12. Scheduling of activities within school, engaging parents, shifting school practices regarding medications in school, environmental and trigger management strategies were costly. Difficulty obtaining parent’s signatures on document for child to carry medication at school. | 13. The Public Health School Asthma Project  resulted in significant improvements for the  school and the individual with asthma. Benefits derived extend from improving the school environment to reducing the personal morbidity imposed by asthma. This project demonstrates significant improvements in asthma inhaler skills, quality of life, health services use, asthma-free days, and school absenteeism”.  14. School based asthma education programme can lead to improved self-management of asthma, reduced absenteeism and reduced health service use.  Continued assessment and coaching is required to achieve and sustain accurate inhaler technique. |
| Pbert et al. (2011).  USA.  Cluster-randomized controlled trial.  To *“evaluate the effectiveness of a school nurse– delivered*  *smoking-cessation intervention in increasing abstinence among adolescent smokers”* (p.926) | 1. NR.  2. “5 A’s” model for  adolescents.  3.The model includes asking about tobacco use; advise users to quit; assess willingness to make a quit attempt; assist in their cessation attempt; and arrange follow-up to support their efforts. Two 30-minute sessions were completed before the quit date; a further two 15-minute sessions were completed after the quit date. | 4. Adolescents (n=35 schools, n=1,068 participants).  5. Smokers.  6. School.  7. School nurse. | 8. Primary outcomes: Students reporting not smoking in the past 30 days- assessed at 3 and 12 months; cotinine validated and imputed abstinence when cotinine was missing.  Secondary outcomes: outcome expectations, adjuncts to quit, helpfulness  of nurse, helpfulness of written materials, pharmacotherapy, and nicotine use  9. Compared with the control group participants were almost twice as likely to be abstinent when self-report at three months (odds ratio: 1.90 [95% CI: 1.12–3.24]; P .017)- this difference was determined by quit rates in the male students (15.0% [intervention] vs 4.9% [control]; odds ratio: 3.23 [95% CI: 1.63– 6.43]; P.001); no intervention effect in female students (6.6% vs 7.0% at 3 months and 16.6% vs 15.5% at 12 months); no intervention effect in male students at 12 months (13.9% vs 13.2%). A significant decrease in the amount smoked and frequency was noted in the intervention group compared with control schools (only at 3; not 12 months) | 10. Accessibility of school nurses reduces cost.  11. School settings were identified as a characteristic of successful programme implementation.  12. NR. | 13. The school nurse– delivered smoking-cessation intervention demonstrated its efficiency and feasibility in improving abstinence in adolescent boys (short-term) and reducing frequency and amount in both genders (short term).  14. Additional research is required to improve adolescents stopping and abstaining from smoking. |
| Wright et al. (2013).  USA.  RCT.  To *“evaluate the impact of a nurse directed, coordinated, culturally sensitive, school-based, family-centered lifestyle program on activity behaviors and body mass index”* (p.1) | 1. NR.  2. Intervention: Kids N Fitness©  3. Intervention: A 6-week program for parents and children which includes: 45 min of structured physical activity; 45 min nutrition education class; plus environmental activities. | 4. Students (n = 251; aged 8-12 years).  5. Enhance physical activity behavior/reduce BMI.  6. School.  7. School Nurse. | 8. Anthropometric measures; Health behaviors/knowledge  9. Anthropometric measures: Female students (KNF group; BMI (p = 0.047) and BMI z-score (p = 0.05) decreased from baseline to 4 months; sustained till 12 months).  Health behaviors/knowledge: Significant differences between the KNF and control group for both genders were seen in three distinct areas: daily physical activity; PE class; attendance; TV viewing; Both genders had increased participation in 60 min of activity per day (from baseline to 4 months)-effects remained constant (at 12 months) for males (p = 0.002) and females (p = 0.005); Both genders had increased attendance in PE class (from baseline to 4 months)-effect were sustained for males (p = 0.003) and females (p = 0.002); A significant decrease in males and females was noted in relation to viewing TV (from baseline to 4 months)- effects remained constant at 12 months for males only (p = 0.030). | 10. Authors state that the delivery of school based programs can be provided as low cost/minimal cost to families; low-income; urban children (p3). Furthermore utilising nurses is a cost effective opportunity in low income school based programmes.  11. Involve stakeholders across school, home, and environmental contexts; formation of a community advisory board and school-health advisory council i.e. school and community partners.  12. NR. | 13. Nurses are pivotal to the implementation of such interventions due to their knowledgeable nature and expertise in identifying the needs of overweight and obese children; male and female students can improve their physical activity by becoming involved in culturally appropriate school-based interventions.  14. There is a need for gender-focused activities to assist in reducing incidences of overweight adolescents and chronic disease management. Nurses play a key collaborative role in implementing such interventions in school settings. |
| **Pregnancy/ Postnatal** | | | | | |
| Brugha et al. (2011).  UK  Cluster RCT  To test if receiving care from a HV trained in identification and psychological  intervention methods prevents depression 6–18 months postnatally in women who are not depressed 6 weeks postnatal women scoring <12 on the postal EPDS at 6 weeks postnatally | 1 NR  2. Systematic assessment of depressive symptoms in women by trained Health Visitors who established warm, therapeutic relationships using a cognitive behavioural approach or person centred approach.  3. 1 hr visit a week for max 8 weeks commencing around 8 weeks postnatally. Control – usual care. | 4. Postnatal women. (n= 2241).  5. PN mental health/ prevention of depression.  6. Primary care teams.  7. Health visitors trained in requirements for this study. | 8. EPDS, CORE-OM, SF-12, State anxiety at 6 mths, 12 mt, 18 mt.  9. Identification and psychological  intervention prevents depression 6–18 months postnatally in women who are not depressed  OR for EPDS >12 at 6 months was 0.71 [95% CI 0.53–0.97, p=0.031] for intervention group (IG) compared with control (CAU) group. Two subgroups compared for 6-week EPDS score of 6–11 (n=999) and a ‘ lowest severity ’ subgroup with a 6-week score of 0–5  (n=1242). No difference in psychological effectiveness by subgroup (interaction term: z=x0.28, p=0.782). | 10. 8 days training for HVs.  11. Intervention by trained HVs provides a universal, enduring preventive effect for depression in women who screen negative for depression postnatally.  12. Differences in practice environment and culture reported. | 13. Potential benefits for increased visits and focus on maternal psychological wellbeing rather than physical welfare of child. Cost effective intervention/  14. The study provides some evidence of the impact of a depression prevention programme to provide clinically significant, useful and persistent reductions in the prevalence of depression in postpartum women. This requires further independent evaluation. |
| Cooper et al. (2015).  UK.  RCT.  To investigate if an intervention enhancing the mother-infant relationship would prevent PND (postnatal depression), impairments in parenting and adverse effects on child development. | 1. NR.  2. Supportive home visits (2 antenatal + 9 postnatal).  3. Measures to enhance maternal sensitivity to infant communicative signals, including items from the Neonatal Behavioral Assessment Scale. | 4. Pregnant women at risk PND. (n=190: IG 91, CG 99)  5. PND, maternal attachment and infant development.  6. Primary care.  7. Health Visitors | 8. Maternal mood, maternal sensitivity in mother–infant engagement, and infant behaviour problems, attachment and cognition at 8 wks, 18 wks, 12 and 18 mts postpartum.  9. No impact on maternal mood, quality of maternal parenting behaviours, infant outcome. Some measures indicated that those with a lower level of antenatal risk experienced benefit but results not consistent or strong.  None of the results were statistically significant | 10. Training of HVs on intervention and resource cost of additional visits.  11. Mothers reported intervention to be of considerable emotional and practical support and help in enhancing their appreciation of their infant’s abilities and their ability to communicate with their infants.  12. NR. | 13. Objective findings conflicted with mothers’ report that intervention provided considerable emotional and practical support including ability to communicate with their infants.  14. This approach to preventing PND and its associated problems not recommended. |
| Cristie & Bunting (2011)  UK NI  Cluster RCT  To determine the effect of frequency of HV’s home visits on ‘low-risk’ first-time mothers outcomes at 8 weeks and 7 months postpartum.  (195 intervention 159 control)  ROC | 1. NR  2. Postnatal home visits by HVs  3. Weekly home visits by HVs to 8 weeks postpartum or usual care of one PN visit at 8-10 days. 80 HVs in intervention group | 4. Low risk first-time postnatal women (n=295)  5. Parenting, maternal wellbeing and service use  6. Primary health care setting  7. Health visitors – 39 delivered intervention of weekly visits no of visits m = 6, control m = 2 | 8. ‘Maternal wellbeing’ (EPDS)  Parenting stress index (role restriction)  Maternal physical health  ‘Baby nurture’ –  ‘Coping/adapting resources’  Self-efficacy (PES)  Assessed at recruitment (8-10 days) and 8 weeks and 7 months  9. Variable effects – increased service satisfaction and decreased use of emergency medical services. EPDS higher in intervention group than control at 8 wks but not 7 mths. | 10. Extra visits have resource implications  11. NR  12. Variation among HVs noted, also trial did not alter the content of care provided. | 13. Increasing the number of home visits increased satisfaction and reduced use of emergency medical services, but had no effect on parenting outcomes. More intervention mothers had raised EPDS at 8 weeks but no difference at 7 months.  14. Main outcome measure was depression and this study did not demonstrate a clear benefit for women from the increased visits by HVs related to their EPDS score |
| Dodge et al. (2013).  USA.  RCT.  Evaluation of home nursing programme effectiveness at reducing infant emergency care (birth to 12 months). | 1. NR.  2. Preventive system of care model 3 to 7 contacts home/ community/ phone using scripted intervention between 3- 12 wks, brief intervention on targeted areas.  3. Nurse engages parents and provides brief educational interventions  organized as 20 “teaching moments” and assesses and scores health and  psychosocial risk in each of 12 domains. | 4. 531 families.  5. Emergency medical care of infant and overnight hospital stays.  6. Community service.  7. Nurses trained in programme. | 8. Infant emergency medical care and overnight hospital stays in first 12 months.  9. Identification of family needs and linking with community resources. | 10. $700 cost per family offset by savings in hospital medical care costs before the infant’s first birthday.  11. Identifying needs and linking families to appropriate community resources.  12. Not identified. | 13. Preventive impact occurs through the nurse home visitor’s success in identifying individual family needs, intervening briefly to address those needs when risk was moderate, and connecting the family with targeted community resources to meet those needs for families having higher risk.  14. Universal/population based community intervention affective in linking families with appropriate community services and reduces emergency medical care. |
| Dodge et al 2014  USA  RCT- further reporting on RCT by Dodge et al (2013)  Evaluation of a postnatal home nursing programme on access to emergency health care (birth to 6 months) and other family wellbeing outcomes | 1 Nurses trained in intervention  2 Preventive system of care model 4 to 7 contacts home/ community/ phone using scripted intervention between 3- 12 wks, brief intervention on targeted areas  3. brief interventions (20 “teaching moments,”), assessment health and psychosocial risk 12 domains | 4. Evaluation study 531 families/births  In n =269 Cnt n = 280  5. Emergency medical care, and family wellbeing  6. Community service  7. Nurses trained in programme | 8. Infant emergency care episodes, community connections, positive parenting (Mother-Child Neglect Scale, Parent-Child Conflict Tactics Scales, Knowledge of Infant  Development Inventory, Parenting Sense of Competence Scale, Survey of New Parents, Duke Endowment Child Abuse Prevention Initiative Neighborhood Survey behaviors, EPDS, Generalized Anxiety Disorder questionnaire, CAGE and CAGE-AID questionnaires, participation in quality out-of-home child care  9. Universal structured home visiting programme tailored to individual needs benefits families and is cost effective. | 10. $700 cost per family offset by savings in medical care costs  11. Emergency medical care, overnight hospital stays, access to community services, positive parenting behaviours, home environment quality, maternal mental health  12. Not identified | 13. Structured home visits provides a feasible, and effective public health policy for families of newborn infants including effective triage to community services  14. Universal service which is effective in assisting families access community services |
| Lopez et al. (2015)  USA  Cochrane Systematic Review  To assess the effectiveness of postpartum educational interventions on contraceptive use. | 1. NR.  2. Nurses, midwives (and physicians) postpartum educational interventions on contraceptive use.  3. Included individual or group counselling, written materials, video or audio recordings | 4. 12 trials, 4145 women – USA, Australia, Nepal, Pakistan, and Syria.  5. Unplanned pregnancy and contraceptive use.  6. Postpartum hospital or 2-3 wks later (home/clinic/ phone).  7. Nurses, midwives. | 8. 2^nd^ birth (by 24 mts). Contraception use at 6mts, effective contraception at 6mths.  Adolescents, with homebased mentoring had fewer 2^nd^ births within 2 years compared to the control group (OR 0.41, 95% CI 0.17 to 1.00). The other five interventions had no effect. Of trials with lower quality evidence, two showed some effectiveness. In Nepal, women with an educational session immediately postpartum were more likely to use contraception at six months than those with a later or no session (OR 1.62, 95% CI 1.06 to 2.50). In an Australian study, teenagers in a structured home-visiting program were more likely to have effective contraception use at six months than those with standard home visits (OR 3.24; 95% CI 1.35 to 7.79).  Meta analysis not done due to varying study designs  9. NR | 10. Given the associated costs and logistics, some programs would not be feasible in many settings.  11. NR.  12. NR. | 13. Insufficient evidence to recommend any particular educational interventions on contraceptive use.  14. Although half of the interventions were effective in reducing repeat pregnancies or births and increasing contraceptive use, the overall evidence of effectiveness was of low to moderate quality. Two trials of counselling providing one or two sessions were effective but were limited by self- reported outcomes and short term outcome assessment. Three trials of multifaceted programmes involving multiple contacts were effective but their applicability in other locations is uncertain and health care providers would need to consider which intervention might be appropriate for their setting and level of resources.  Valid and reliable outcome measures are needed to obtain meaningful results. |
| Mirmolaei et al (2014)  Iran  RCT  To compare effect of two midwife home visits to usual care on healthy behaviours of low‑risk mothers | 1. NR  2. Attendance at primary care facilities compared with home visits by midwives.  3. First visit at health centre (3-5 days) followed by two postnatal home visits at 10-15 days and 2^nd^ within 42-60 days | 4. Postpartum women (n= 200)  5. Maternal health behaviours assessed at 60 days (2 months)  6. Home visits in designated geographical region of Iran  7. Midwives | 8. Impact of two postnatal home visits on the healthy behaviors of low risk mothers: included maternal behaviors in relation to nutrition, physical activity, smoking, alcohol, breastfeeding, FP, personal and mental health, and ability to taking care of newborn.  Intervention group more likely to receive postpartum care (P < 0.001).  Mean score of maternal healthy behaviors increased from 120.5 (SE = 0.76) to 148.9 (SE = 1.02) (P < 0.001) with control group 119.9 (SE = 1.06) to 140.9 (SE = 1.08) (P < 0.001).  Mean score of maternal healthy behaviors in the intervention group had significant differences with that in the control group (P < 0.001).  9. Poor uptake for standard care | 10. Additional cost of home visits a factor for developing countries.  11. NR  12. Cost of setting up home visiting service for low risk mothers in developing countries when health centre available. | 13. Postpartum care at home is effective in improving maternal healthy behaviors such as breastfeeding and family planning.  14. Costs of home‑visits and low rates of receiving routine care in developing countries such as Iran, the cost effectiveness assessments of other methods forpostpartum care can enable health policymakers. |
